# HMGCS2 serves as a potential biomarker for inhibition of renal clear cell carcinoma growth

**DOI:** 10.1038/s41598-023-41343-7

**Published:** 2023-09-05

**Authors:** Huajie Mao, Runzhi Wang, Fengling Shao, Ming Zhao, Dayu Tian, Hua Xia, Ya Zhao

**Affiliations:** 1 Department of Laboratory Medicine, The First Affiliated Hospital of Northwest University, Xi’an No.1 Hospital, Xi’an, 710002 China; 2 The Ministry of Education Key Laboratory of Laboratory Medical Diagnostics, the College of Laboratory Medicine, Chongqing Medical University, Chongqing, 400016 China; 3 Department of Science and Education, The First Affiliated Hospital of Northwest University, Xi’an No.1 Hospital, Xi’an, 710002 China

**Keywords:** Cancer, Computational biology and bioinformatics

## Abstract

3-Hydroxymethylglutaryl-CoA synthase 2 (HMGCS2) is the rate-limiting enzyme for ketone body synthesis, and most current studies focus on mitochondrial maturation and metabolic reprogramming. The role of HMGCS2 was evaluated in a pan-cancer multi-database using R language, and HMGCS2 was lowly expressed or not differentially expressed in all tumor tissues compared with normal tissues. Correlation analysis of clinical case characteristics, genomic heterogeneity, tumor stemness, and overall survival revealed that HMGCS2 is closely related to clear cell renal cell carcinoma (KIRC). Single-cell sequencing data from normal human kidneys revealed that HMGCS2 is specifically expressed in proximal tubular cells of normal adults. In addition, HMGCS2 is associated with tumor immune infiltration and microenvironment, and KIRC patients with low expression of HMGCS2 have worse prognosis. Finally, the results of cell counting kit 8 assays, colony formation assays, flow cytometry, and Western blot analysis suggested that upregulation of HMGCS2 increased the expression of key tumor suppressor proteins, inhibited the proliferation of clear cell renal cell carcinoma cells and promoted cell apoptosis. In conclusion, HMGCS2 is abnormally expressed in pan-cancer, may play an important role in anti-tumor immunity, and is expected to be a potential tumor prognostic marker, especially in clear cell renal cell carcinoma.

## Introduction

HMGCS2 encodes the protein 3-hydroxy-3-methylglutaryl-CoA synthase 2, which belongs to the HMG-CoA synthase family^1^. It is a mitochondrial enzyme that catalyzes the first irreversible step in ketogenesis. HMGCS2 combines acetyl-CoA to form acetoacetyl-CoA, which is further converted to HMG-CoA by HMG-CoA reductase (HMGCR), and eventually metabolized to acetoacetate^1^. Additionally, HMGCS2 is a key molecule in mitochondrial metabolic reprogramming^2^ and is sensitive to high-fat and high-sugar diets^3, 4^.

Renal cell carcinoma (RCC) is fundamentally a metabolic disease characterized by the reprogramming of energy metabolism^5–8^, particularly the altered flux of glycolysis^9–11^, impaired mitochondrial bioenergetics and oxidative phosphorylation, as well as dysregulated lipid metabolism^9, 12–14^. Furthermore, integrated research using bioinformatics and cell biology can provide new insights into prognostic biomarkers in clear cell renal cell carcinoma (ccRCC)^15, 16^, particularly in relation to metabolism-related genes^17^.Previous experimental data have reported that HMGCS2 participates in various disease processes or the maintenance of body homeostasis by limiting ketone body production, including diabetic nephropathy, colorectal cancer, hepatocellular carcinoma, intestinal stem cell homeostasis, and mitochondrial metabolic reprogramming^2, 18–21^. Additionally, HMGCS2 has been shown to potentially act as a tumor suppressor in various human cancers, such as mediating ketone production and regulating proliferation and metastasis in hepatocellular carcinoma^22, 23^. Low expression of HMGCS2 is known to be associated with adverse clinical and pathological features, including shorter overall survival and disease-free survival^24–26^. Although HMGCS2 is currently considered a potential biomarker and a key mediator in various human malignancies, there is no comprehensive report on its anti-tumor function in pan-cancer.

In this study, we investigated the role of HMGCS2 in pan-cancer through bioinformatic analysis. Furthermore, we validated HMGCS2 as a tumor suppressor in renal clear cell carcinoma (ccRCC) through in vitro experiments, demonstrating that the loss of HMGCS2 leads to malignant cellular behavior in ccRCC. Overall, HMGCS2 represents a promising therapeutic target for cancer and serves as a prognostic marker associated with mitochondrial-related outcomes in renal clear cell carcinoma.

## Method

### Analysis of HMGCS2 expression levels in pan-cancer datasets

We obtained the comprehensive and standardized pan-cancer dataset from the UCSC database (https://xenabrowser.net/, accessed on October 8, 2022), which consisted of a total of 19,131 samples and encompassed 60,499 gene expression profiles, incorporating data from TCGA, TARGET, and GTEx databases. Specifically, we extracted the expression data for the HMGCS2 (ENSG00000134240) gene in each sample. To investigate the expression patterns and cellular localization of HMGCS2 in various organs, we referred to the Human Protein Atlas (HPA) as a valuable resource.

### Identification of correlations between HMGCS2 expression levels and clinicopathological features and human cancer survival

Sanger box (http://vip.sangerbox.com/home.html, accessed on October 8, 2022) is an open-access bioinformatics analysis platform that facilitated the investigation of the correlation between HMGCS2 expression and several clinical indicators. These indicators included patient overall survival (OS), primary tumor status (T), lymph node metastasis (N), distant metastasis (M), tumor grade (G), and tumor stage (Stage). To ensure the reliability of the analysis, samples with an expression level of 0 and a follow-up period of less than 30 days were excluded. Survival analysis was conducted using the Kaplan–Meier method and ROC curve analysis. Furthermore, the correlation between HMGCS2 expression and clinicopathological characteristics was assessed using the "person" method.

### Association between HMGCS2 expression and genomic heterogeneity and tumor stemness in different types of cancer

The Sanger box platform obtained data on simple nucleotide variation and copy number variation from the GDC database (https://portal.gdc.cancer.gov/, accessed on October 8, 2022). The acquired data were then processed using the built-in MuTect2 software and GISTIC software^27, 28^. In line with the study by Thorsson et al., the pan-cancer Tumor Mutational Burden (TMB) data were extracted and subsequently analyzed for correlation with HMGCS2 expression using the Pearson correlation coefficient. Additionally, based on the findings of Thorsson et al., data on TMB, Microsatellite Instability (MSI), tumor purity, ploidy, Homologous Recombination Deficiency (HRD), and Loss of Heterozygosity (LOH) in pan-cancer were extracted to analyze their correlation with HMGCS2 expression. The Pearson correlation coefficient was utilized to determine the correlation^29, 30^.

### Correlation of HMGCS2 expression with immune microenvironment (TME), immune-related genes and RNA modification-related genes in different types of cancer

We employed the immune infiltration calculation method proposed by Kosuke Yoshihara et al. to compute immune-related scores for different tumors, including stromal, immune, and ESTIMATE scores^31^. Furthermore, the Pearson correlation coefficient was utilized to examine the correlation between HMGCS2 expression and these scores. To comprehensively analyze the association of HMGCS2 with immune cell infiltration in pan-cancer, we utilized the Time tool (http://timer.comp-genomics.org/, accessed on October 8, 2022), Epic tool (http://epic.gfellerlab.org/, accessed on October 8, 2022), and quanTIseq tool (https://icbi.i-med.ac.at/software/quantiseq/doc/, accessed on October 8, 2022)^32–34^. Moreover, we extracted 60 immune checkpoint genes^30^, 150 immune pathway genes^35^ and RNA modifier genes in various cancers for further analysis of the relationship between HMGCS2 expression and them.

### Specific expression of HMGCS2 in healthy human kidney

The expression of HMGCS2 in normal human kidneys was analyzed using the UCSC Cell Browser (http://cells.ucsc.edu/, accessed on October 8, 2022). This analysis included two single-cell sequencing datasets obtained from healthy adults. The first dataset, GSE151302, was derived from the study conducted by Muto et al., consisting of 27,034 cells^36^. Another data set comes from Stewart's research, which contains 40,268 mature and 27,203 fetal kidney cells.

### Plasmids construction and cell culture transfection

The coding sequences (CDS) of HMGCS2 (NM_001166107.1) were amplified through in vitro polymerase chain reaction (PCR) using HEK293T cell cDNA. The amplified sequences were then cloned into the CMV-flag vector using the EcoRI and XhoI restriction enzymes. The CMV-GFP-HMGCS2 plasmid was subjected to sequencing and alignment to confirm the successful cloning of the DNA sequence (performed by BGI, Chongqing, China). The OSRC2 cell line was cultured in Dulbecco's Modified Eagle Medium (DMEM) supplemented with 10% fetal bovine serum and 1% penicillin/streptomycin. The cells were maintained in a 37 °C incubator with 5% CO_2_. For transfection, the GFP-CTL or GFP-HMGCS2 plasmids were transfected into SH-SY5Y cells using the Neofect™ DNA transfection reagent (Neofect biotech, Beijing, China) following the manufacturer's protocol. Briefly, OSRC2 cells were cultured in a 35 mm dish with complete medium. The DNA (2 μg) was diluted in Opti-MEM and mixed gently with 2 μL of Neofect™. After incubating for 25 min at room temperature, the transfection mix was added to the plate and further incubated at 37 °C in a 5% CO_2_ environment.

### Western Blot analysis

The GFP-CTL and GFP-HMGCS2 plasmids were transfected into OSRC2 cells with Neofect™ DNA transfection reagent. After cells were transfected for 48 h, the cells were lysed by 1% SDS lysing buffer containing Protease Inhibitor Cocktail and Phosphatase Inhibitor Cocktail (Apexbio, Houston, TX, USA) for Western blot analysis. The protein concentration was determined by a BCA protein assay reagent kit (Thermo Scientific, Waltham, MA, USA). All blots were, respectively, incubated with primary anti-bodies anti-flag (1:1000, ABclonal, Wuhan, China), anti-p-ACC, anti-ACC, Anti-p-ACLY, anti-ACLY, anti-PFKFB3, anti-HK2 and anti-LDHA (1:1000, Cell Signaling Technology, MA, USA), anti-ALDOA, anti-ENO2, anti-HADH and anti-PPARA (1:1000, proteintech, Wuhan, China), and anti-β-Tubulin (1:5000, TRANSGEN, Beijing, China). Bands were visualized with ECL Reagents (Smart-Lifesciences, Changzhou, China).

### Cell viability assay

After cells were transfected for 48 h, the cell counting kit-8 (CCK-8) assay kits (MedChem Express, NJ, USA) were used for cell viability analysis. The GFP-CTL or GFP-HMGCS2 cells were seeded in 96-well plates at a density of 2 × 104 cells per well and cultured for 0 h, 24 h, 48h and 72 h. Then the DMEM medium (100 μL) and CCK8 solution (10 μL) were added to each well and incubated for 1 h. Finally, the absorbance of each well was measured at 450 nm using a microplate reader (Thermo Scientific, Waltham, MA, USA). A two-tailed paired or unpaired t-test statistical analysis was performed using GraphPad Prism 8 software (GraphPad, San Diego, CA, USA).

### RNA extraction and q-PCR

Real-time quantitative PCR (RT-qPCR) was performed following the methodology described by Xie et al. Specifically, total RNA was extracted from cells using TRIzol reagent (Invitrogen) according to the manufacturer's instructions. For each sample, cDNA synthesis was performed using the iScript cDNA Synthesis Kit (Bio-Rad) in a 20 μL reaction system with 1 μg of total RNA. A 25 μL PCR reaction was then carried out using 1 μL of the cDNA library for each sample. Fast SYBR Green Master Mix (Bio-Rad) was used, and the threshold cycle (Ct) values for each sample were determined using the CFX96 Real-Time PCR Detection System (Bio-Rad). The 18S gene served as the reference gene for normalization in these studies. The relative expression levels of the target gene were calculated using the 2^-ΔΔCt method (ΔCt = Ct of 18S—Ct of target gene). The primer sequences used as follows: HMGCS2 F: AGGAGGCCAATCCATACAACCA, R: TGGGGAAGGTCTGTATCCCT; 18S F: GTAACCCGTTGAACCCCATT, 18S R: CCATCCAATCGGTAGTAGCG.

### Colony formation assay

Long-term cell survival was monitored in a colony formation assay. In brief, 1000 cells were seeded into 6-well plates and allowed to grow for 2 weeks. The cells were fixed with 4% paraformaldehyde for 15 min and visualized by 0.5% (w/v) crystal violet (Sigma-Aldrich) staining. Colons in the plate were scanned using Odyssey Scanner (LI-COR, Lincoln, NE, USA) and the number of colons was quantified by Image J software.

### Flow cytometry analysis

When convergence is 80–90%, harvest OSRC2 cells overexpressing GFP-Con or GFP-HMGCS2 with EDTA-free trypsin. Apoptosis was measured by Annexin V-fluorescein isothiocyanate (FITC) apoptosis detection kit (KeyGEN BioTECH, Nanjing, China). Then, single cells were analyzed by flow cytometry according to the manufacturer's instructions. Apoptosis detection was performed by the College of Life Sciences, Chongqing Medical University.

## Results

### HMGCS2 is differentially expressed between tumor tissues and normal tissues

**Figure 1:**
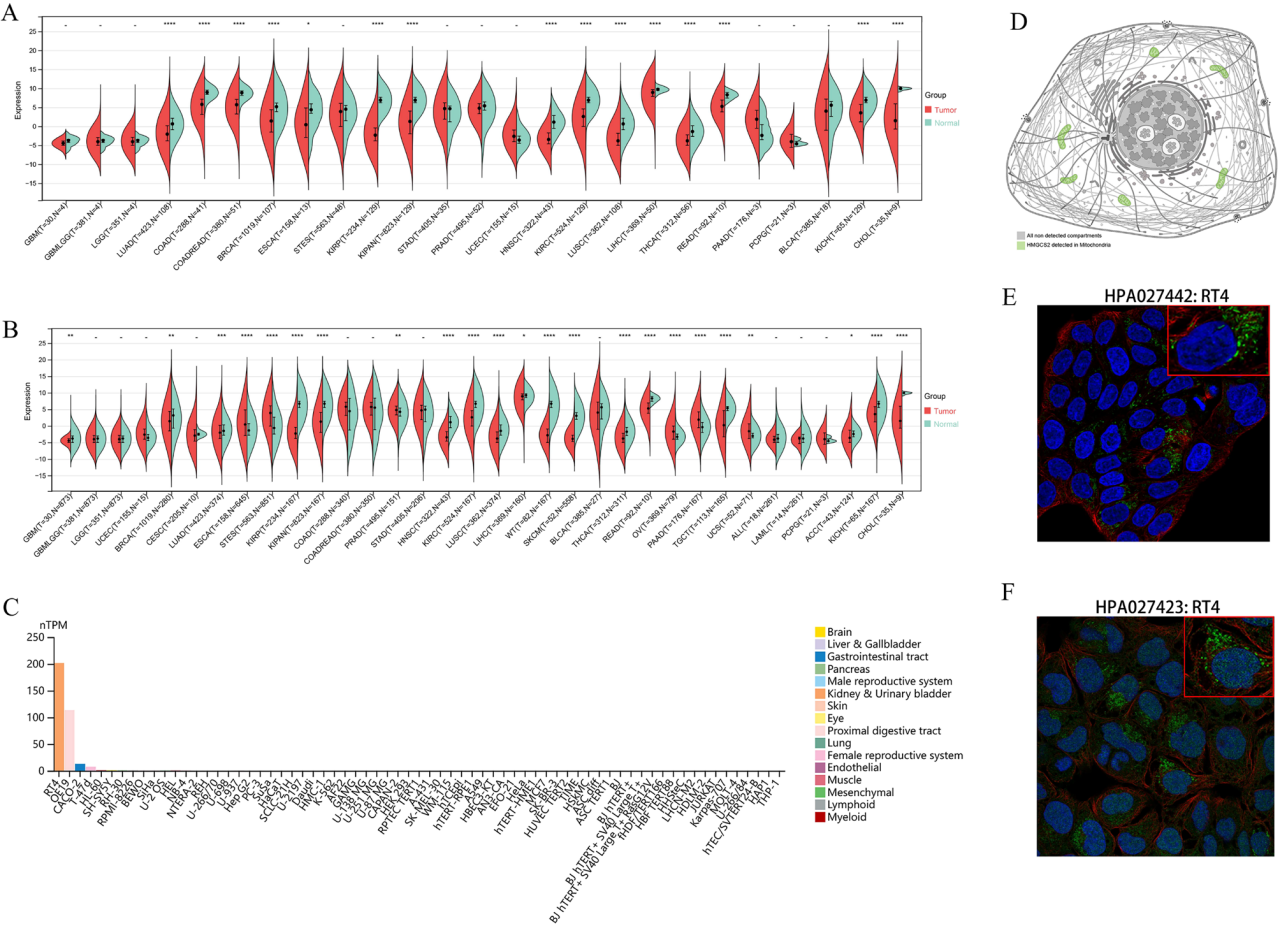
Pan-cancer analysis of HMGCS2 expression. (**A**,**B**) Differential expression of HMGCS2 between tumor and normal tissues (TCGA (**A**), TCGA + GTEx + TARGET (**B**). (**C**) Expression of HMGCS2 in various cancer cell lines. (**D**–**F**) Cellular localization of HMGCS2 (p < 0.05). *p < 0.05; **p < 0.01, ****p < 0.0001.

From the normalized pan-cancer datasets of TCGA, TARGET, and GTEx, we obtained a total of 19,131 normal tissue samples and 60,499 tumor samples. Specifically, we evaluated the differential expression of ENSG00000134240 (HMGCS2) between normal and tumor samples in different tumor types. HMGCS2 was significantly downregulated in 15 tumor types within the TCGA database (Fig. 1A), including LUAD, KIRC, and LIHC (P < 0.05). Furthermore, upon combining the TARGET and GTEx databases, we observed upregulation of HMGCS2 in ESCA, STES, PRAD, OV, PAAD, and UCS (Fig. 1B). In the HPA database, HMGCS2 exhibited significantly lower expression levels in most cell lines, except for RT4 (human bladder transitional cell papilloma cells) and OE19 (human esophageal cancer cells) (Fig. 1C). Additionally, through immunofluorescence staining using different antibodies, we confirmed that HMGCS2 primarily localizes within the mitochondria (Fig. 1D). These results indicate significant downregulation of HMGCS2 in the majority of tumors and suggest a potential association with mitochondrial function.

### The prognostic value of HMGCS2 in pan-cancer

**Figure 2:**
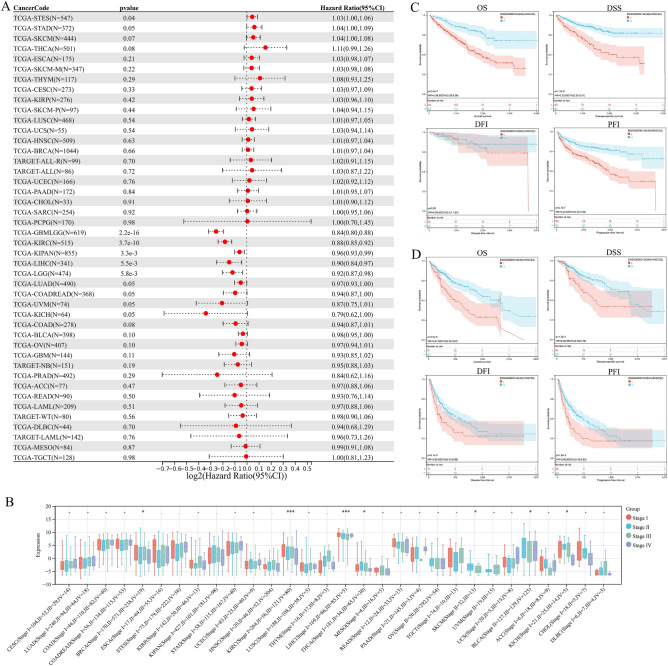
Pan-cancer survival analysis of HMGCS2. (**A**) Forest plot showing the correlation between overall survival (OS) and HMGCS2 expression in various cancers. (**B**,**C**) K-M analysis of associations between HMGCS2 expression and OS, DSS, DFI and PFI in KIRC (**B**) and LIHC (**C**).

To assess the clinical relevance of HMGCS2 expression, we investigated its relationship with overall survival (OS), primary tumor status (T), lymph node metastasis (N), distant metastasis (M), tumor grade (G), and tumor stage. Univariate Cox analysis revealed significant associations between HMGCS2 expression and overall survival in STES, GBMLGG, KIRC, KIPAN, LIHC, and LGG (P < 0.05) (Fig. 2A). HMGCS2 expression was also correlated with primary tumor status (T) in BRCA, ESCA, STES, KIPAN, KIRC, LIHC, and CHOL (P < 0.05) (Supplementary Fig. 1A). Furthermore, lymph node metastasis (N) was associated with HMGCS2 expression in BRCA, STES, KIPAN, STAD, HNSC, KIRC, and THCA (P < 0.05) (Supplementary Fig. 1B). Distant metastasis (M) showed correlations with COADREAD, KIPAN, and KIRC (Supplementary Fig. 1C). Tumor grade (G) was associated with HMGCS2 expression in STES, KIPAN, HNSC, KIRC, and LIHC (Supplementary Fig. 1D). Moreover, HMGCS2 expression was related to tumor stage in BRCA, KIRC, LIHC, THCA, SKCM, BLCA, and KICH (Fig. 2B), Furthermore, Kaplan–Meier survival analysis was performed to assess the association of HMGCS transcription levels with KIRC and LIHC. Interestingly, the transcription levels of HMGCS2 were found to be correlated with overall survival (OS), disease-specific survival (DSS), and progression-free interval (PFI) in KIRC patients, but not with disease-free interval (DFI) (Fig. 2C). In LIHC, HMGCS2 was found to be associated with all prognostic parameters (Fig. 2D). These results further highlight the prognostic potential of HMGCS2 in KIRC and LIHC (Supplementary Fig. 1B,C).

### Correlation of HMGCS2 expression with genomic heterogeneity and tumor stemness in various cancer types

**Figure 3:**
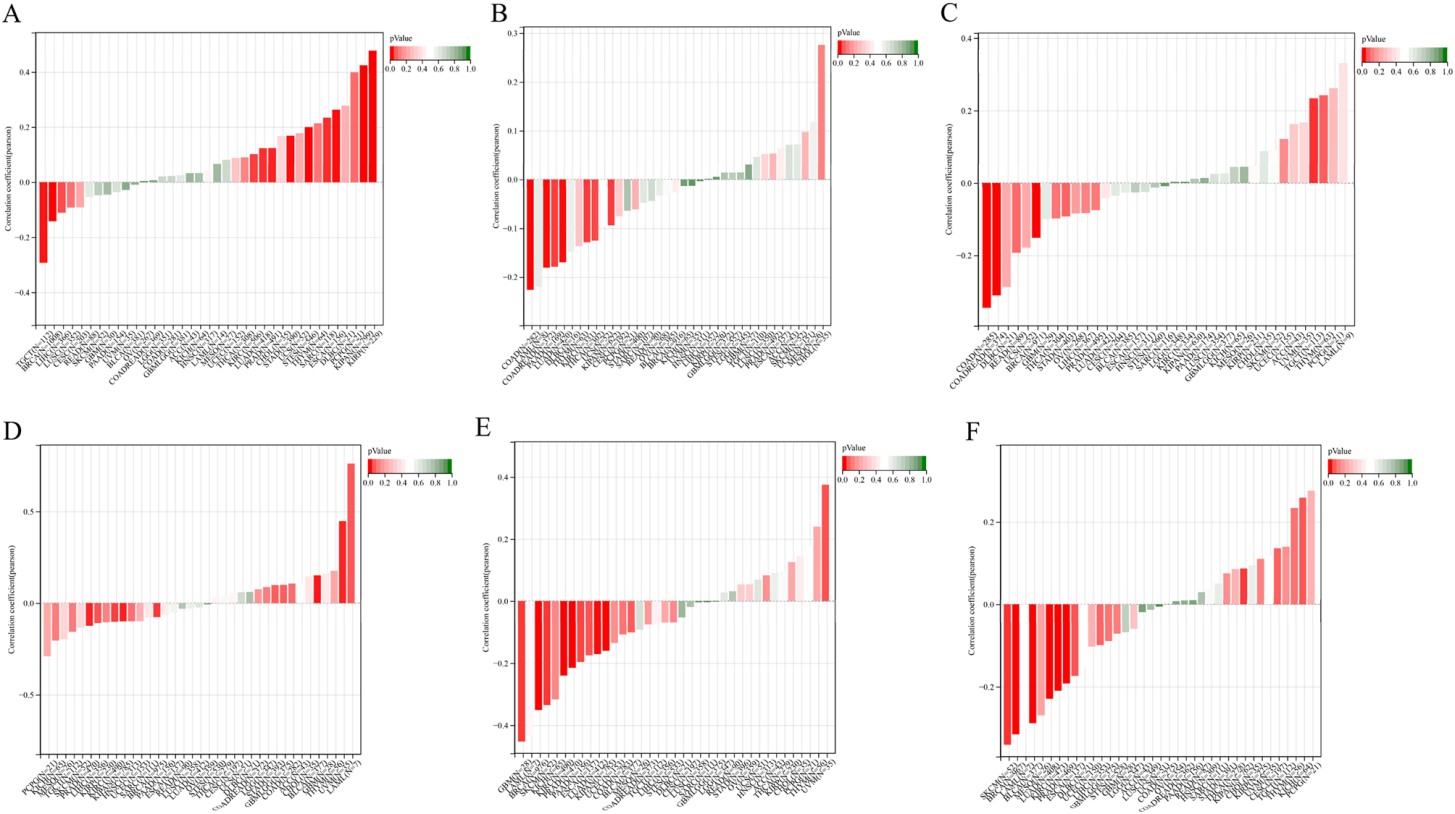
HMGCS2 expression correlates with tumor stemness and genomic heterogeneity. (**A**) Association of tumor stemness with HMGCS2 expression in various cancers. (**B**–**F**) HMGCS2 expression and Tumor mutational burden (**B**), microsatellite instability (**C**), Ploidy (**D**) Homologous recombination deficiency (**E**), Loss of Heterozygosity (**F**) in various cancers.

Cancer stem cells (CSCs) represent a distinct population within tumors and play a crucial role in cancer development^37^. Thus, we investigated the association of HMGCS2 with tumor stemness. Using an mRNA signature-based approach, we calculated the EREG.EXPss tumor stemness score^38^ and observed significant correlations in 12 tumors. Notably, nine tumors including LUAD, ESCA, STES, SARC, KIRP, KIPAN, STAD, PRAD, and KIRC exhibited a significant positive correlation, while three tumors, namely BRCA, LIHC, and TGCT, showed a significant negative correlation (P < 0.05) (Fig. 3A).

Tumor mutational burden (TMB), microsatellite instability (MSI), ploidy, homologous recombination deficiency (HRD), and loss of heterozygosity (LOH) are factors contributing to tumor development and therapy resistance^39–43^.

Specifically, we observed a positive correlation between HMGCS2 expression and ploidy in LAML, UVM, and BLCA, while negative correlations were evident in BRCA, KIPAN, PRAD, KIRC, and LIHC. Regarding HRD, HMGCS2 showed a positive correlation with UVM and a significant negative correlation in GBM, LUAD, BRCA, ESCA, KIPAN, PRAD, KIRC, PAAD, and SKCM. Furthermore, we found a significant association between HMGCS2 expression and LOH in nine tumors. HMGCS2 exhibited a positive correlation with KIPAN and KICH, while a negative correlation was observed in LUAD, BRCA, ESCA, PRAD, KIRC, SKCM, and BLCA. Overall, our results highlight the strong association between HMGCS2 expression and tumor genomic heterogeneity and stemness, particularly in KIRC.

### Correlation between HMGCS2 Expression and the TME in Different Types of Cancers

**Figure 4:**
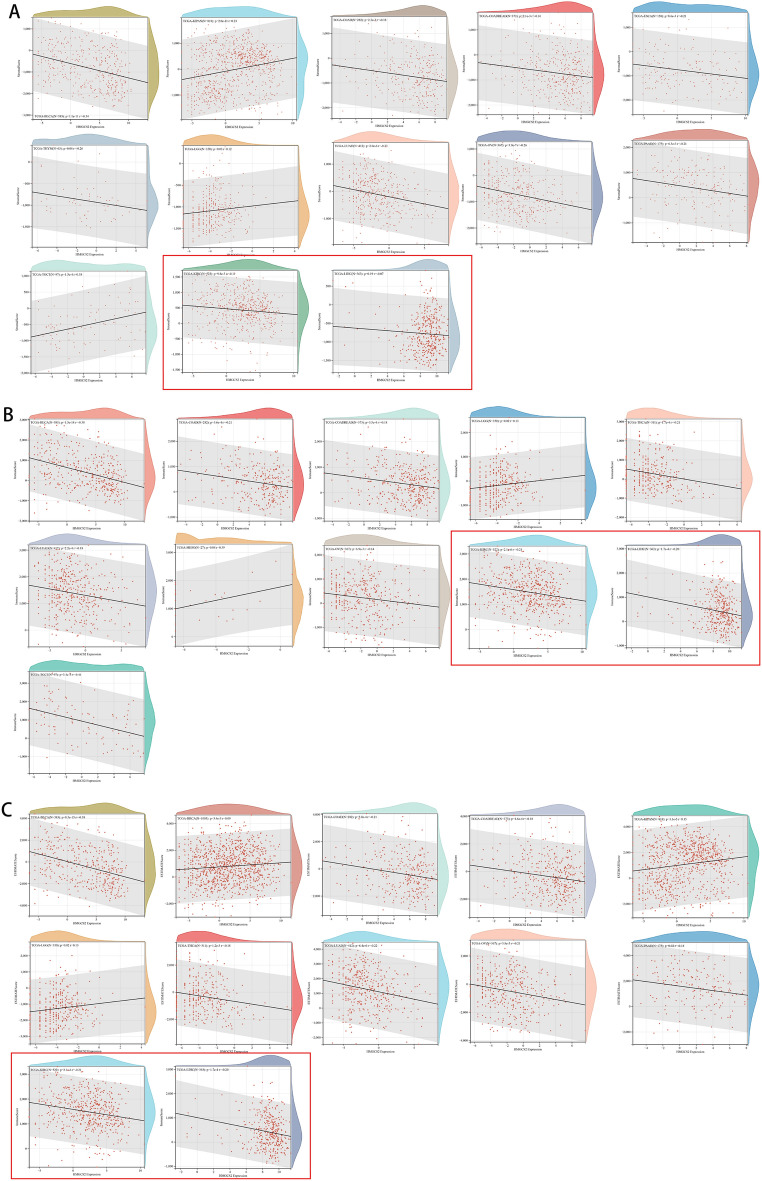
Pan-cancer immune infiltration analysis of HMGCS2. (**A**) Association of HMGCS2 with stromal score in pan-cancer. (**B**) Association of HMGCS2 with immune score in pan-cancer. (**C**) HMGCS2 association with estimate in pan-cancer.

The tumor microenvironment (TME) plays a crucial role in tumorigenesis and cancer progression, prompting the investigation of therapeutic strategies targeting the TME^44, 45^. The stromal score, reflecting the abundance of stromal cells within tumor tissue, is often associated with tumor malignancy^46^. We observed significant correlations between HMGCS2 expression and stromal scores in 13 cancer types. Specifically, LGG, BRCA, KIPAN, and TGCT exhibited significant positive correlations, while LUAD, ESCA, COAD, COADREAD, KIRC, THYM, BLCA, OV, and PAAD displayed significant negative correlations (Fig. 4A). Furthermore, the immune score, which evaluates the tumor immune microenvironment, outperforms the AJCC/UICC TNM classification in colorectal cancer^47, 48^. HMGCS2 expression demonstrated a negative correlation with immune scores in LUAD, COAD, COADREAD, KIRC, LIHC, BLCA, THCA, OV, and TGCT, while showing a positive correlation in LGG and MESO (Fig. 4B). The combination of stromal score and immune score yields an estimate score that reflects tumor purity by considering the presence of stromal and immune cells. Additionally, significant correlations were observed between HMGCS2 expression and immune infiltration in 12 cancer types. Notably, LGG, BRCA, and KIPAN displayed significant positive correlations, while LUAD, COAD, COADREAD, KIRC, LIHC, BLCA, THCA, OV, and PAAD exhibited significant negative correlations (Fig. 4C). Remarkably, our results consistently emphasize the strong association between HMGCS2 and KIRC and LIHC, as supported by gene expression patterns, prognostic prediction, and immune infiltration analysis (Figs. 1, 2, 3, 4).

### HMGCS2 is associated with immune cell infiltration in pan-cancer

**Figure 5:**
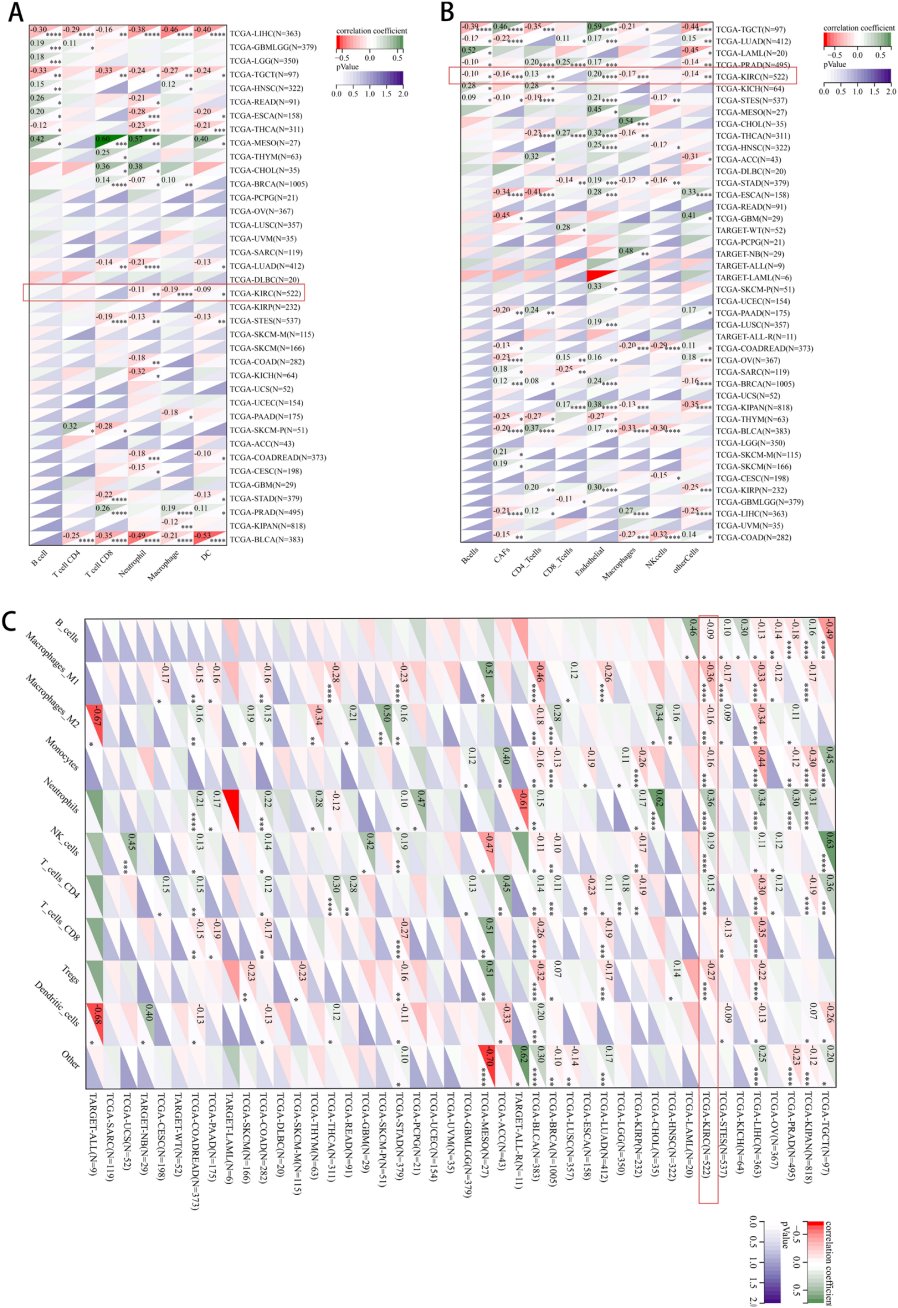
Comprehensive immune cell infiltration analysis of HMGCS2 in various cancers. (**A**,**B**) Association of HMGCS2 expression with various immune infiltrating cells analyzed in timer (**A**), EPIC (**B**) and quanTIseq databases.

To further investigate the correlation between HMGCS2 and immune cell infiltration across different cancer types, we conducted a comprehensive analysis using various databases. Results from the Timer database revealed that HMGCS2 expression was associated with immune cell infiltration in 25 cancers, with a particular focus on LIHC, TGCT, and BLCA (Fig. 5A). The EPIC and quanTIseq databases further supported the association between HMGCS2 and immune cell infiltration in most cancer types. Notably, HMGCS2 displayed significant associations with TGCT and KIRC in EPIC, as well as LIHC, KIRC, and BLCA in quanTIseq (Fig. 5B,C). Previous studies have demonstrated the tumor-suppressive effect of HMGCS2 in hepatocellular carcinoma (HCC)^19, 22^. Thus, we aim to investigate the role of HMGCS2 specifically in KIRC. Notably, our findings suggest that HMGCS2 may influence the immune landscape of KIRC through its impact on macrophages (as indicated by the red box).

To further explore the immune correlates of HMGCS2, we examined 60 immune checkpoint genes and 150 immune pathway genes^25, 30^. The analysis revealed a strong association between HMGCS2 and BLCA, followed by TGCT. In KIRC, 33 out of the 60 immune checkpoint genes were significantly associated with HMGCS2 expression (Supplementary Fig. 2A), and 75 out of the 150 immune pathway marker genes showed significant correlations with HMGCS2 (Supplementary Fig. 2B). Additionally, the discovery of new drugs and the understanding of programmed cell death in cancer are closely linked to RNA modifications, such as m6A modification^49, 50^. Therefore, we performed correlation analysis between HMGCS2 expression and 44 gene expression profiles related to three types of RNA modifications (m1A, m5C, m6A). Remarkably, in KIRC, the most relevant cancer type, 34 out of the 44 genes displayed significant associations with HMGCS2 expression (Supplementary Fig. 2C).

### HMGCS2 expression is closely related to renal proximal tubules

**Figure 6:**
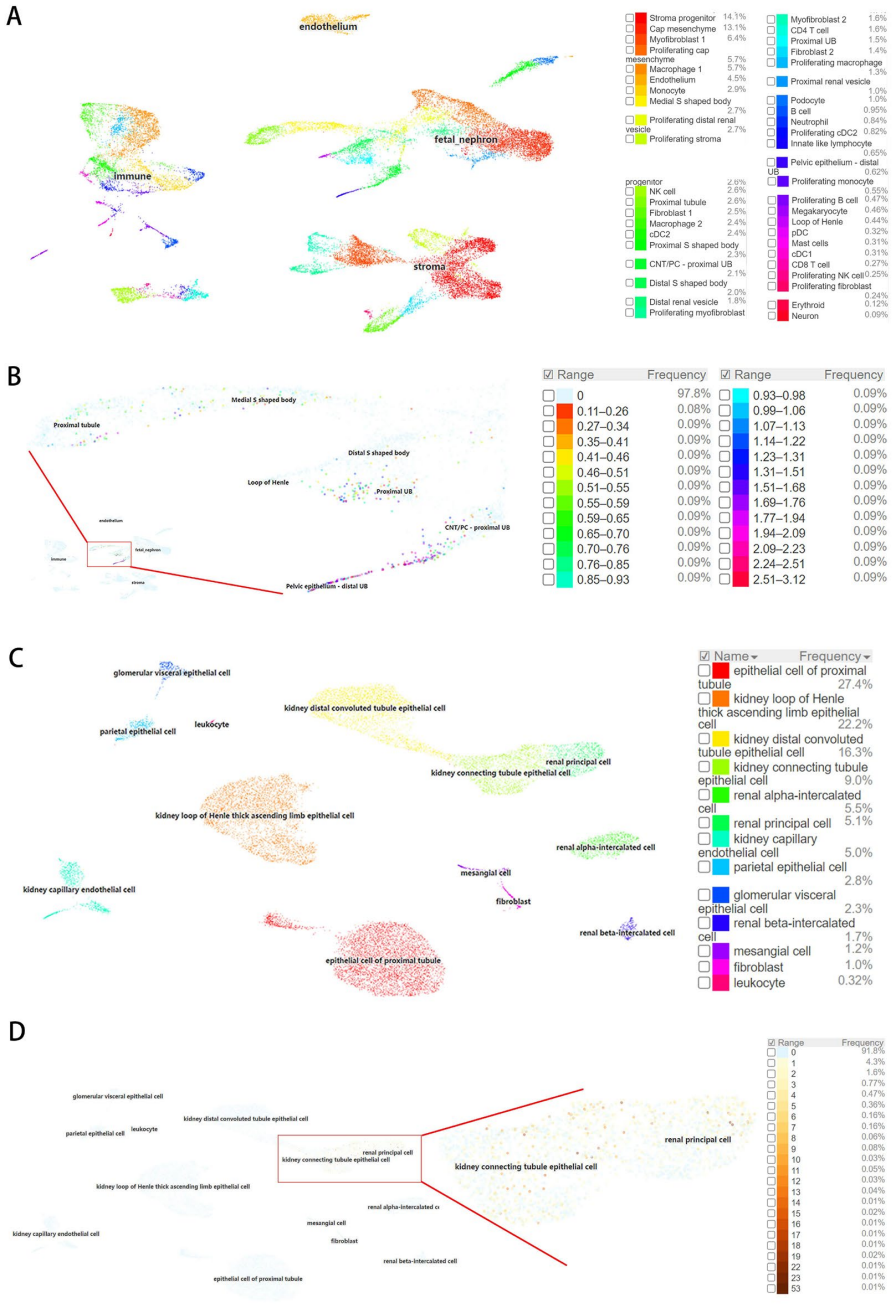
Single-cell analysis of the role of HMGCS2 in kidney development. (**A**) UMAP plot of the GSE151302 dataset. (**B**) UMAP map of cell type assignment of HMGCS2 as a marker gene in the GSE151302 dataset. (**C**) UMAP plot of 40,268 human mature kidney cells. (**D**) Cell type assignment of HMGCS2 as a marker gene.

To gain further insights into the role of HMGCS2 in kidney development, we conducted an analysis of two single-cell sequencing datasets obtained from adult kidneys. The cells were categorized into four major populations, including immune cells, endothelial cells, fetal nephrons, and stromal cells (Fig. 6A). Subsequently, we examined the expression of HMGCS2 as a marker gene, and the results revealed its specific expression in the pelvic epithelium-distal UB, proximal UB, and proximal tubule (Fig. 6B). In the second dataset, the cells were divided into thirteen distinct populations, such as kidney capillary endothelial cells, glomerular visceral epithelial cells, and epithelial cells of the proximal tubule (Fig. 6C). Furthermore, the expression of HMGCS2 exhibited correlation with kidney connecting tubule epithelial cells and renal principal cells (Fig. 6D). These findings provide valuable insights into the specific cell populations in the kidney where HMGCS2 is expressed during development.

### Ectopic expression of HMGCS2 suppresses proliferation and glycolysis in OSRC2 cells

**Figure 7:**
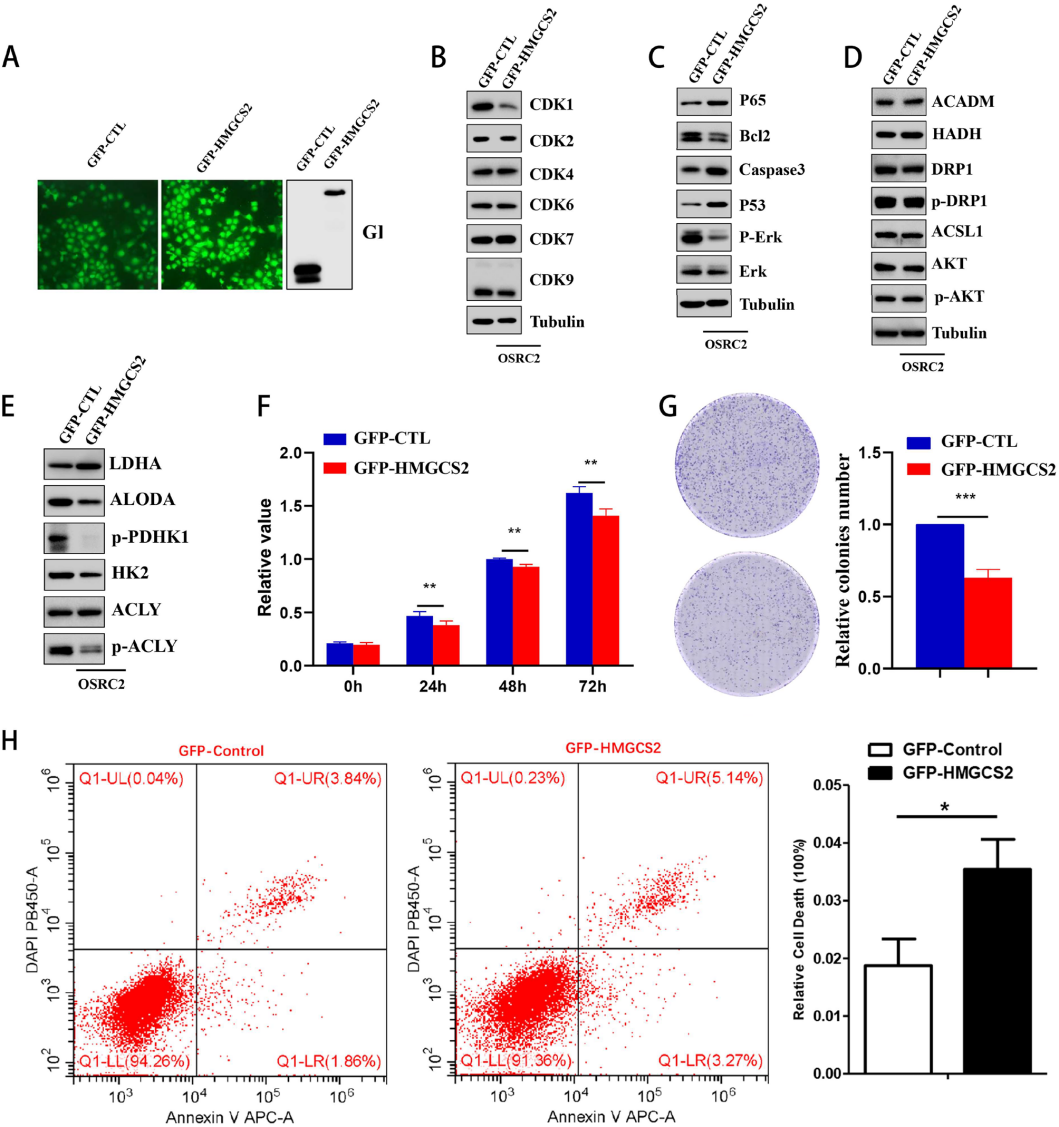
Function of HMGCS2 in OSRC2. (**A**) Western blot and fluorescence detection of GFP-HMGCS2 overexpression in OSRC2. (B-E) Western blot detection of the effect of GFP-HMGCS2 overexpression on the levels of CDK family proteins (**B**), apoptosis-related proteins (**C**), and mitochondrial or metabolism-related proteins (**D**,**E**). (**F**) The results of CCK-8 assay showed that the viability of OSRC2 cells decreased after HMGCS2 overexpression. (**G**) Colony formation assay detected that overexpression of HMGCS2 reduced the number of colonies formed in OSRC2 cells. (**H**) Representative flow cytometry images, the histogram on the right is the statistical analysis of the results.

We performed ectopic expression of GFP-HMGCS2 in the human renal clear cell carcinoma cell line OSRC2 to investigate the biological functions of HMGCS2 in renal clear cell carcinoma (Fig. 7A). Ectopic expression of HMGCS2 suppressed the expression of the cell cycle protein CDK1 but did not affect other members of this family (Fig. 7B). Additionally, ectopic expression of HMGCS2 increased the levels of p65, caspase3, and p53 while decreasing the levels of Bcl2 and Erk in OSRC2 cells (Fig. 7C). HMGCS2 is a key metabolic enzyme in mitochondria (31442404), therefore it is necessary to examine the metabolic changes induced by ectopic expression of HMGCS2. By evaluating the protein levels of metabolic key enzymes, we found that ectopic expression of HMGCS2 might affect the glucose metabolism pathway rather than the lipid metabolism pathway in OSRC2 cells (Fig. 7D,E). Importantly, ectopic expression of HMGCS2 did not alter key proteins closely related to mitochondrial function, such as ACADM, DRP1, and p-DRP1 (Fig. 7D). Furthermore, we explored the biological functions of HMGCS2 using cell viability assays, colony formation assays, and flow cytometry. The results indicated that ectopic expression of HMGCS2 inhibited proliferation (Fig. 7F,G) and promoted apoptosis in OSRC2 cells (Fig. 7H). These findings suggest that HMGCS2 may act as a tumor suppressor by inhibiting cell cycle progression and promoting apoptosis ADDIN EN.CITE^51^.

### Knockdown of HMGCS2 enhances proliferation in renal clear cell carcinoma

**Figure 8:**
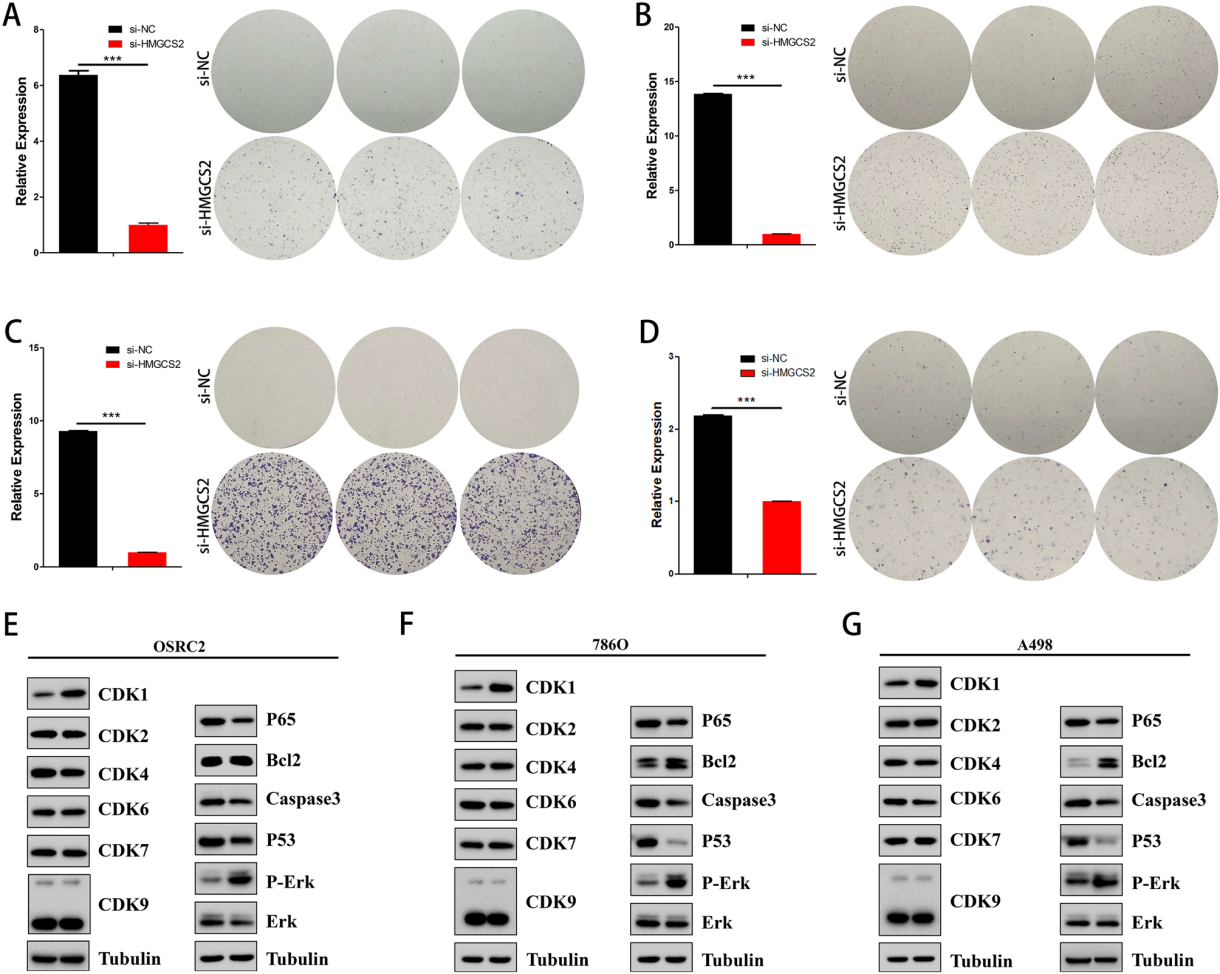
The effect of HMGCS2 knockdown on renal clear cell carcinoma cells and normal human renal proximal tubular cells. (**A**–**D**) HMGCS2 was knocked down by si-RNA in OSRC2 (**A**), 786O (**B**), A498 (**C**) and HK2 (**D**), and their proliferative ability was detected by clonogenic assay. The bar graph on the left shows the knockdown efficiency of si-HMGCS2. (**E**–**G**) Western blot detection of the effect of si-HMGCS2 on CDK family proteins and apoptosis-related proteins in OSRC2 (**E**), 786O (**F**) and A498 (**G**).

Given that HMGCS2 shows a downregulation trend in many cancer types, it is important to further investigate the consequences of HMGCS2 downregulation in renal clear cell carcinoma and normal human kidney cells. Following the study by Wang et al. (32457595), we performed HMGCS2 knockdown in renal clear cell carcinoma cell lines (OSRC2 (Fig. 8A), 786-O (Fig. 8B), A498 (Fig. 8C)), as well as in normal human proximal tubule cells (Fig. 8D). Evidently, the use of si-HMGCS2 promoted their proliferation (Fig. 8A–D), particularly in A498 cells (Fig. 8C). Furthermore, changes in cell cycle and apoptosis-related proteins were detected in the three renal clear cell carcinoma cell lines (Fig. 8E–G), consistent with the previous findings (Fig. 7B,C).

## Discussion

HMGCS2 was previously reported as a key rate-limiting enzyme for ketone body production, involved in mitochondrial maturation and metabolic reprogramming^51, 52^. HMGCS2 has been previously reported as a key rate-limiting enzyme involved in ketone body production and is implicated in mitochondrial maturation and metabolic reprogramming^52, 53^. Furthermore, HMGCS2-deficient mice display hyperglycemia and impaired gluconeogenesis^54^. However, there have been limited systematic studies evaluating the role of HMGCS2 through pan-cancer analysis using bioinformatics approaches. This study aims to systematically determine the expression patterns, prognostic value, and potential functions of HMGCS2 in different types of cancer.

In this study, HMGCS2 was found to be downregulated in almost all cancer tissues, suggesting its potential role as a suppressor. Consistent with previous research, HMGCS2 was primarily localized in the mitochondria^2, 55, 56^. and may be upregulated in certain cancer cell lines, such as RT4 (bladder cancer) and OE19 (esophageal cancer). Subsequent survival analysis revealed correlations between HMGCS2 and clinical parameters in various cancers, particularly in LIHC and KIRC, which were further supported by Kaplan–Meier survival analysis. Previous studies have also indicated that low expression of HMGCS2 is an adverse prognostic factor in hepatocellular carcinoma (HCC)^25, 57–59^, but there have been no specific reports on renal clear cell carcinoma to date. On the other hand, studying tumor stemness and genomic heterogeneity can enhance our understanding of negative aspects of tumorigenesis, metastasis, and drug resistance^60–63^. In this study, HMGCS2 exhibited associations with stemness and heterogeneity in multiple cancers, particularly in KIRC and LIHC.

Renal cell carcinoma (RCC) is considered one of the most immune-infiltrated tumors^64, 65^, and emerging evidence suggests the involvement of specific metabolic pathways in regulating angiogenesis and inflammatory features^66, 67^. Therefore, understanding the dynamic tumor microenvironment (TME) is crucial for enhancing the response of clear cell renal cell carcinoma (ccRCC) patients to immune-targeted therapies. The transcription levels of HMGCS2 showed correlations with stromal scores in 13 cancers, immune scores in 11 cancers, and estimate scores in 12 cancers. Notably, a comprehensive analysis of three immune cell infiltration assessments revealed a stronger association of HMGCS2 with LIHC, TGCT, BLCA, and KIRC (ranked by the number of associated immune cells). Specifically, HMGCS2 appears to modulate the immune status of KIRC through macrophage alterations (highlighted by the red box), indicating its potential role in macrophage polarization. Additionally, HMGCS2 expression positively correlated with immune inhibitory genes such as CD276, IL4, and TGFB1. Tumor-associated macrophages (TAMs) interact with cancer cells and stromal cells in the tumor microenvironment, contributing to the maintenance of various cancer traits and displaying strong clinical relevance^68^. CD276 has been shown to suppress anti-tumor immunity through the CCL2-CCR2-M2 macrophage axis^69^, further supporting the key role of HMGCS2 in regulating tumor immunity and macrophage polarization.

To our knowledge, this is the first comprehensive pan-cancer analysis focusing on the significance of HMGCS2 in tumors. The rapid and cost-effective nature of next-generation sequencing allows individual cancer patients to take proactive therapeutic approaches^70^. Pan-cancer analyses have been employed to uncover common features and/or heterogeneity in key biological processes that contribute to dysregulated tumor microenvironments^71, 72^. Therefore, identifying the differential expression and roles of HMGCS2 across various tumors through pan-cancer analysis holds clinical significance. In our pan-cancer analysis, the strong correlations of HMGCS2 with KIRC and LIHC prompted us to further validate its functions through cell biology experiments. Given the established inhibitory role of HMGCS2 in LIHC, we focused on exploring its role in KIRC. Various assays conducted in different renal clear cell carcinoma cell lines consistently demonstrated that loss or ectopic expression of HMGCS2 alters proliferation, cell cycle, and apoptosis in renal clear cell carcinoma, exerting tumor-suppressive effects while inhibiting glycolysis in OSRC2 cells. These findings suggest that HMGCS2 may function as a tumor suppressor by inhibiting the glycolytic process, providing a prospective experimental basis for further investigation of HMGCS2 as a tumor suppressor in clear cell renal cell carcinoma.

Despite our comprehensive pan-cancer analysis providing insights into the tumor suppressor role of HMGCS2, there are significant limitations. Firstly, the study relied on bioinformatics and public databases, which may be incomplete. For example, the TCGA database mainly includes data from Caucasians, with limited representation of other racial groups. Secondly, the specific mechanism underlying the underexpression of HMGCS2 in various tumors remains unclear. Further investigations should focus on unraveling the tumor suppressor mechanism of HMGCS2 and validating its correlation with the tumor immune microenvironment. Lastly, our functional experiments in KIRC were limited and further in vitro and in vivo experiments, including animal models, are necessary to validate our findings.

## Conclusion

Aberrant expression of HMGCS2 in pan-cancer is closely associated with clinical pathology and tumor tissue, suggesting its potential as a therapeutic target for cancer suppression, particularly in clear cell renal cell carcinoma.

### Supplementary Information

Supplementary Legends.

Supplementary Figures.

Supplementary Figure 1.

Supplementary Figure 2.

## Data Availability

Data used in this study can be downloaded from TCGA (https://tcga-data.nci.nih.gov/tcga/, accessed on October 8, 2022), Ucsc Xena (https://xenabrowser.net/datapages/, accessed on October 8, 2022), and CellMiner (https://discover.nci.nih.gov/cellminer/home.do, accessed on October 8, 2022).

## Abbreviations

ACC: Adrenocortical carcinoma
BLCA: Bladder urothelial carcinoma
BRCA: Breast invasive carcinoma
CESC: Cervical squamous cell carcinoma and endocervical adenocarcinoma
CHOL: Cholangiocarcinoma
COAD: Colon adenocarcinoma
COADREAD: Colon adenocarcinoma/Rectum adenocarcinoma esophageal carcinoma
DLBC: Lymphoid neoplasm diffuse large B-cell lymphoma
ESCA: Esophageal carcinoma
FPPP: FFPE pilot phase II
GBM: Glioblastoma
HNSC: Head and neck squamous cell carcinoma
KICH: Kidney chromophobe
KIPAN: Pan-kidney cohort (KICH + KIRC + KIRP)
KIRC: Kidney renal clear cell carcinoma
KIRP: Kidney renal papillary cell carcinoma
LAML: Acute myeloid leukemia
LGG: Low grade glioma
LIHC: Liver hepatocellular carcinoma
LUAD: Lung adenocarcinoma
LUSC: Lung squamous cell carcinoma
MESO: Mesothelioma
OV: Ovarian serous cystadenocarcinoma
PAAD: Pancreatic adenocarcinoma
PCPG: Pheochromocytoma and paraganglioma
PRAD: Prostate adenocarcinoma
READ: Rectum adenocarcinoma
SARC: Sarcoma
STAD: Stomach adenocarcinoma
SKCM: Skin cutaneous melanoma
STES: Stomach and esophageal carcinoma
TGCT: Testicular germ cell tumors
THCA: Thyroid carcinoma
THYM: Thymoma
UCEC: Uterine corpus endometrial carcinoma
UCS: Uterine carcinosarcoma
UVM: Uveal melanoma
OS: Osteosarcoma
ALL: Acute lymphoblastic leukemia
NB: Neuroblastoma
WT: High-risk wilms tumor
TCGA: The Cancer Genome Atlas
GEO: Gene Expression Omnibus
OS: Overall survival
PFI: Progression free interval
