# Supplementary material for: HMGCS2 serves as a potential biomarker for inhibition of renal clear cell carcinoma growth
Source: Sci Rep. 2023 Sep 5;13:14629. doi: 10.1038/s41598-023-41343-7 (PMC10480187; doi:10.1038/s41598-023-41343-7)
Supplement: Supplementary file 1 — Supplementary Legends. [file 41598_2023_41343_MOESM1_ESM.docx]

**Supplementary Figure 1.** HMGCS2 expression correlated with clinical features. (A-D) Correlation of HMGCS2 expression with clinical parameters of various cancers (primary tumor status (T), lymph node metastasis (N), distant metastasis (M), tumor grade (G) and tumor stage (Stage)).

**Supplementary Figure 2.** Correlation analysis of HMGCS2 with genes related to immune checkpoints, immune regulation and RNA modification in various cancers. (A) Association of HMGCS2 with immune checkpoint-related genes in various cancers. (B) Association of HMGCS2 with immune regulation-related genes in various cancers. (C) Association of HMGCS2 with immune modification-related genes in various cancers.
