# Supplementary material for: HMGCS2 serves as a potential biomarker for inhibition of renal clear cell carcinoma growth
Source: Sci Rep. 2023 Sep 5;13:14629. doi: 10.1038/s41598-023-41343-7 (PMC10480187; doi:10.1038/s41598-023-41343-7)

Figure 7B(CDK1)

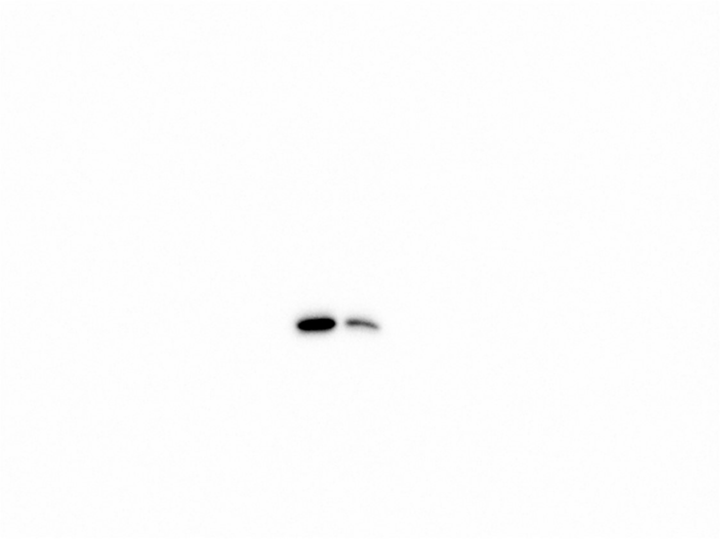

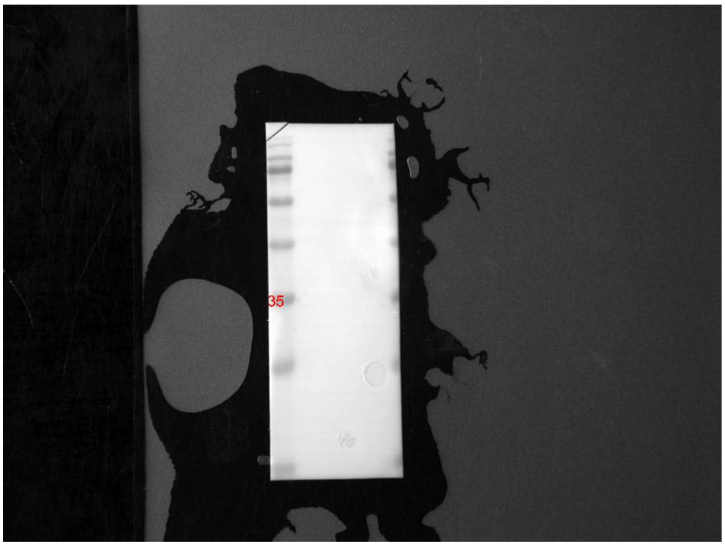

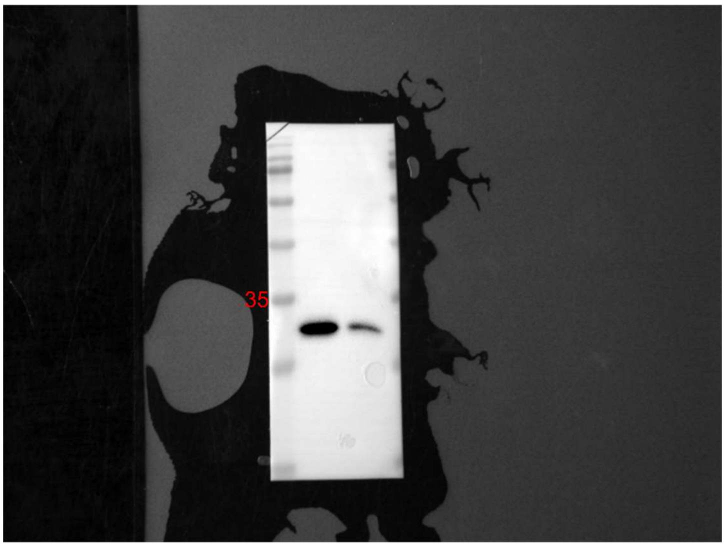

Figure 7B(CDK2)

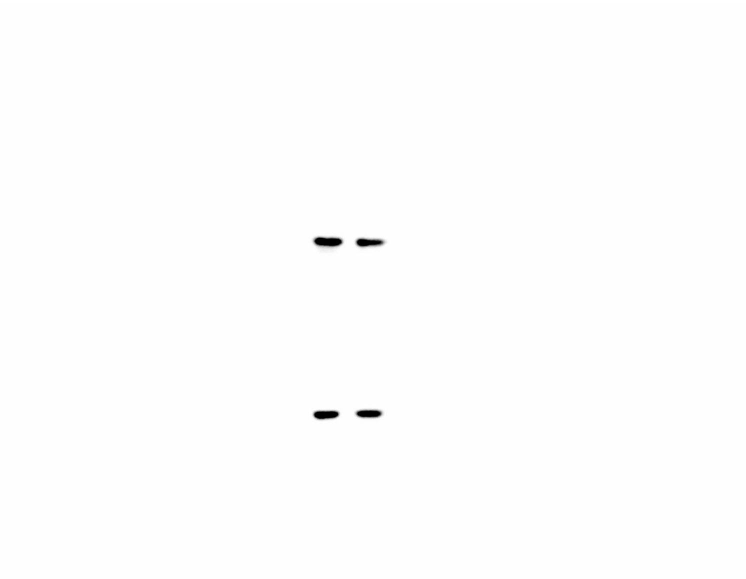

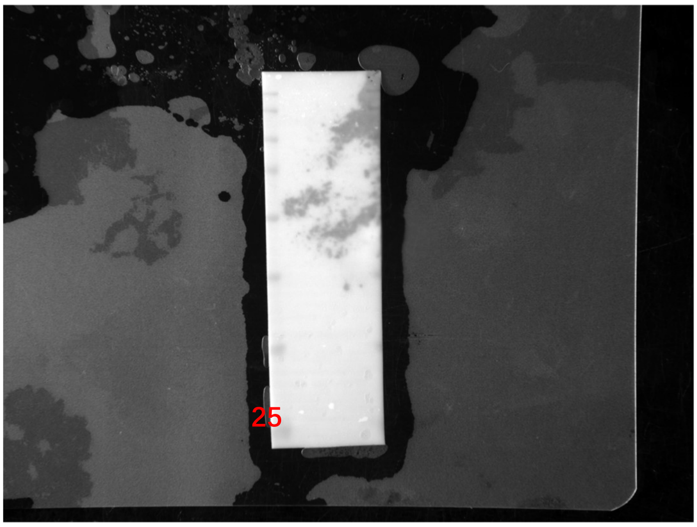

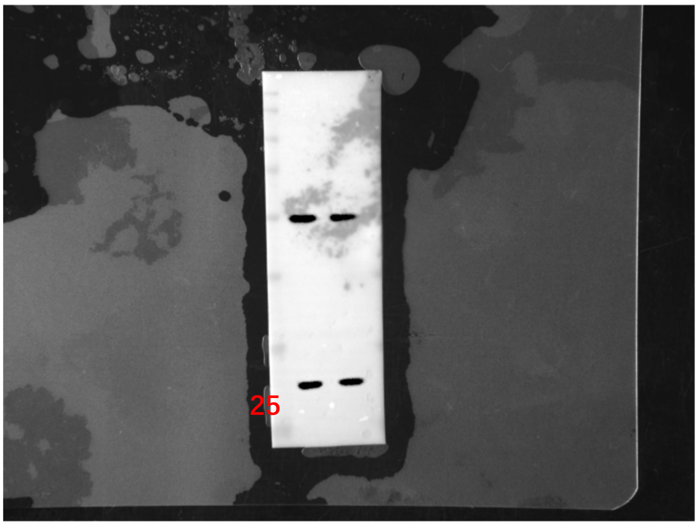

Figure 7B(CDK4)

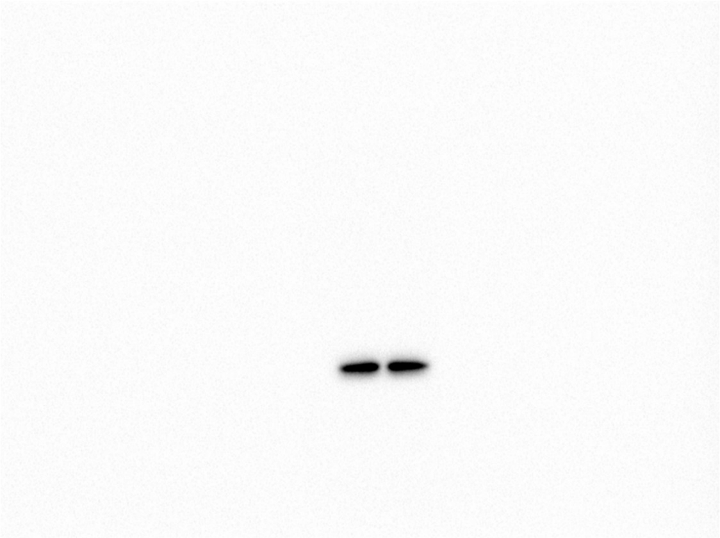

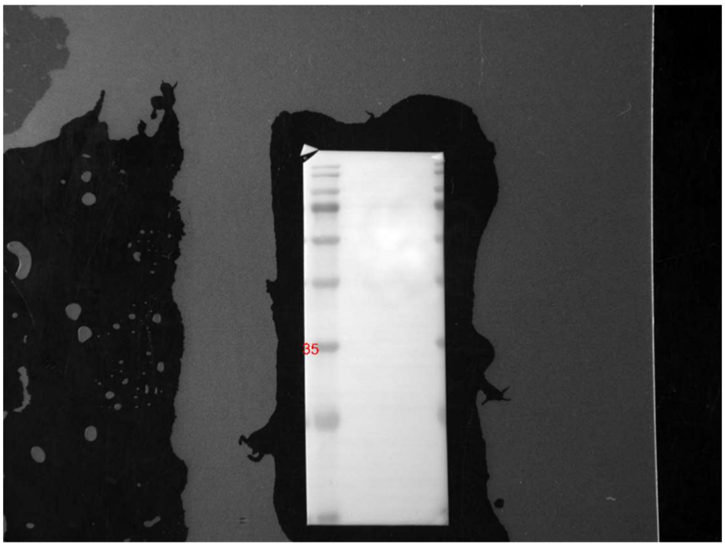

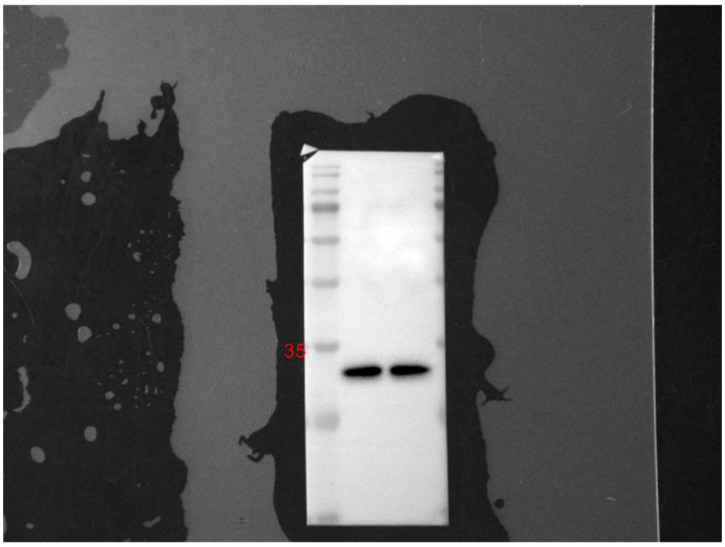

Figure 7B(CDK6)

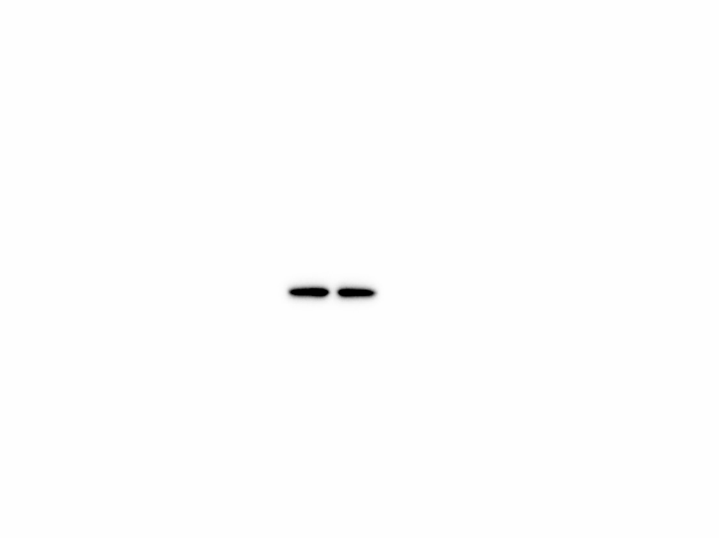

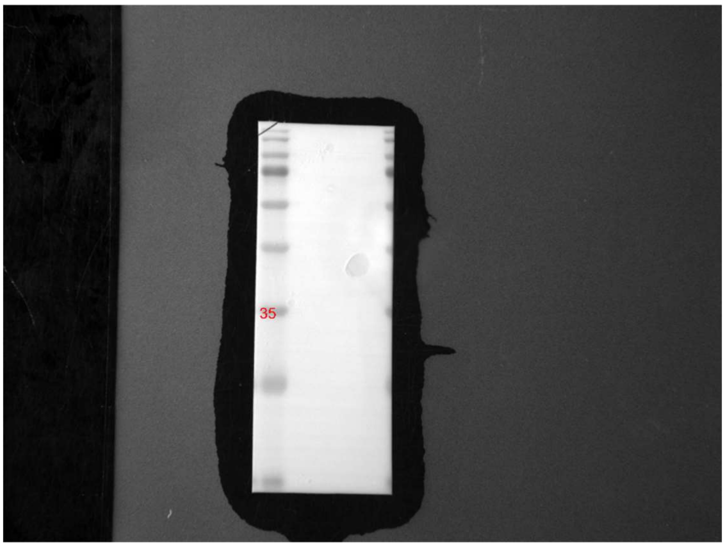

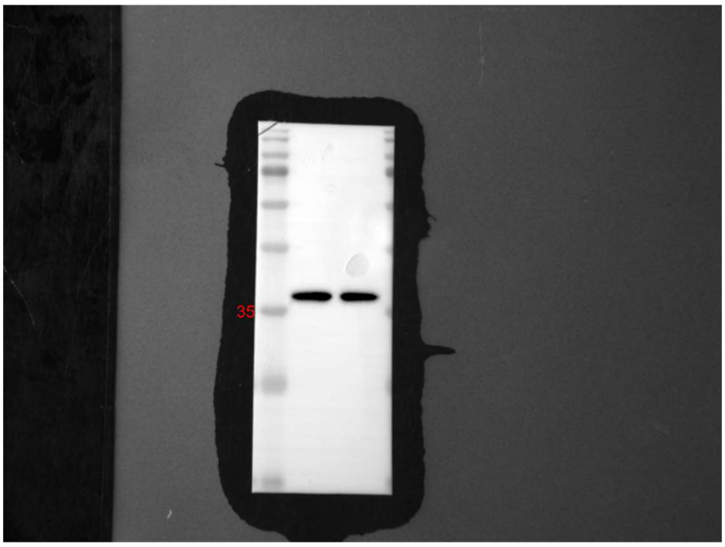

Figure 7B(CDK7)

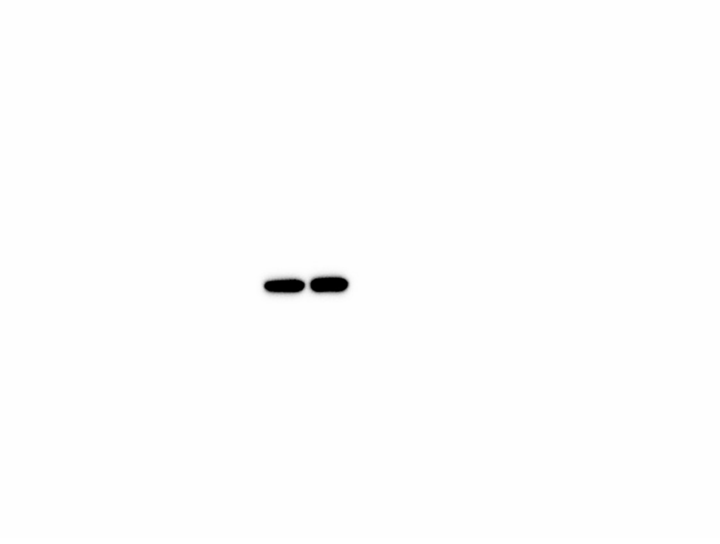

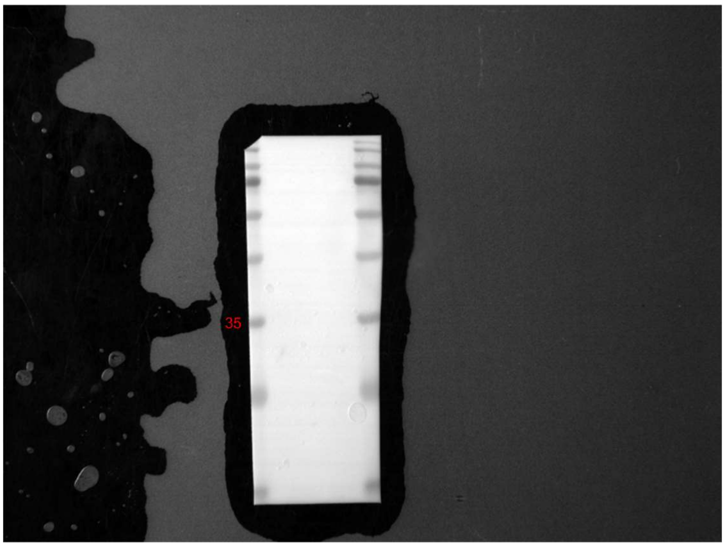

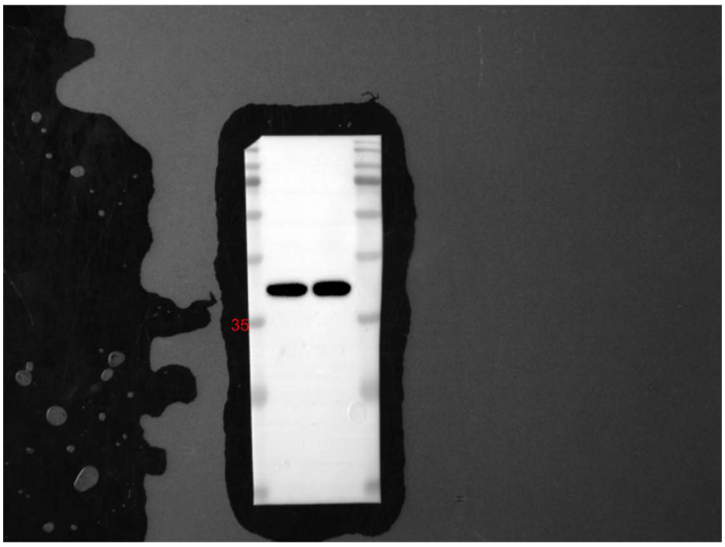

Figure 7B(CDK9)

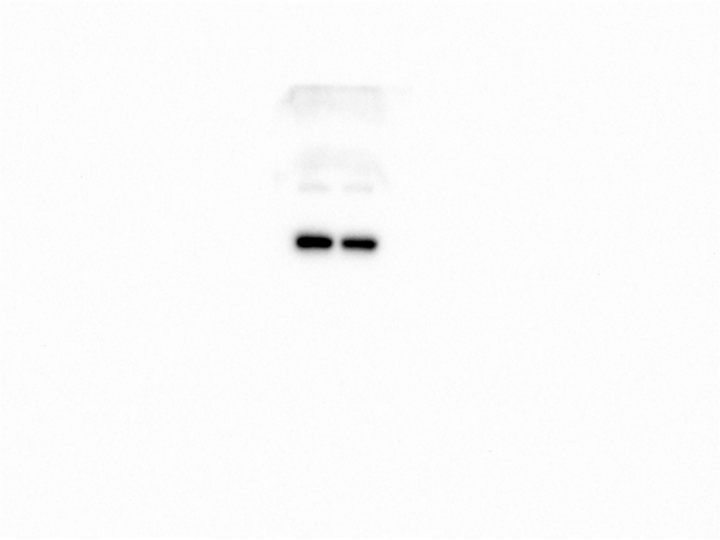

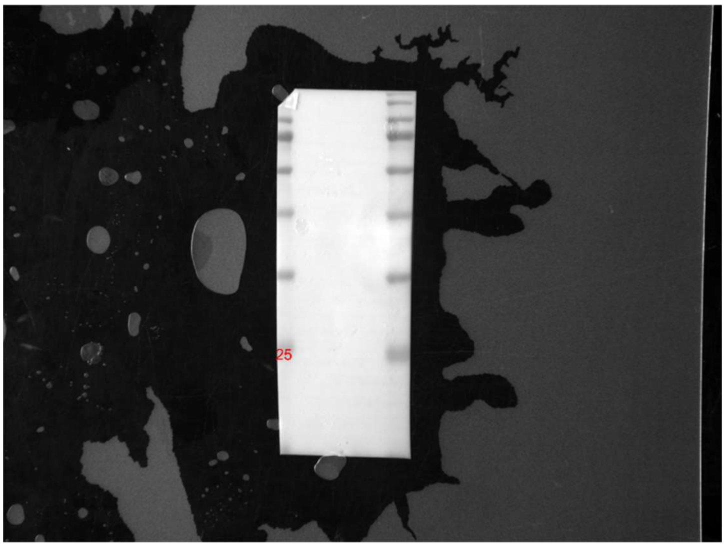

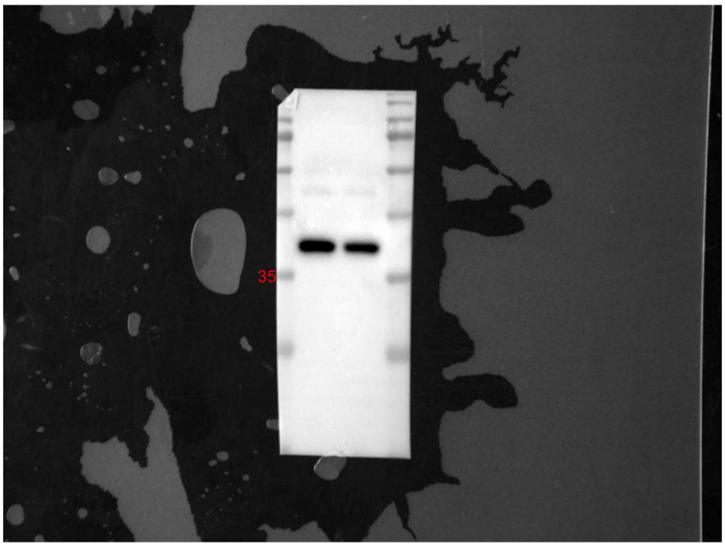

Figure 7B(Tubulin)

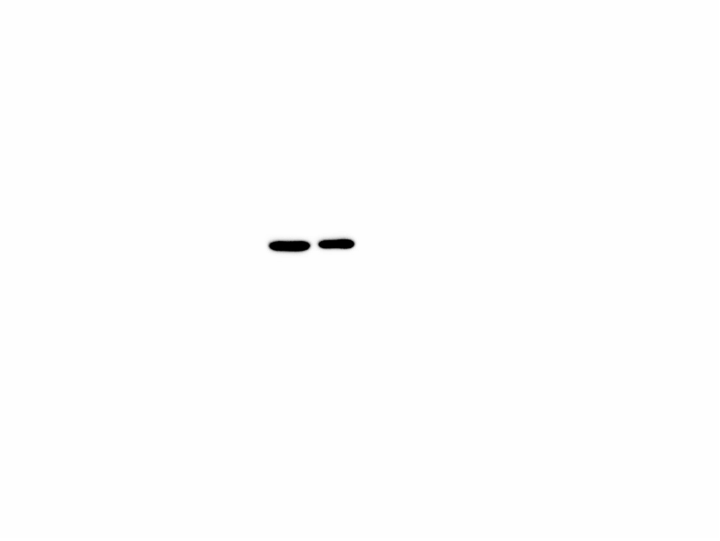

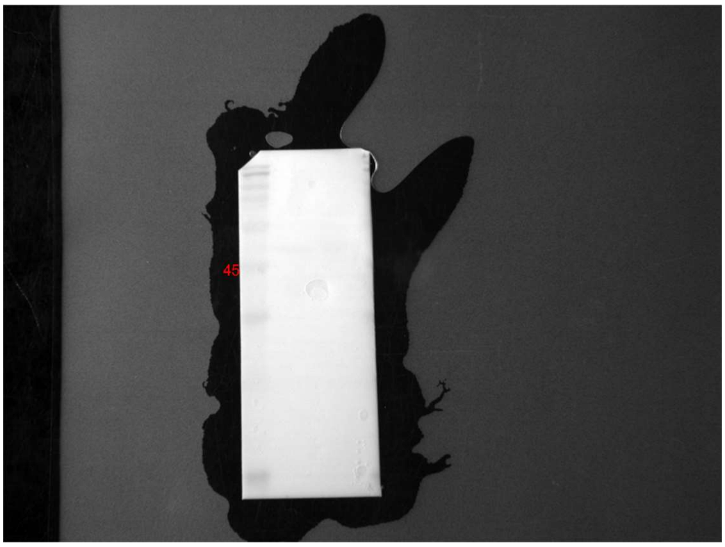

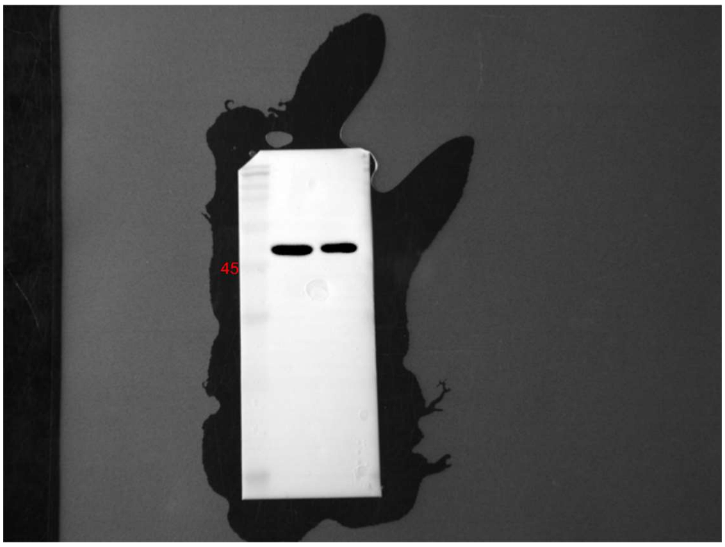

Figure 7C(P65)

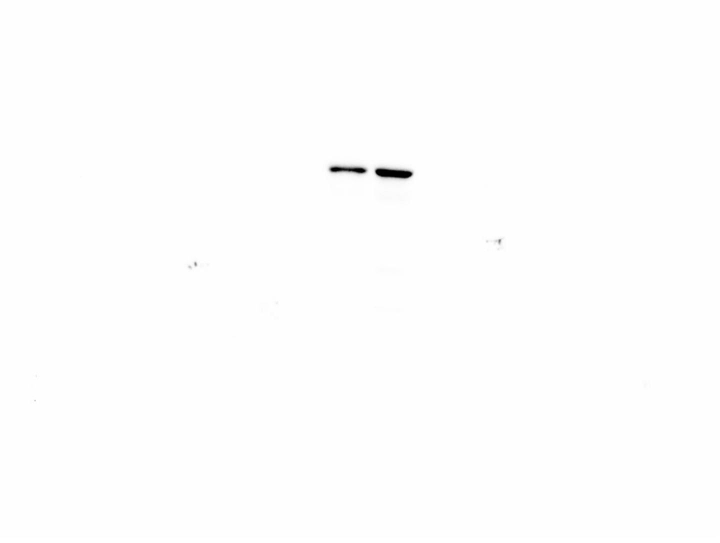

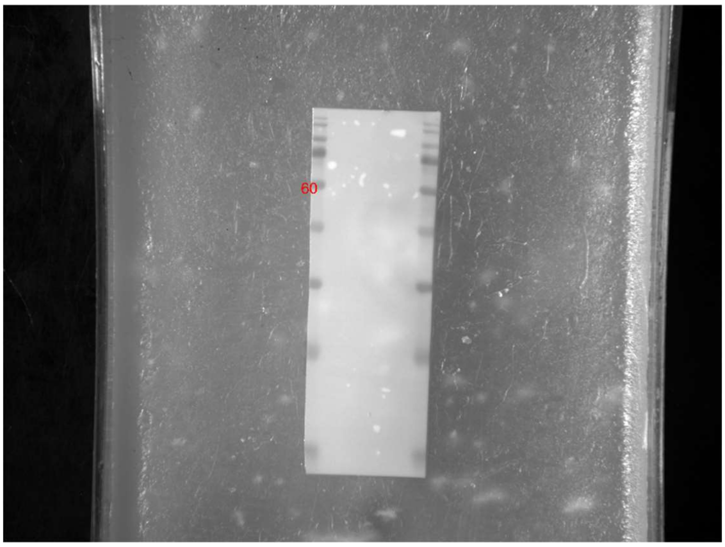

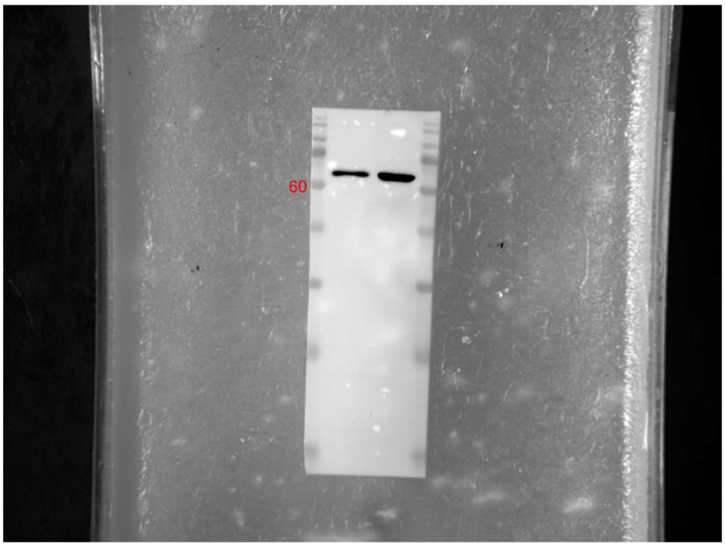

Figure 7C(Bcl2)

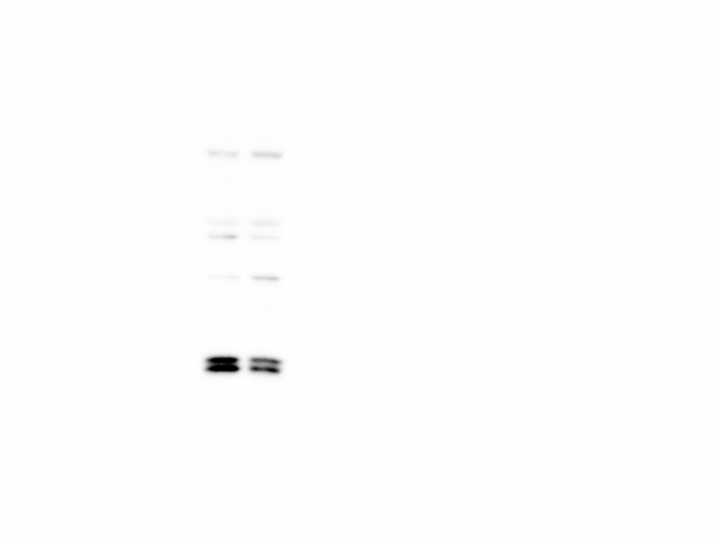

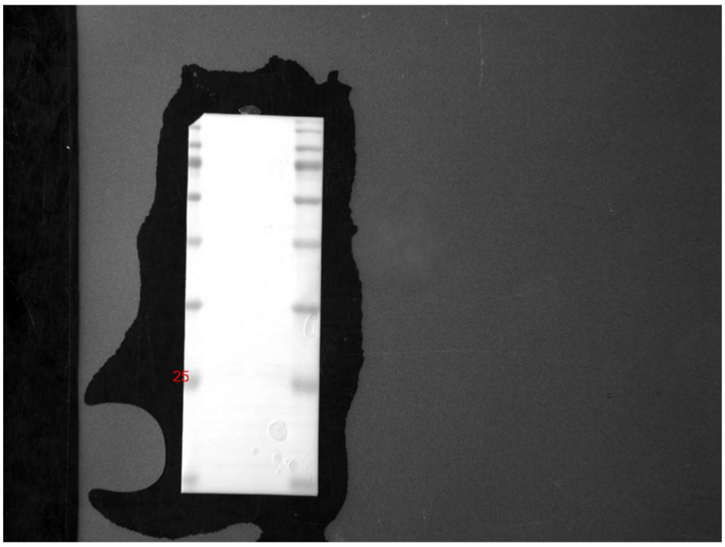

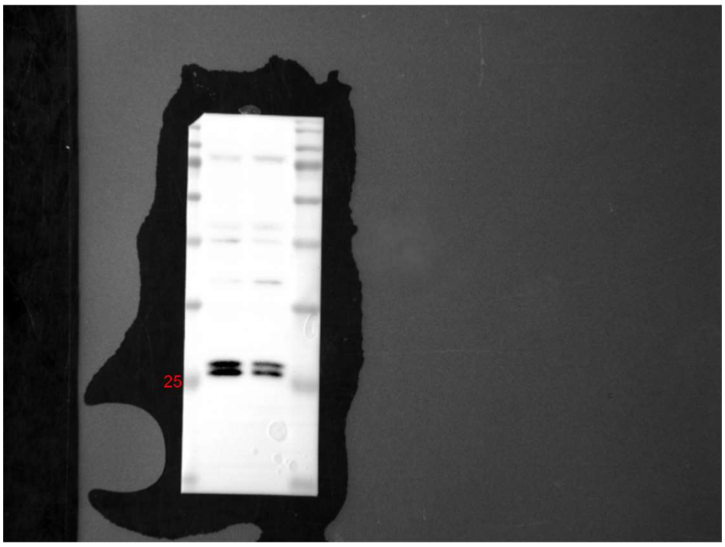

Figure 7C(Caspase3)

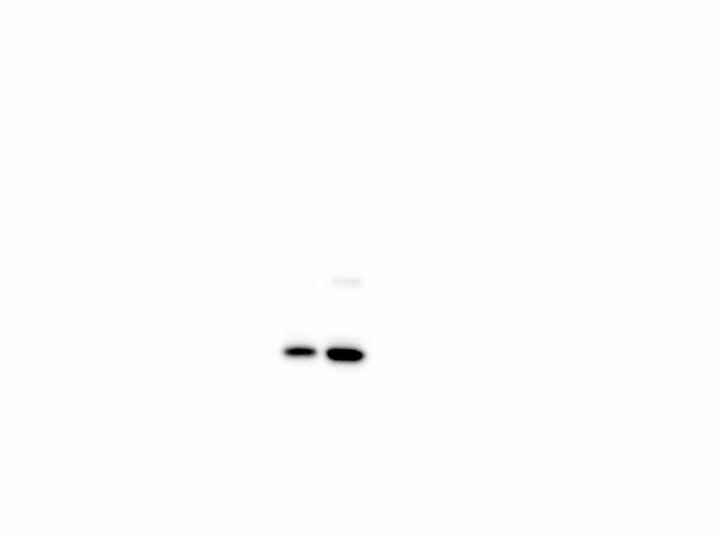

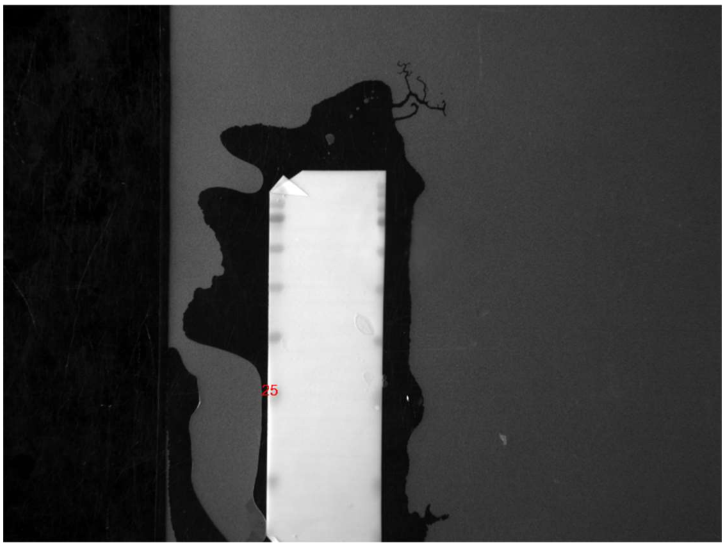

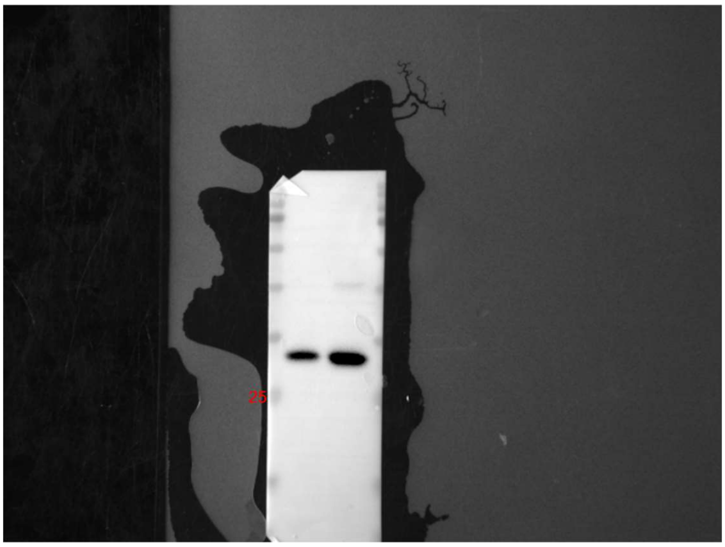

Figure 7C(P53)

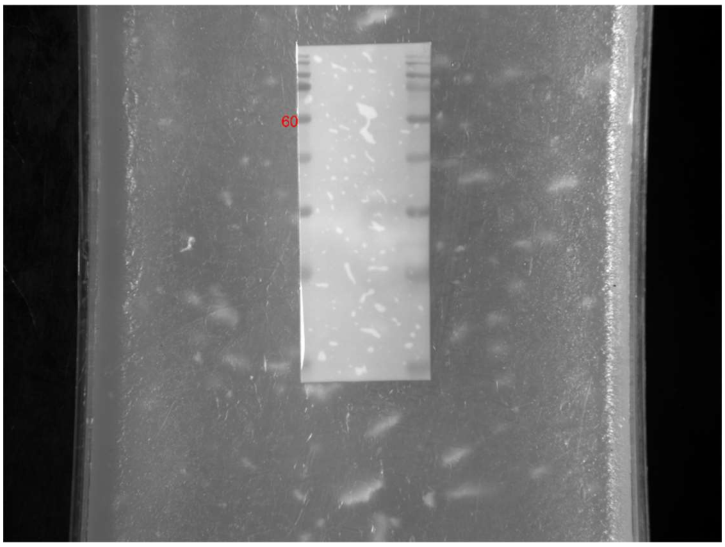

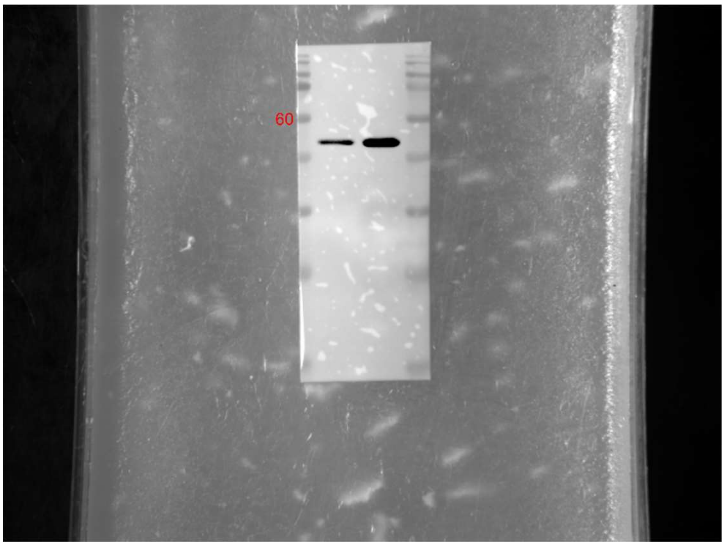

Figure 7C(P-erk)

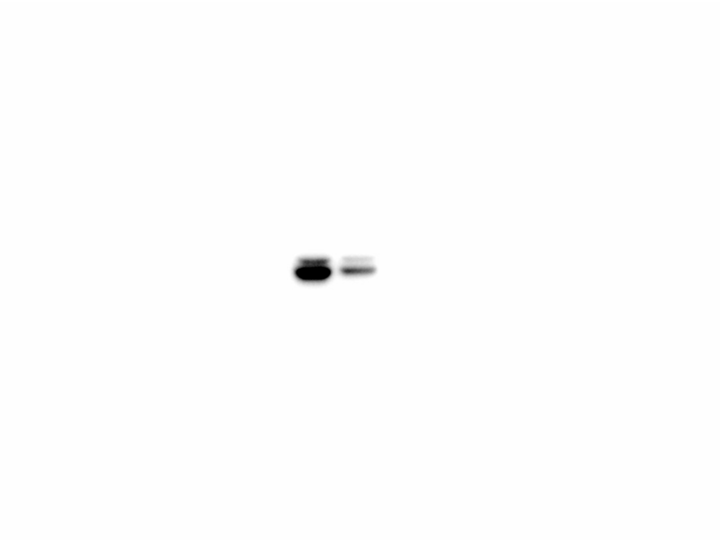

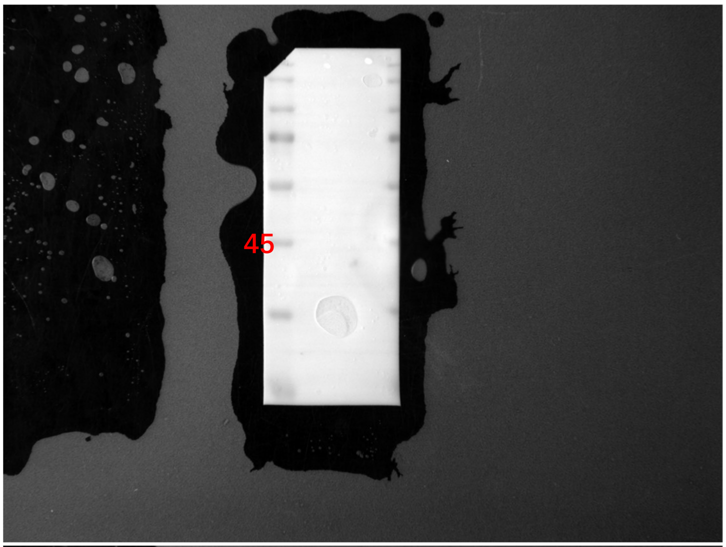

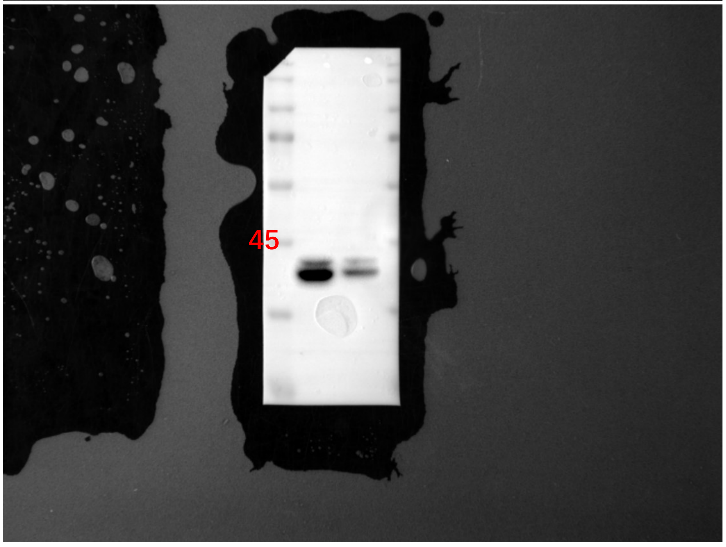

Figure 7C(Erk)

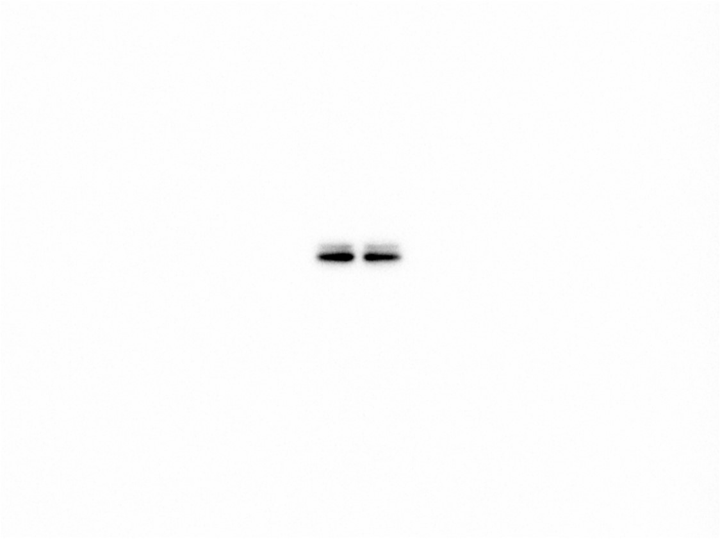

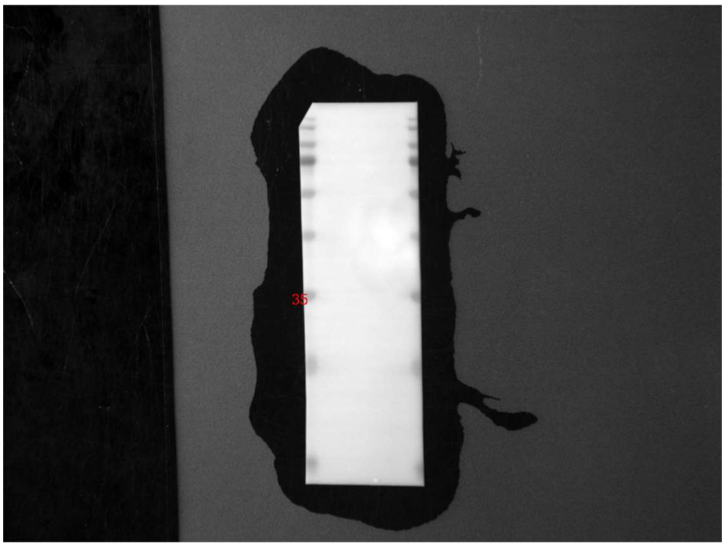

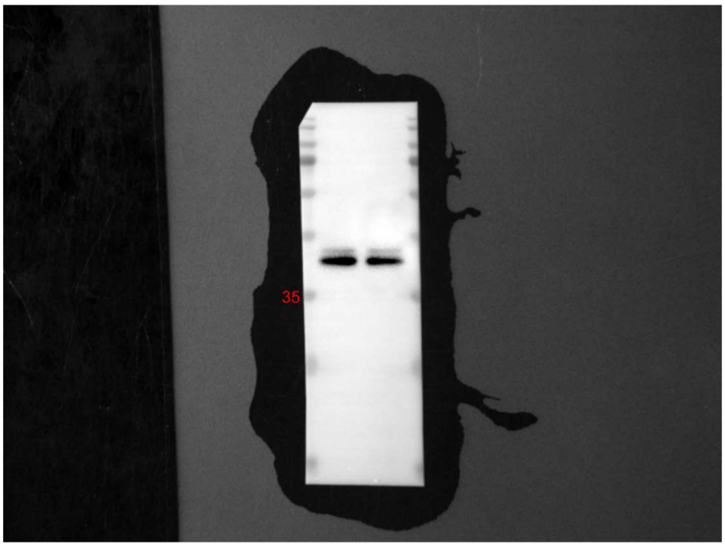

Figure 7C(Tubulin)

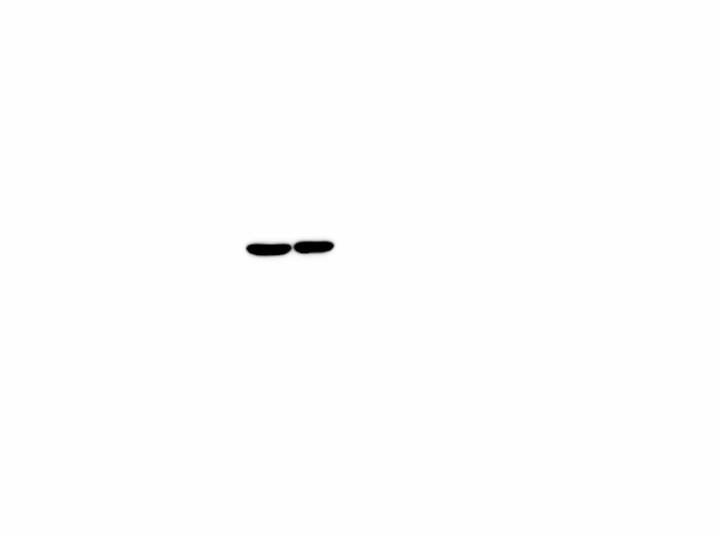

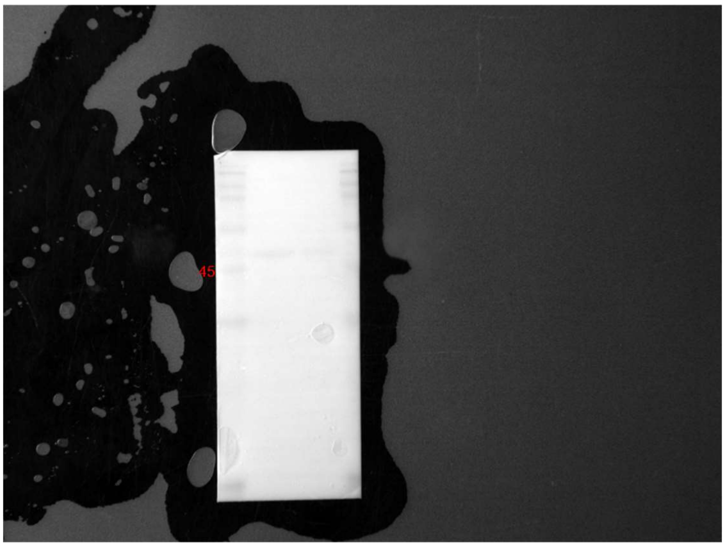

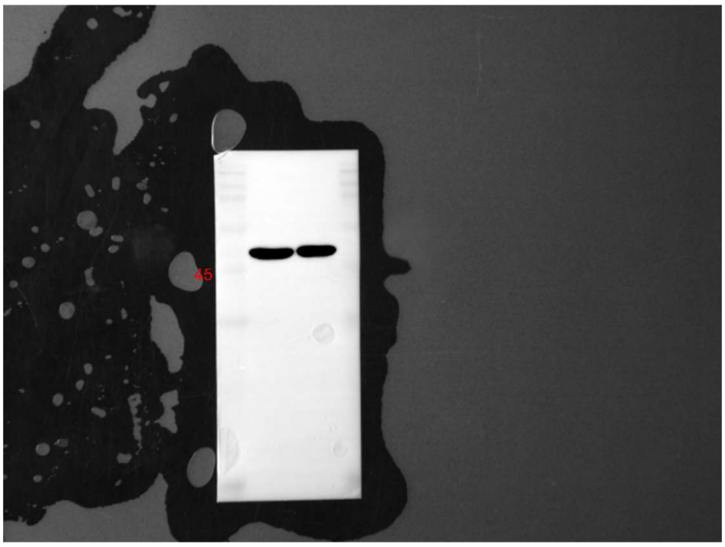

Figure 7D(ACADM)

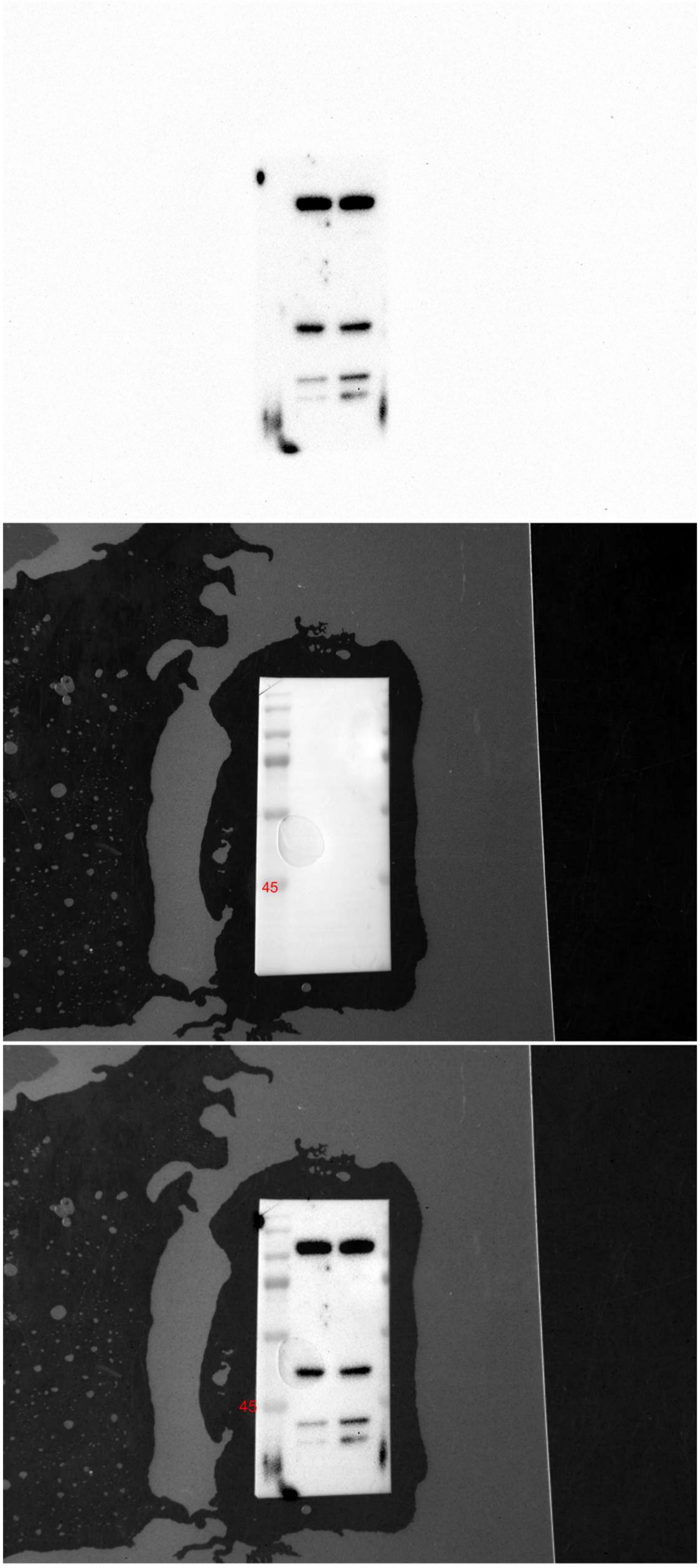

Figure 7D(HADH)

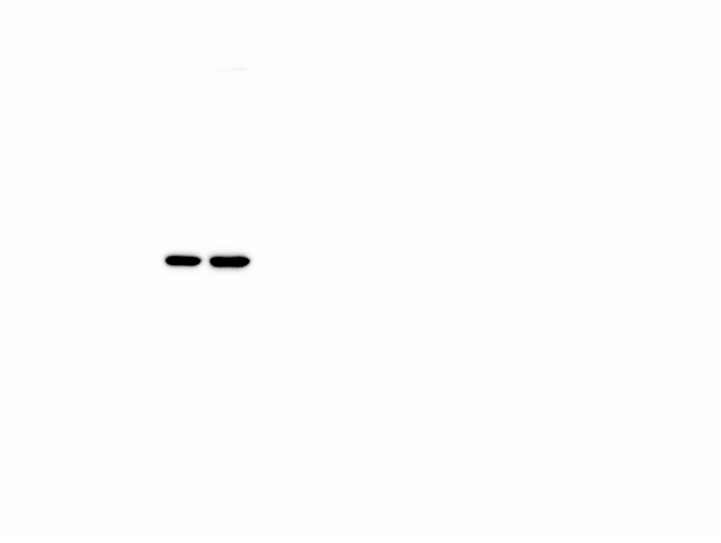

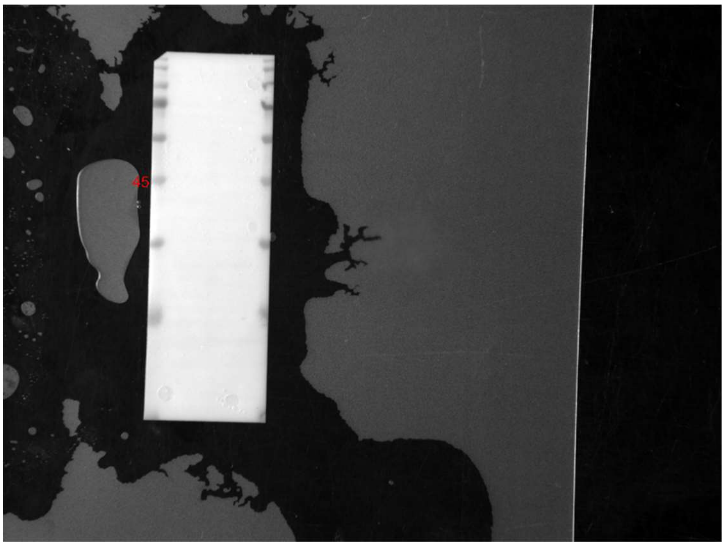

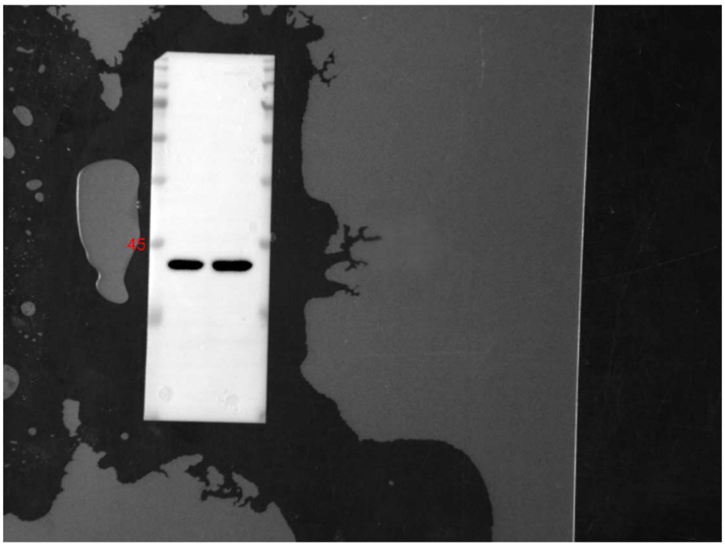

Figure 7D(DRP1)

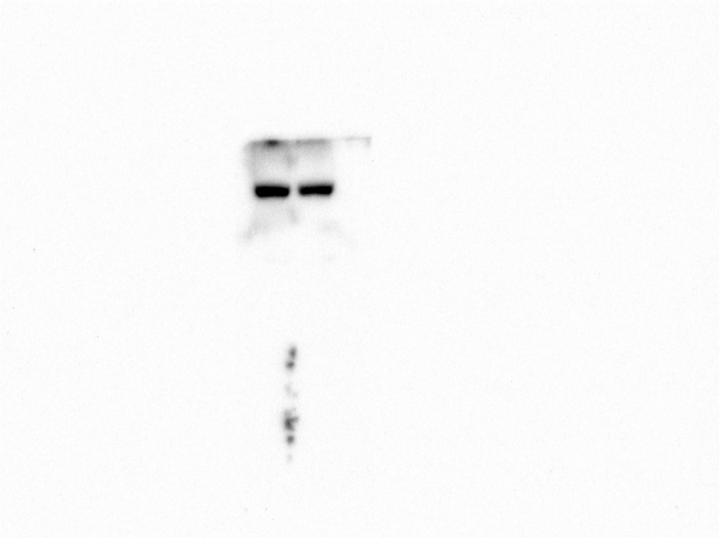

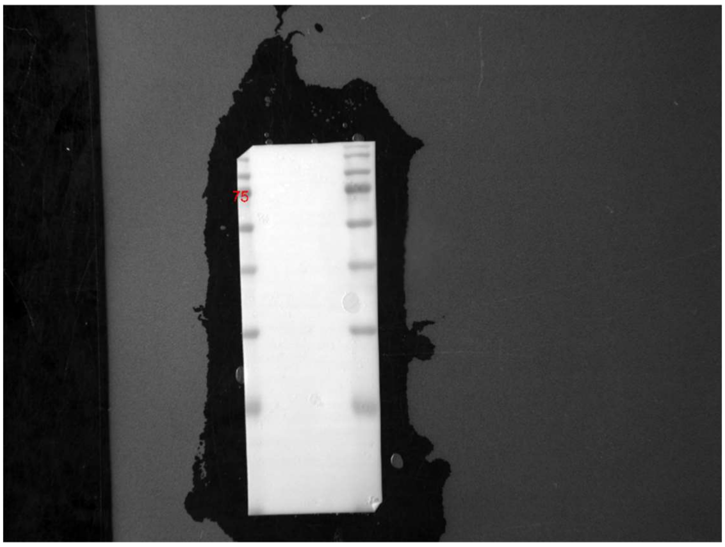

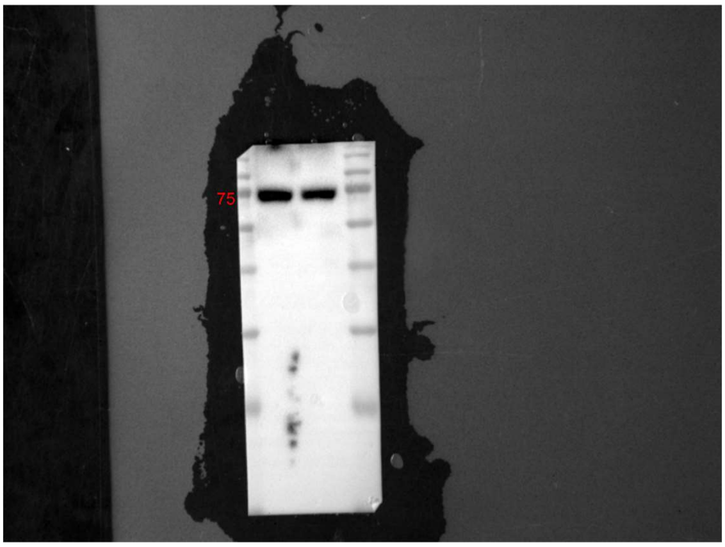

Figure 7D(p-DRP1)

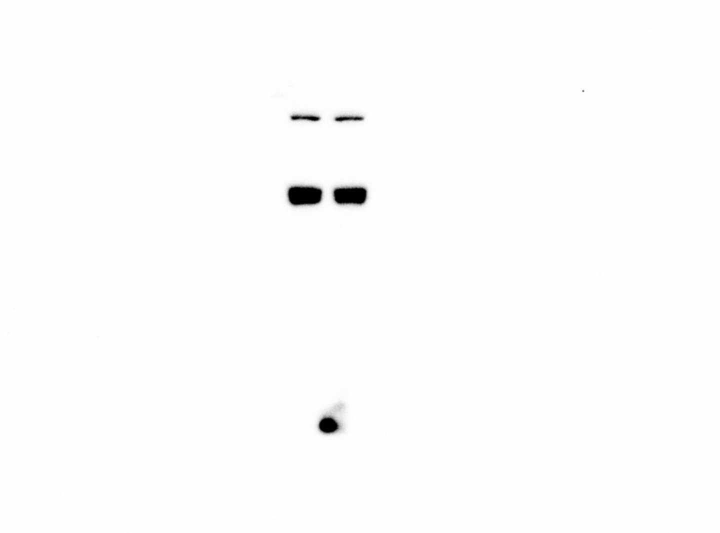

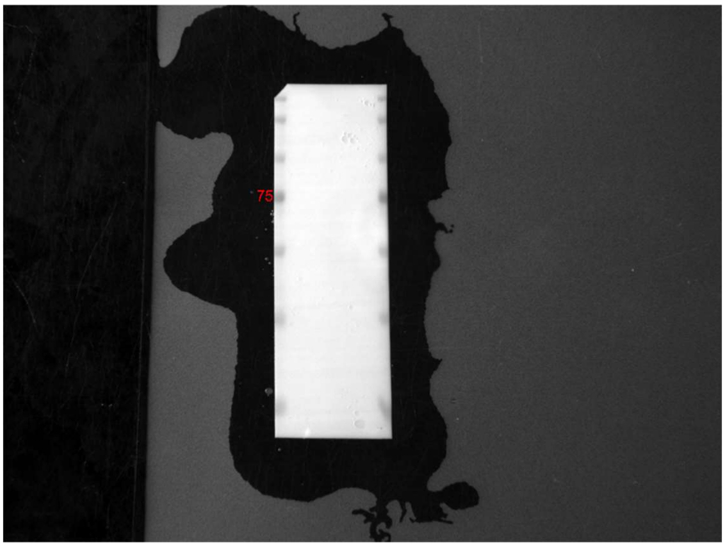

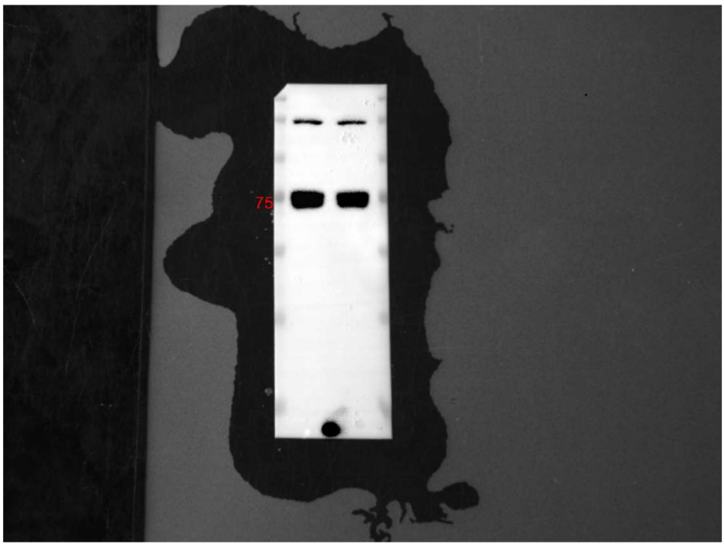

Figure 7D(ACSL1)

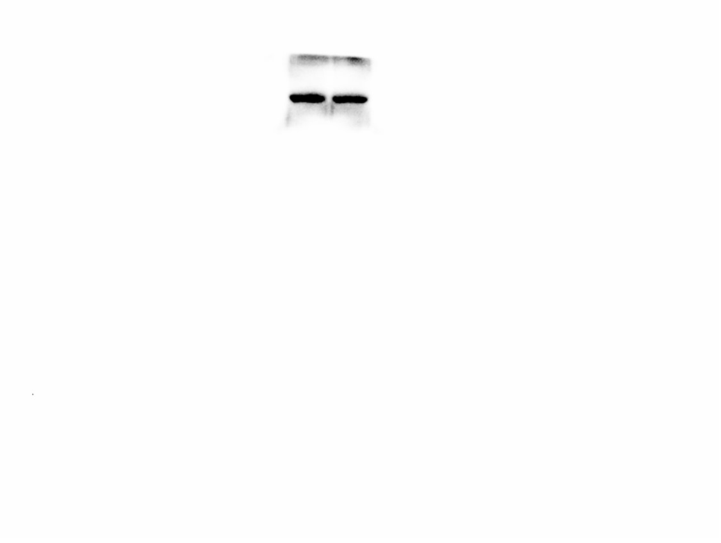

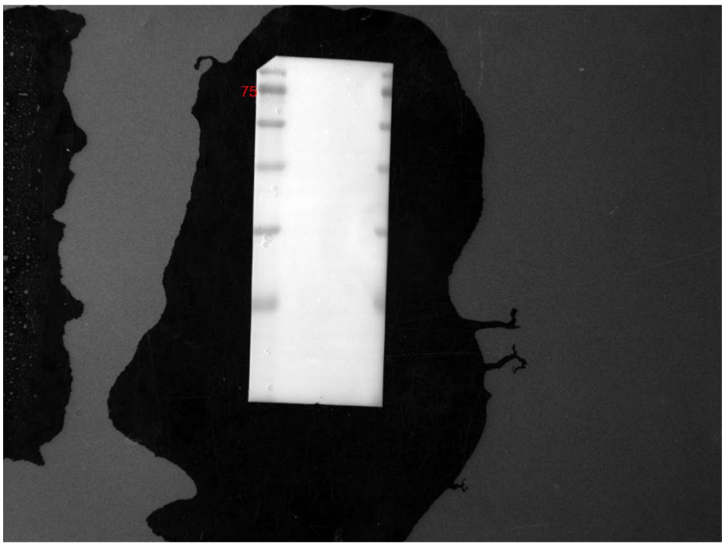

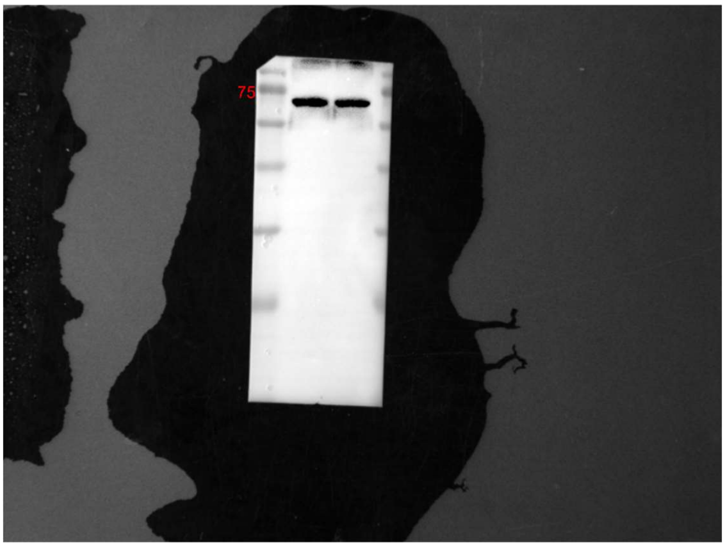

Figure 7D(AKT)

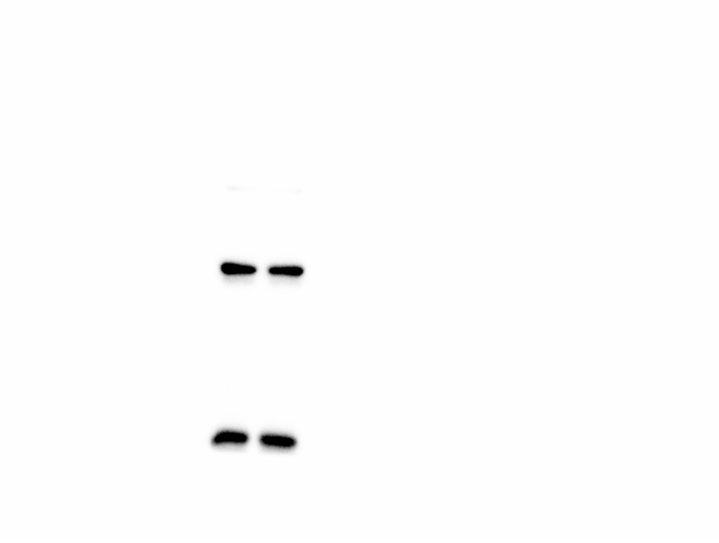

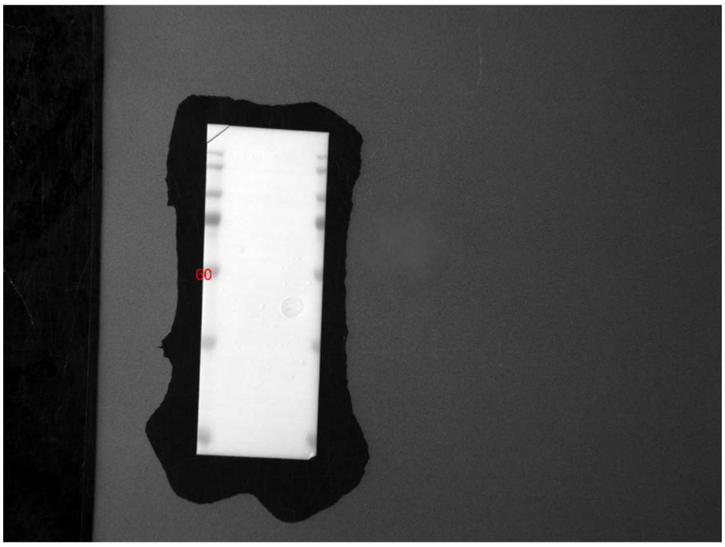

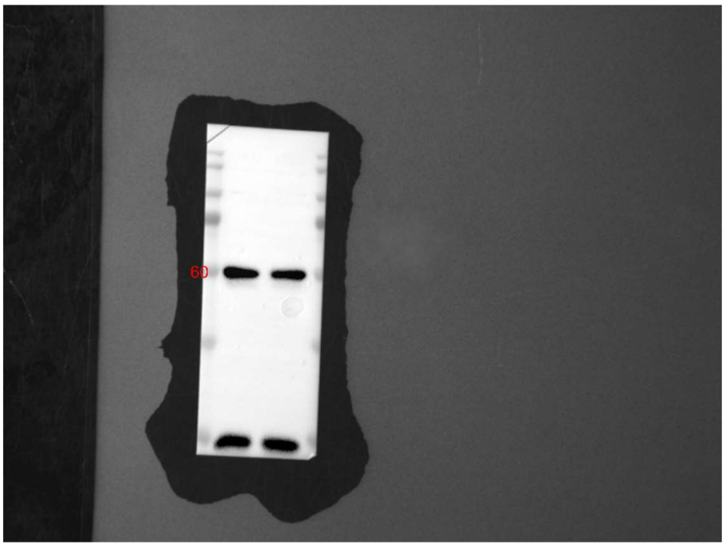

Figure 7D(p-AKT)

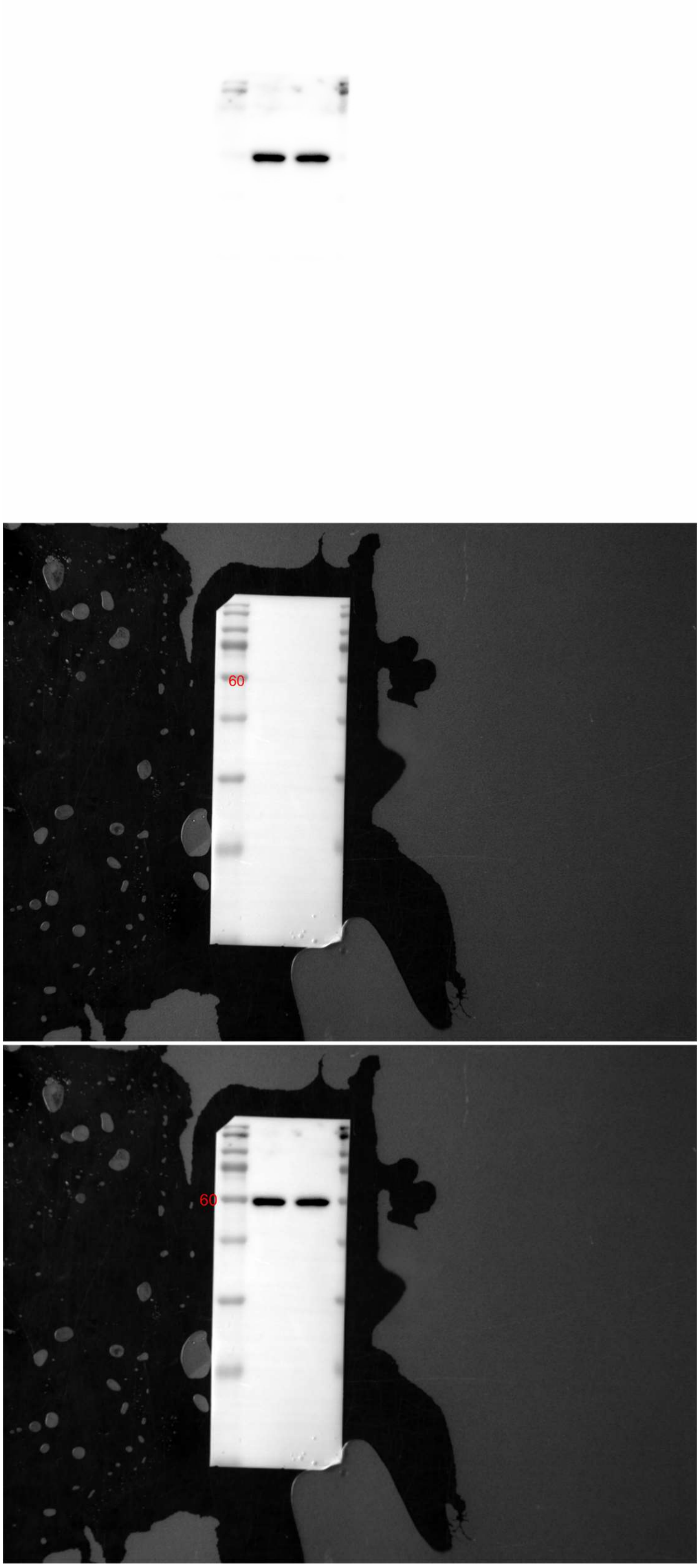

Figure 7D(tubulin)

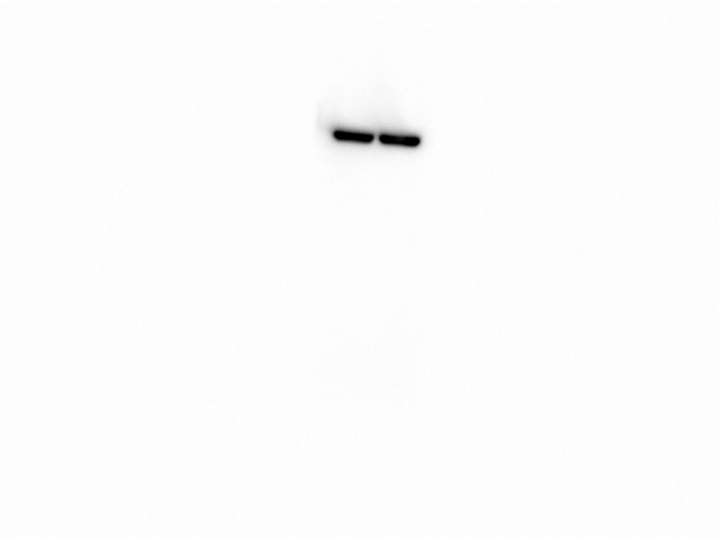

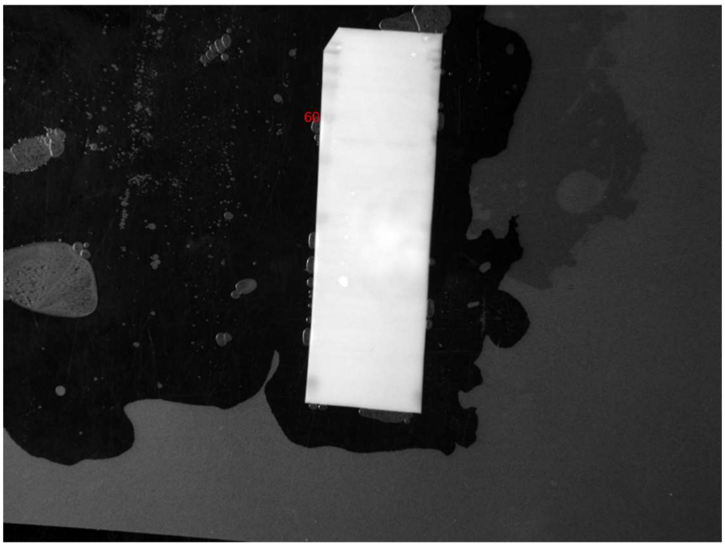

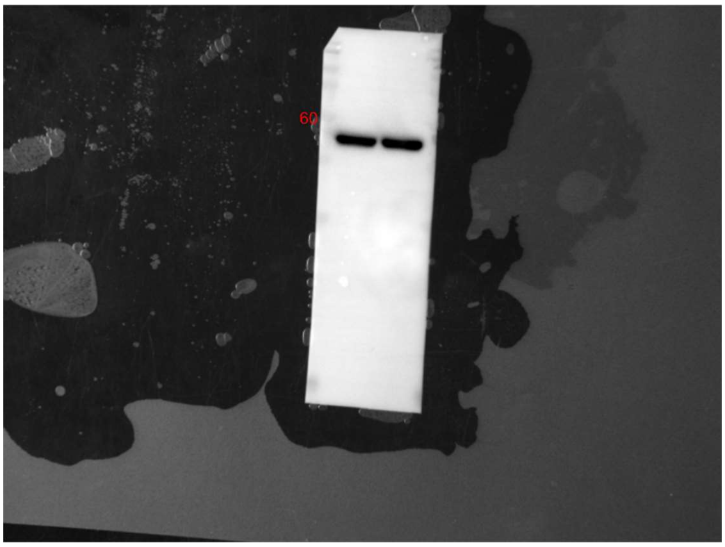

Figure 7E(LDHA)

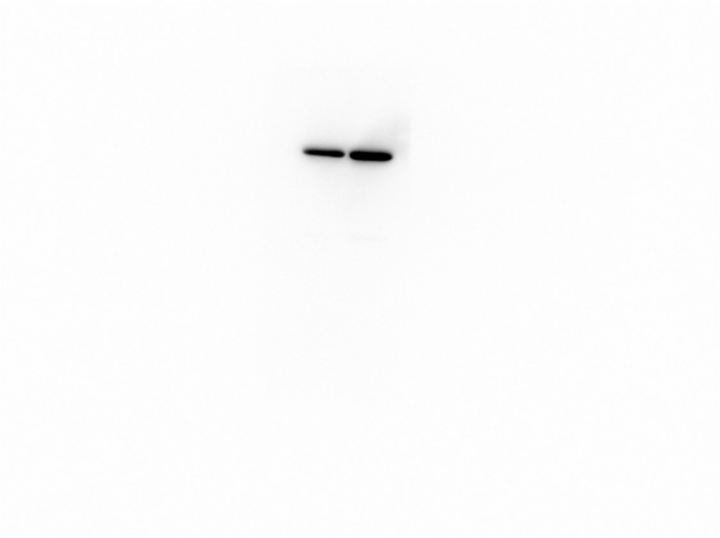

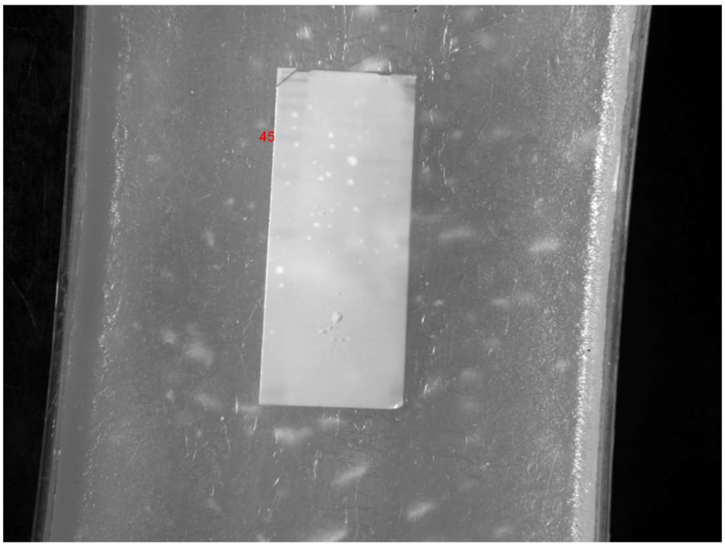

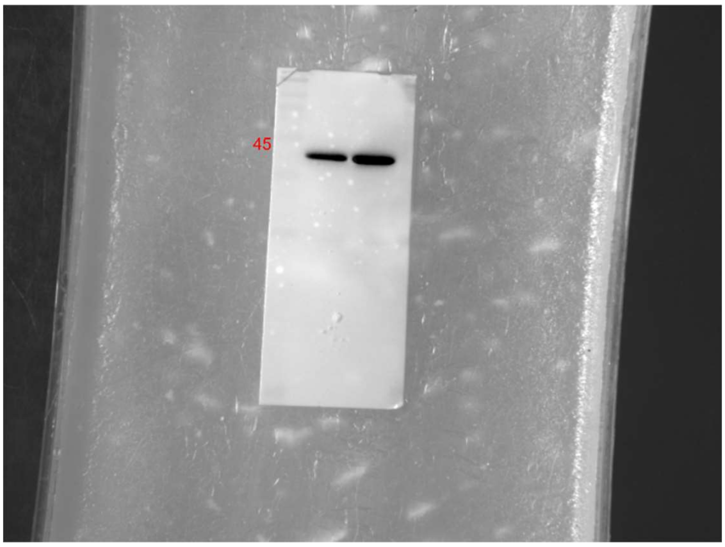

Figure 7E(ALODA)

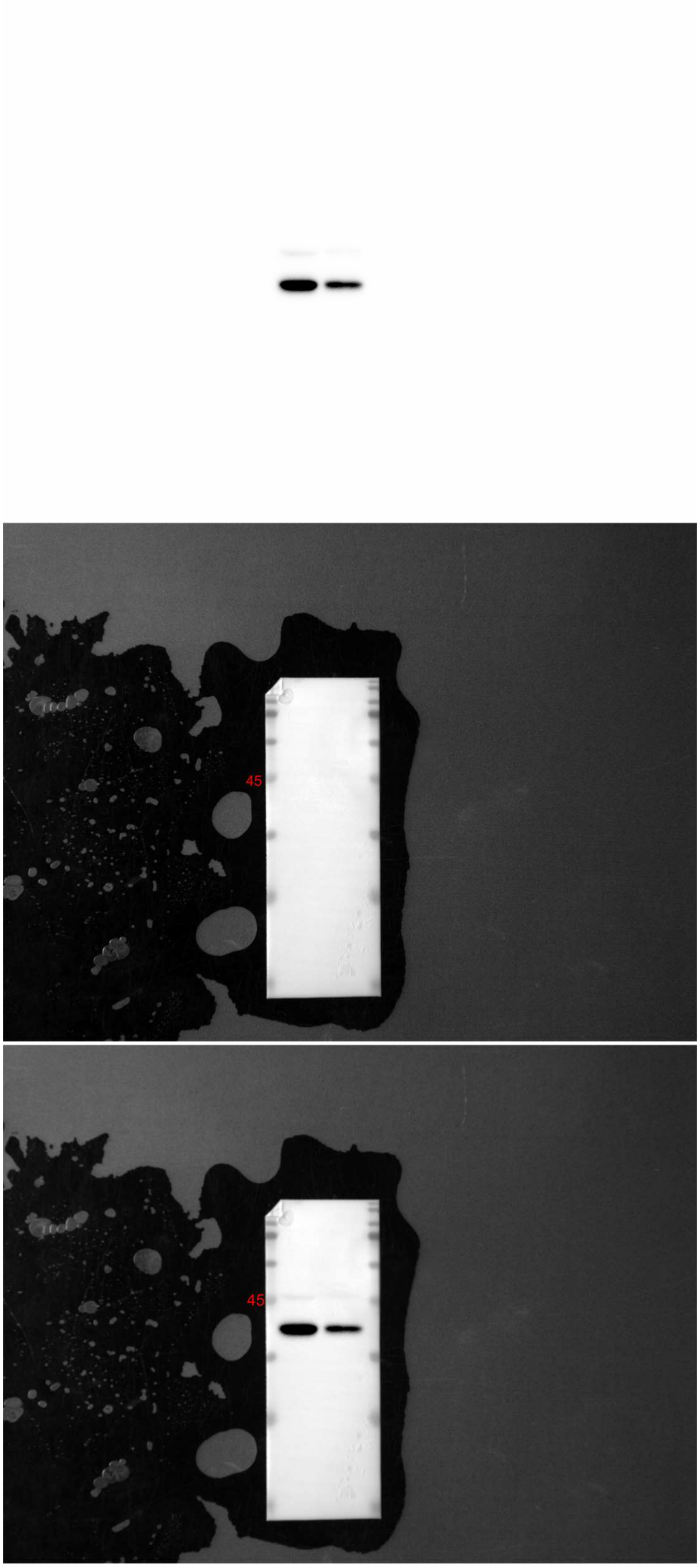

Figure 7E(p-PDHK1)

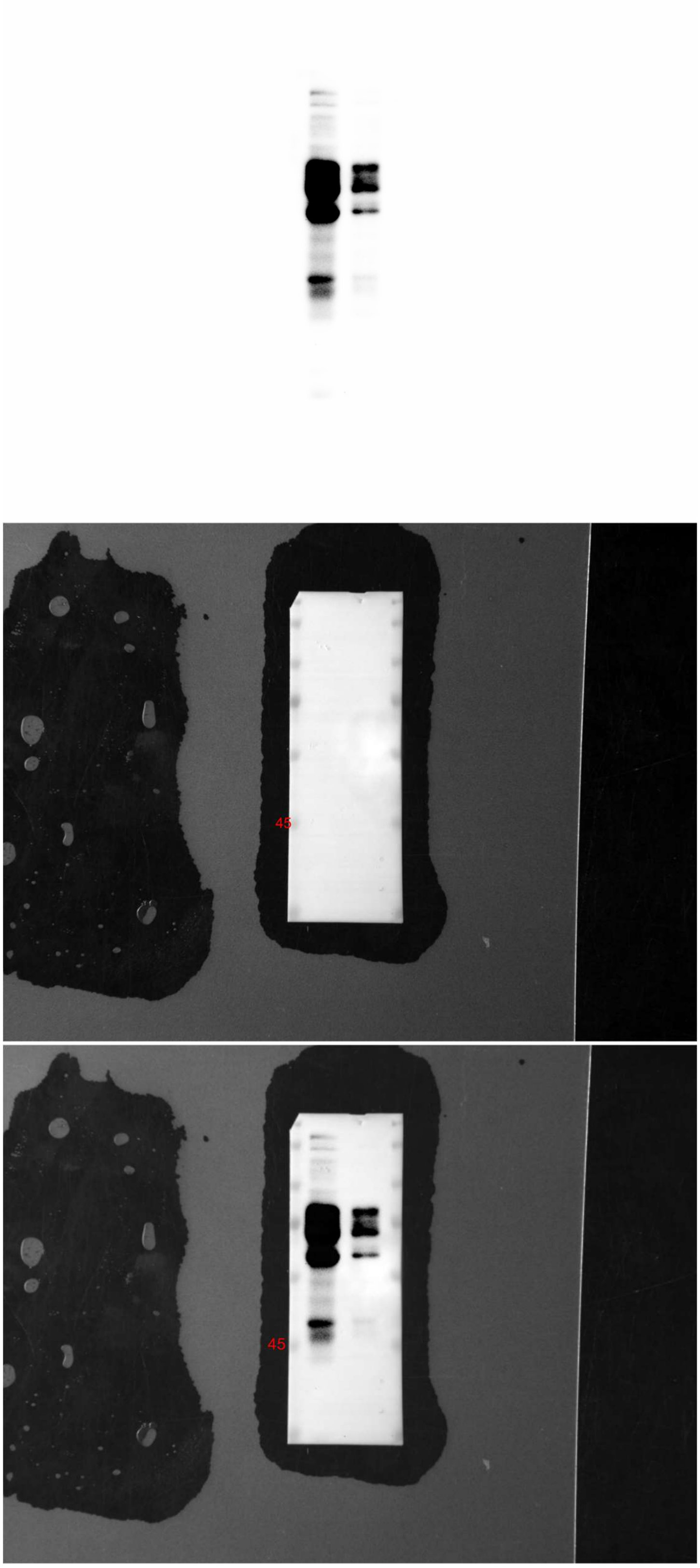

Figure 7E(HK2)

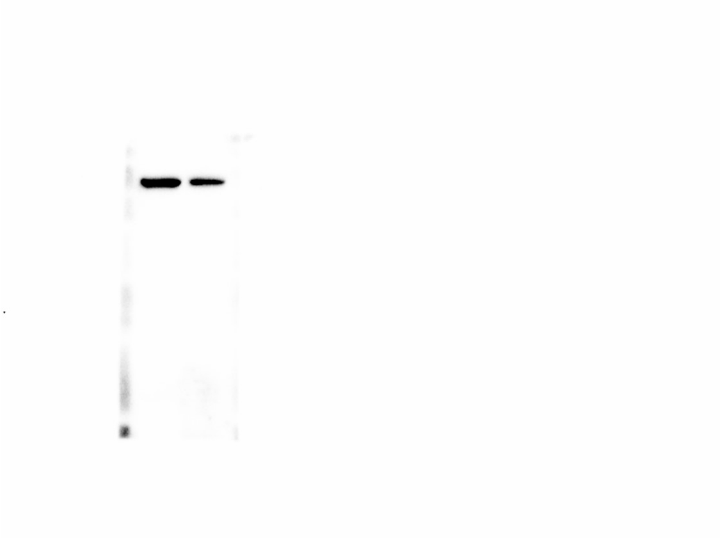

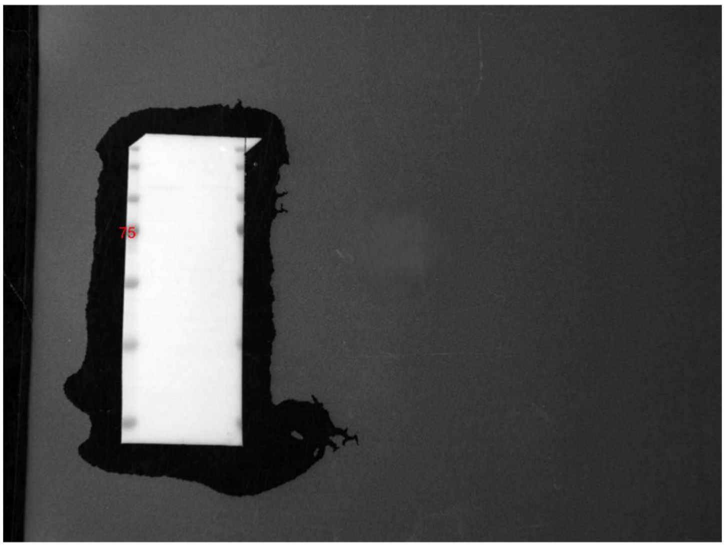

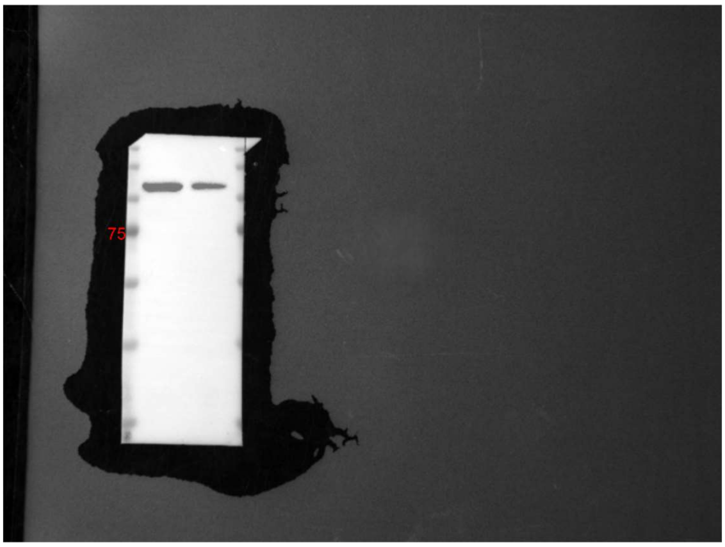

Figure 7E(ACLY)

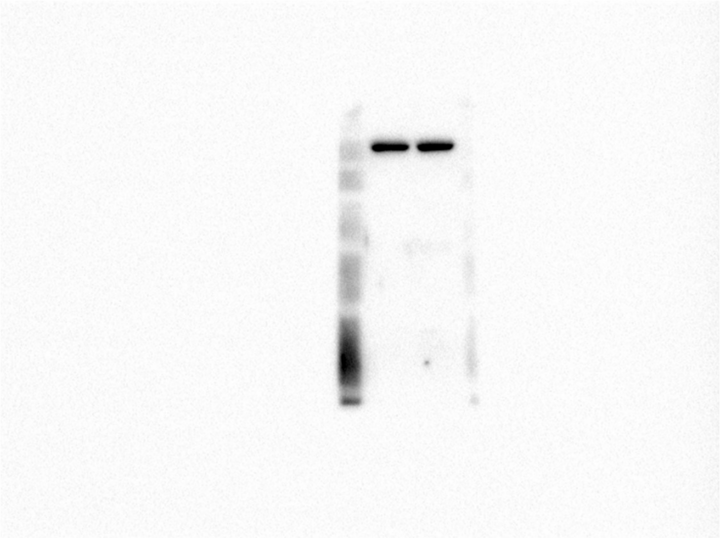

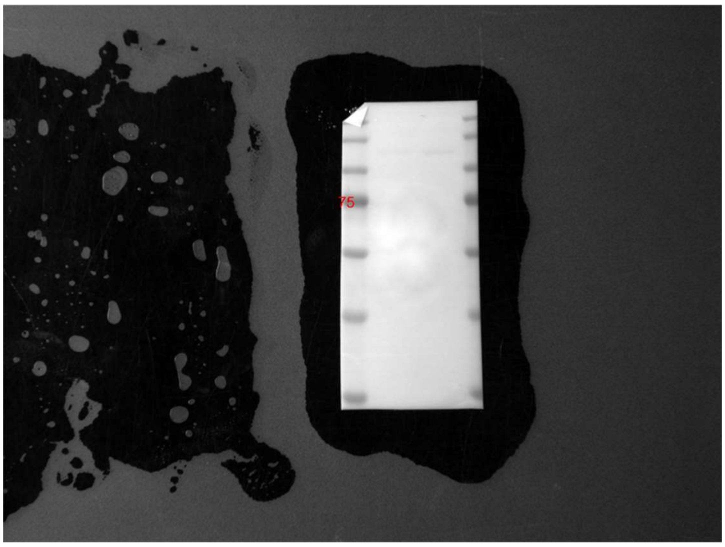

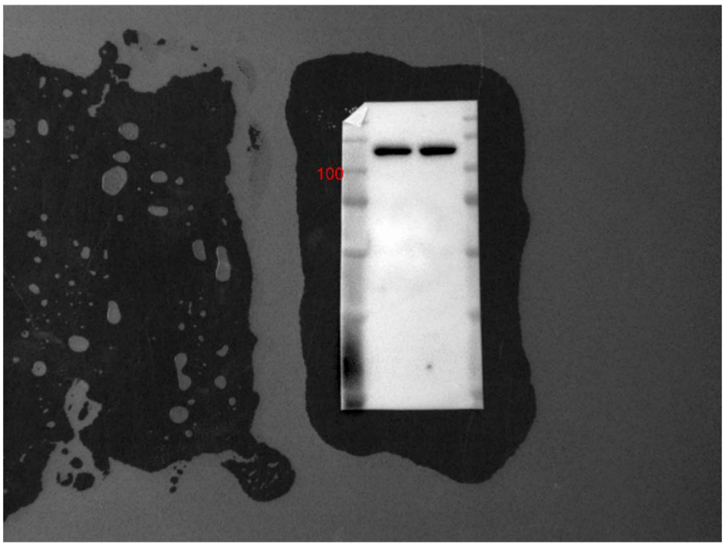

Figure 7E(p-ACLY)

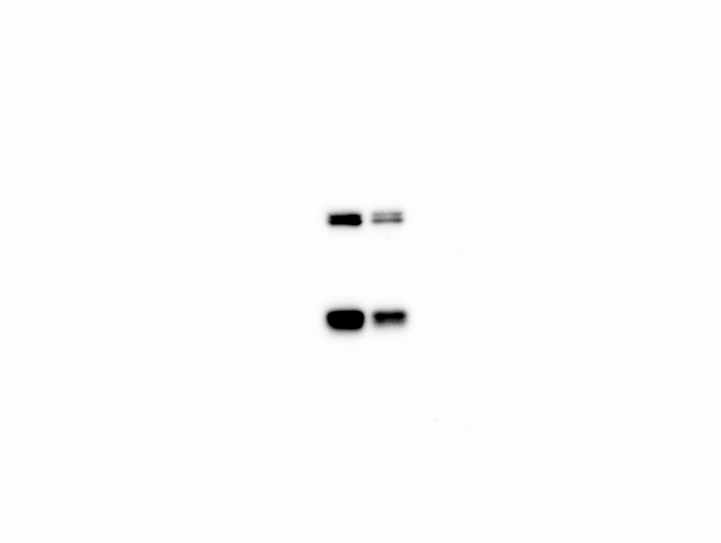

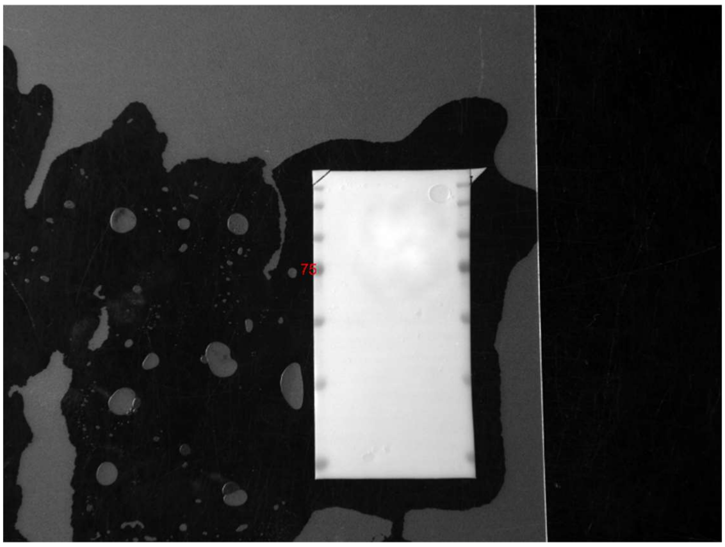

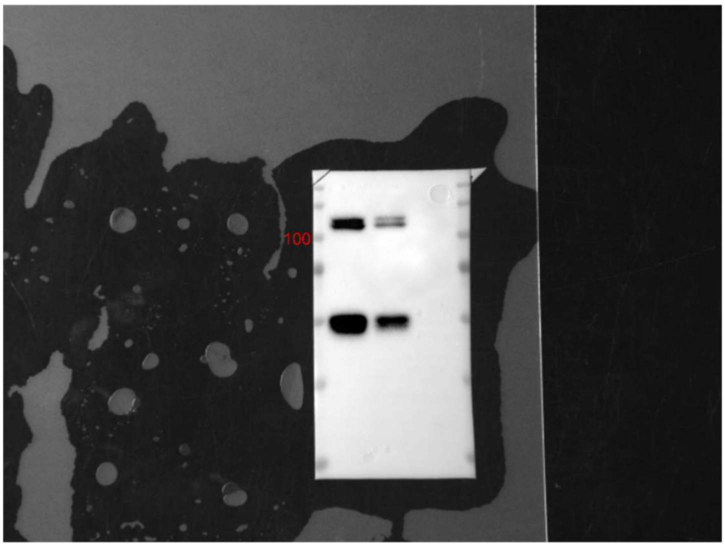

Figure 7E(Tubulin)

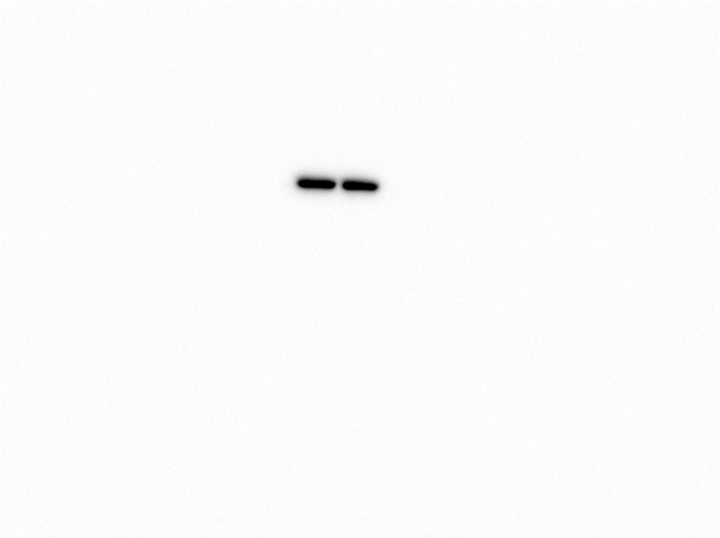

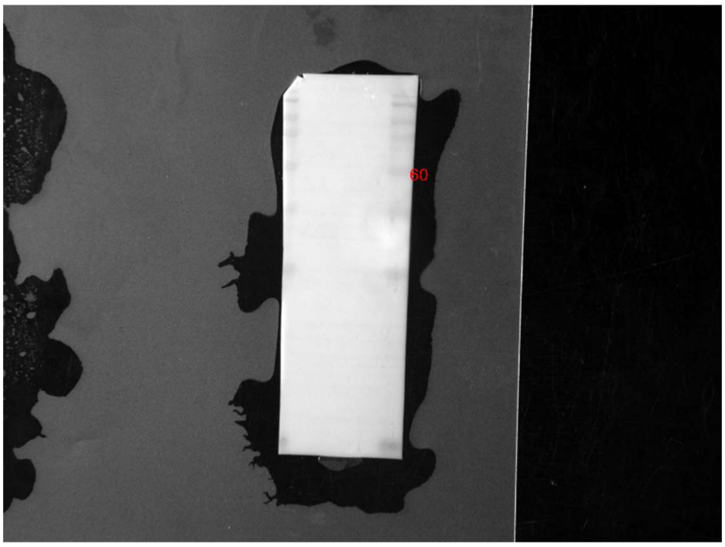

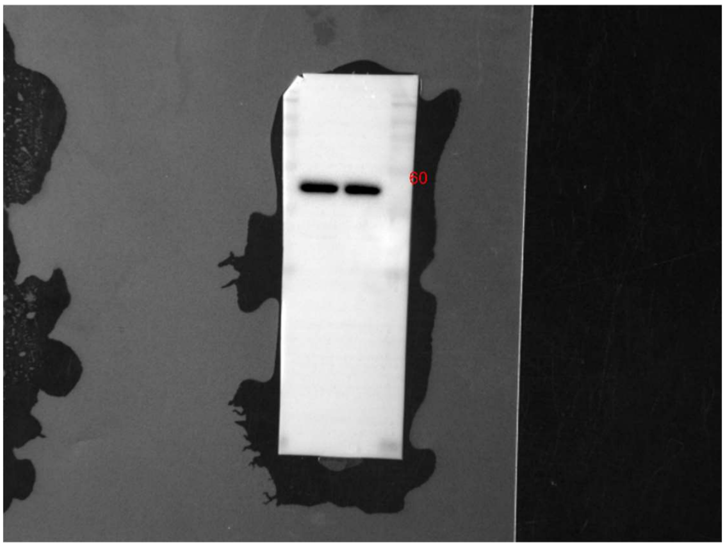

Figure 8E(CDK1)

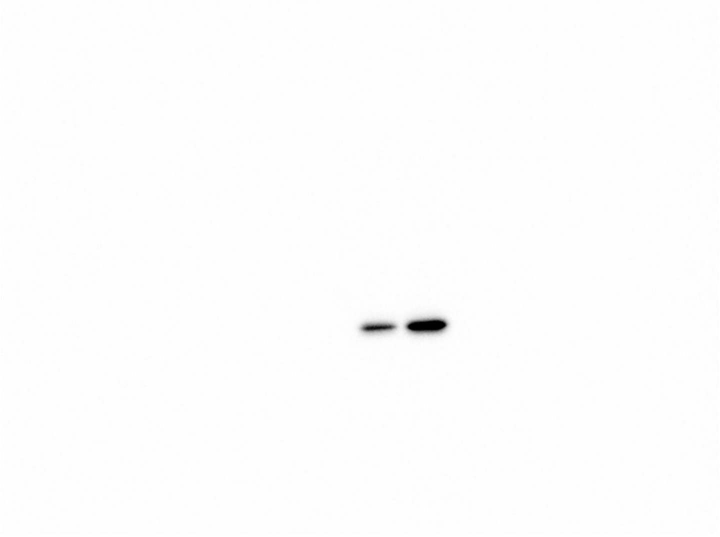

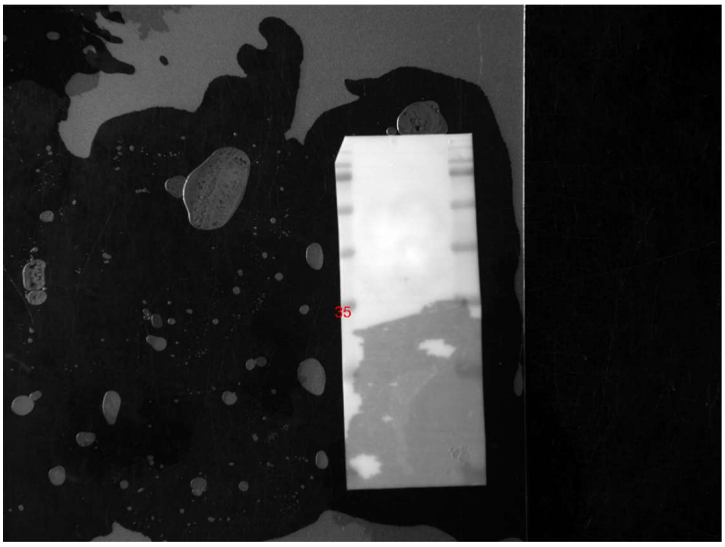

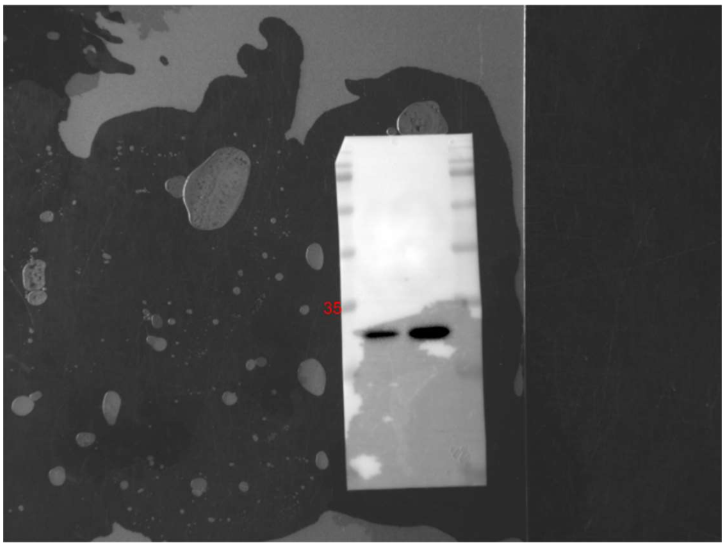

Figure 8E(CDK2)

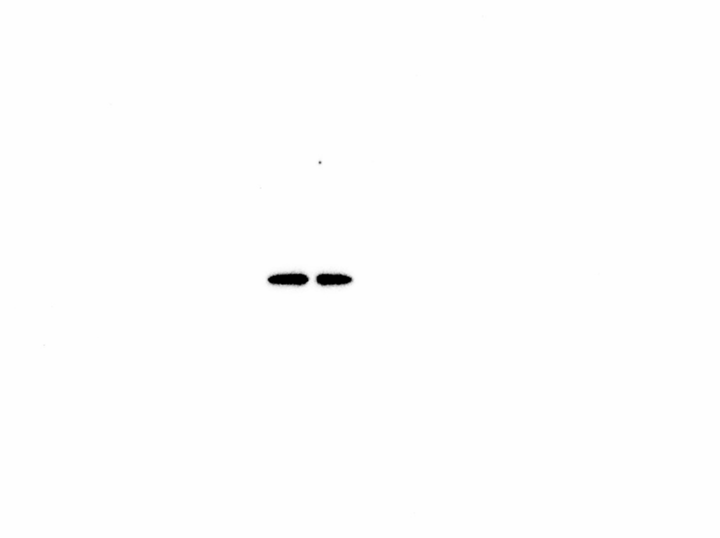

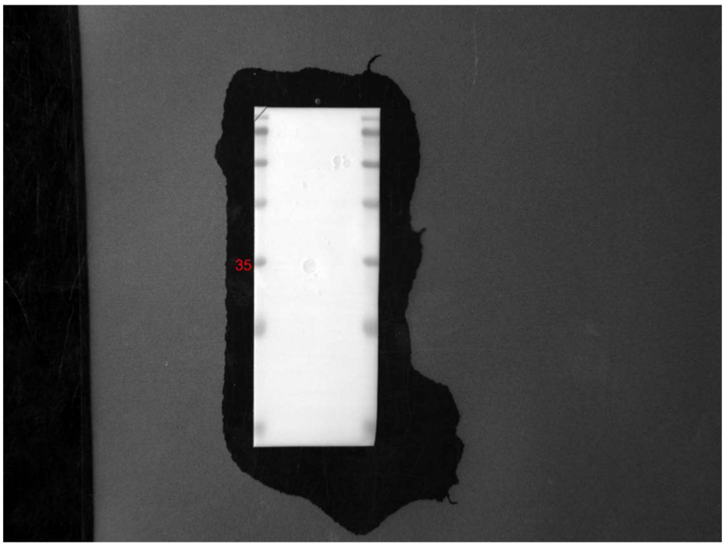

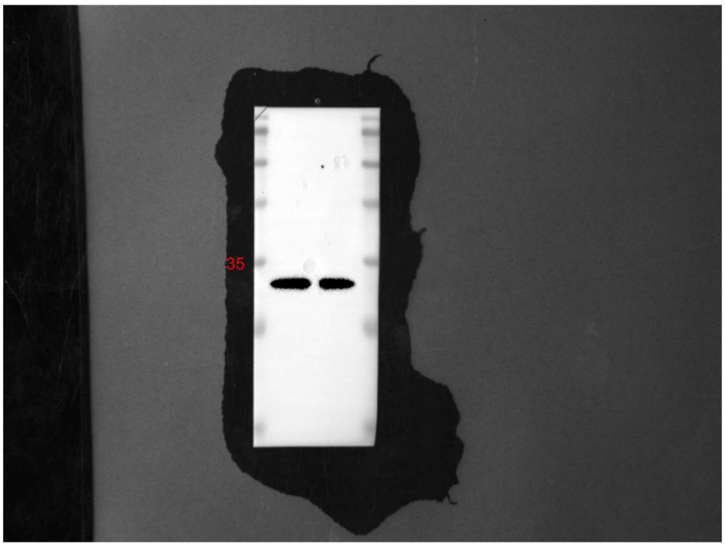

Figure 8E(CDK4)

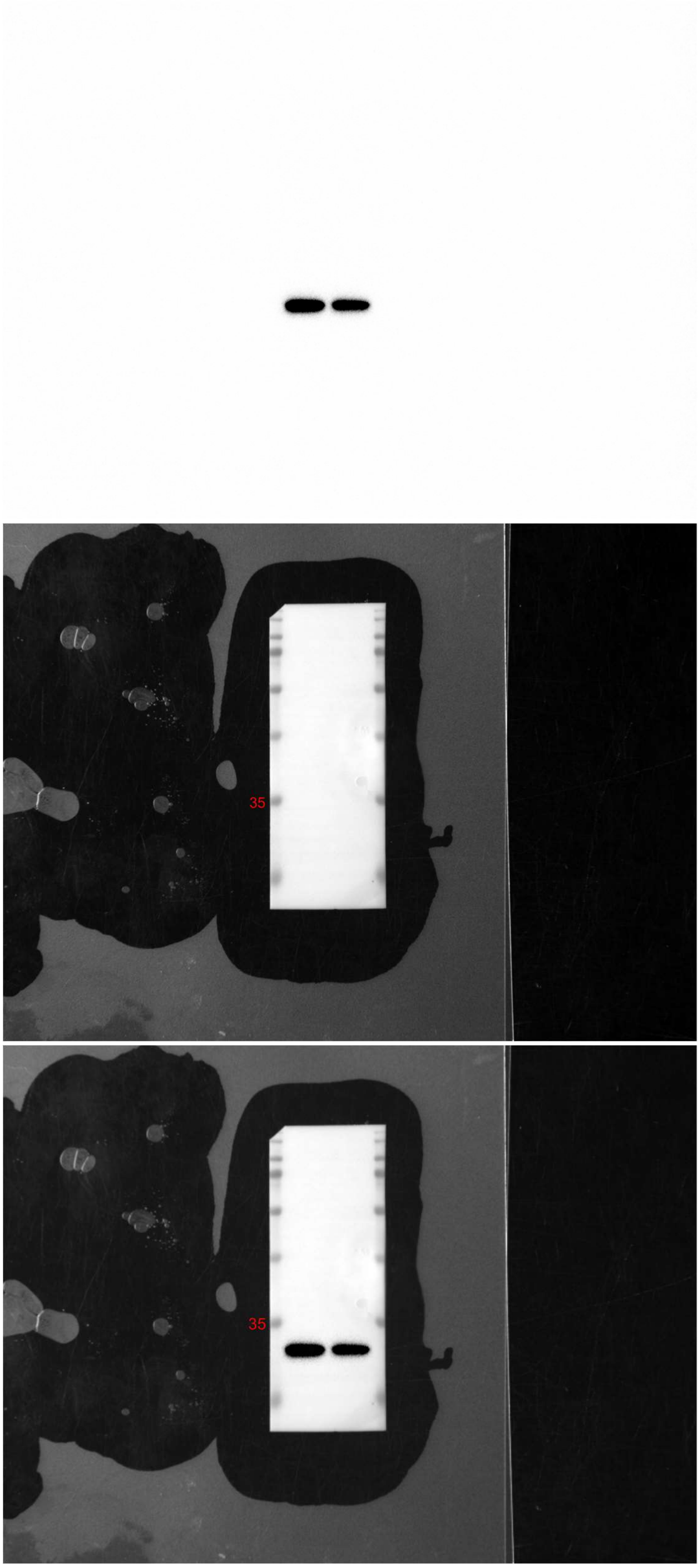

Figure 8E(CDK6)

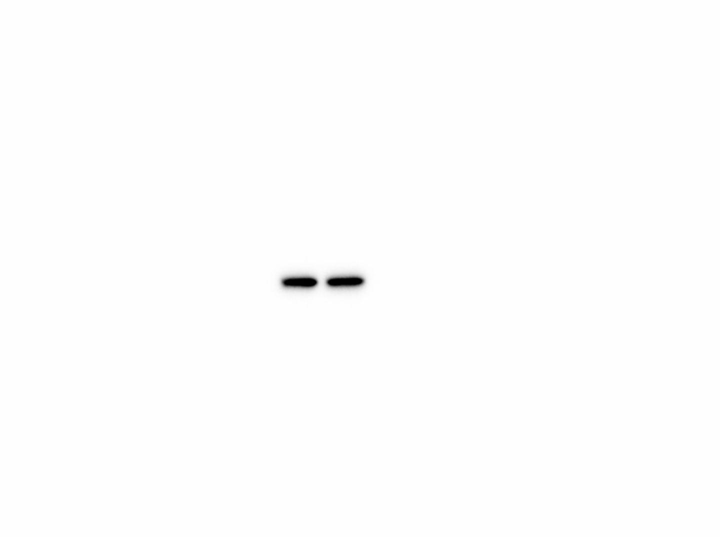

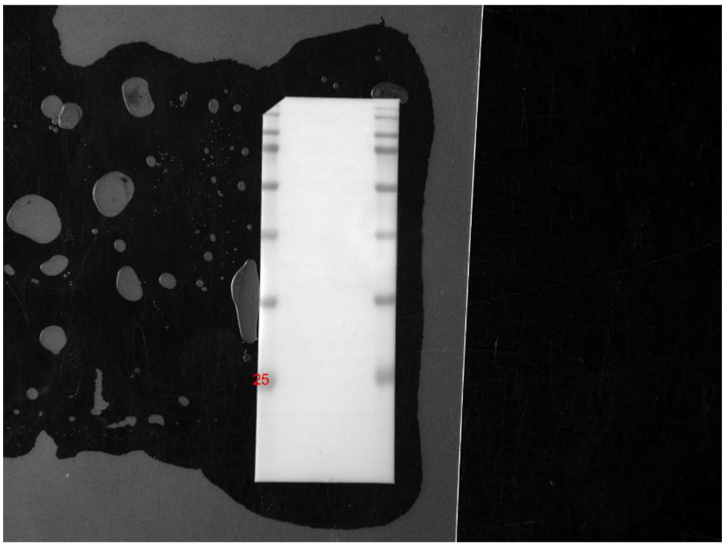

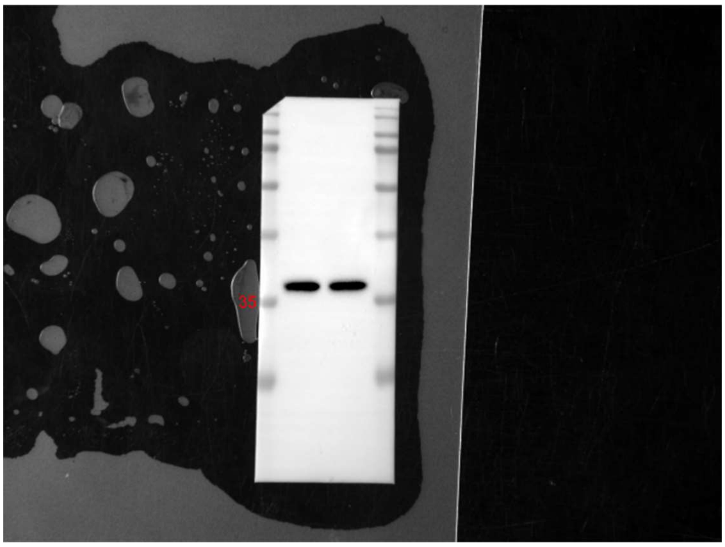

Figure 8E(CDK7)

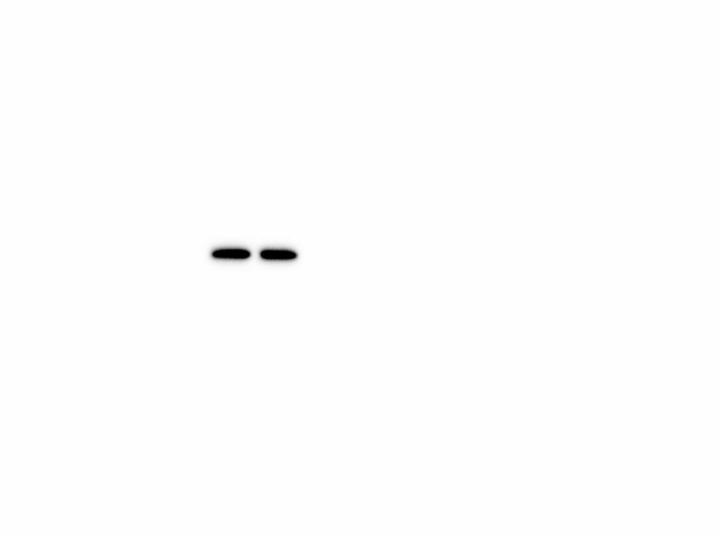

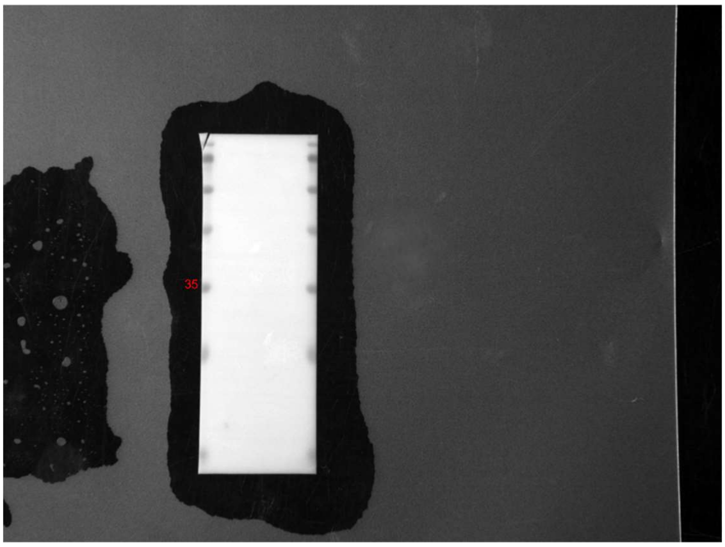

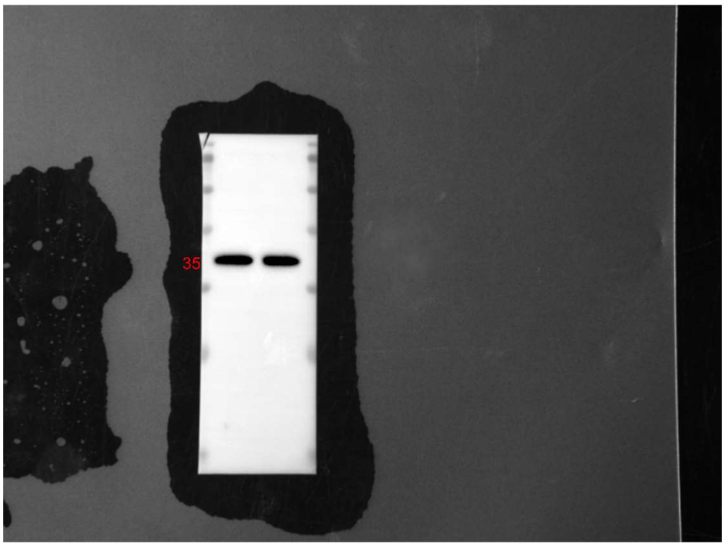

Figure 8E(CDK9)

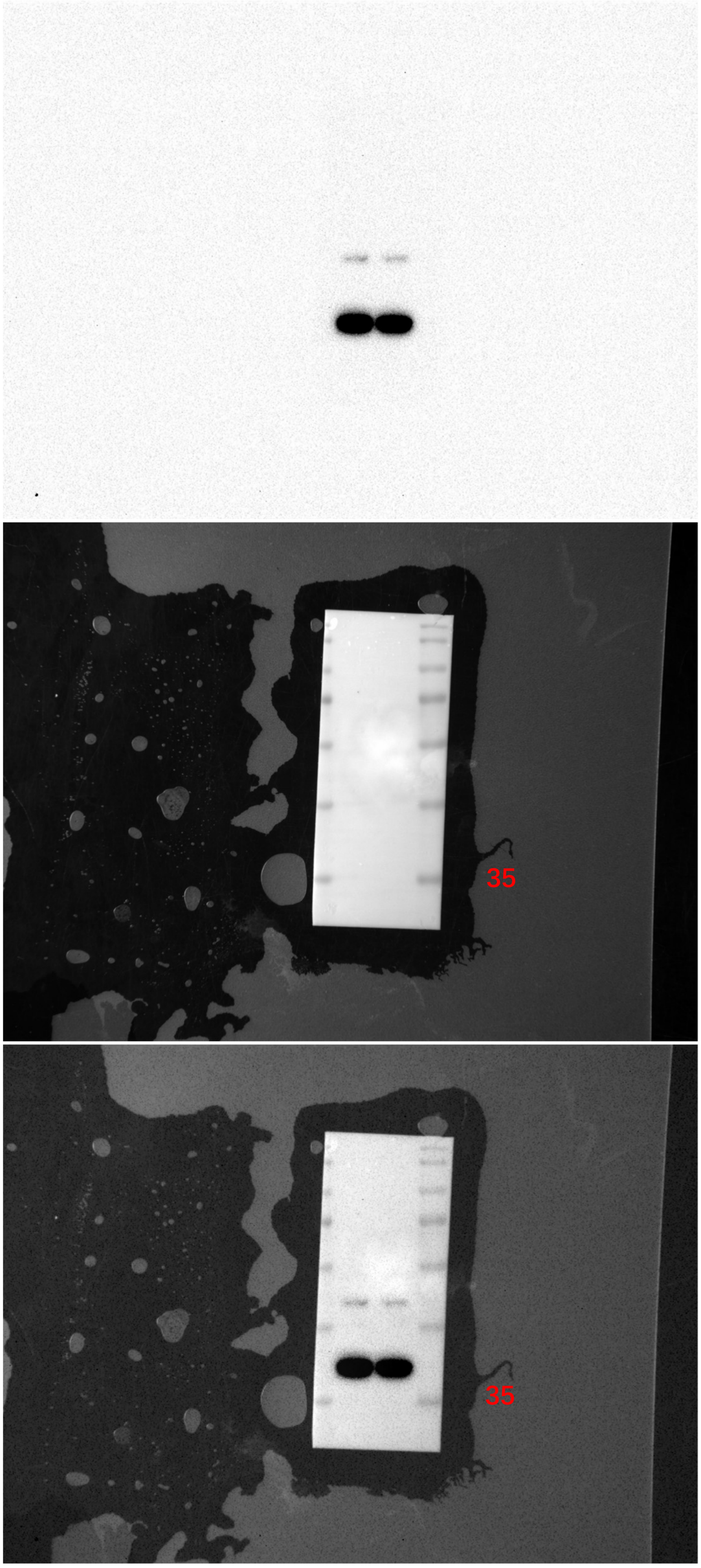

Figure 8E(Tubulin)

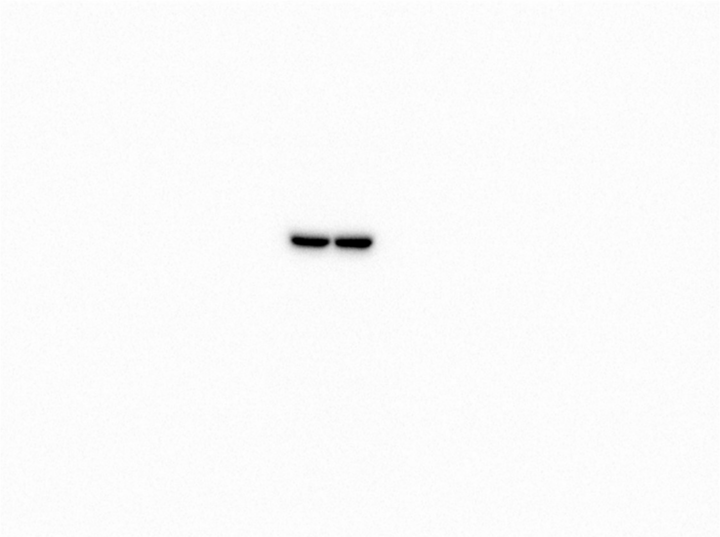

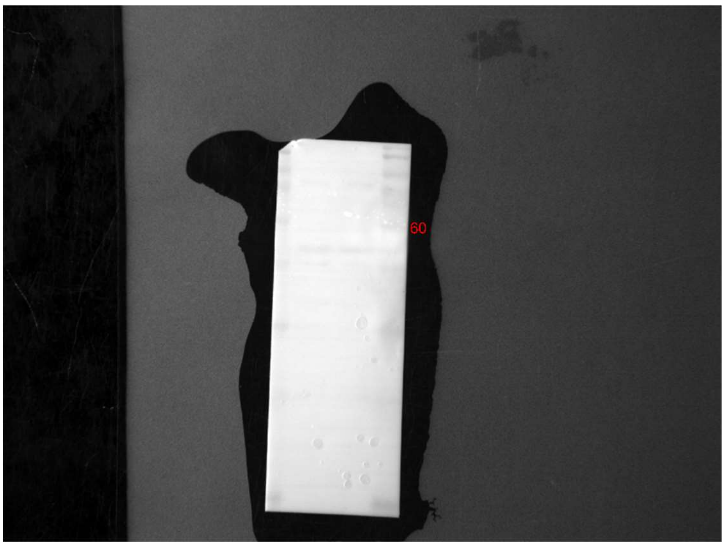

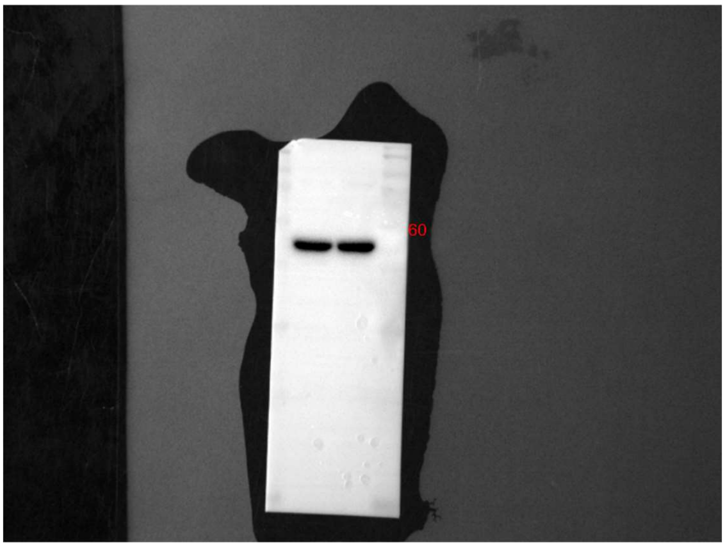

Figure 8E(P65)

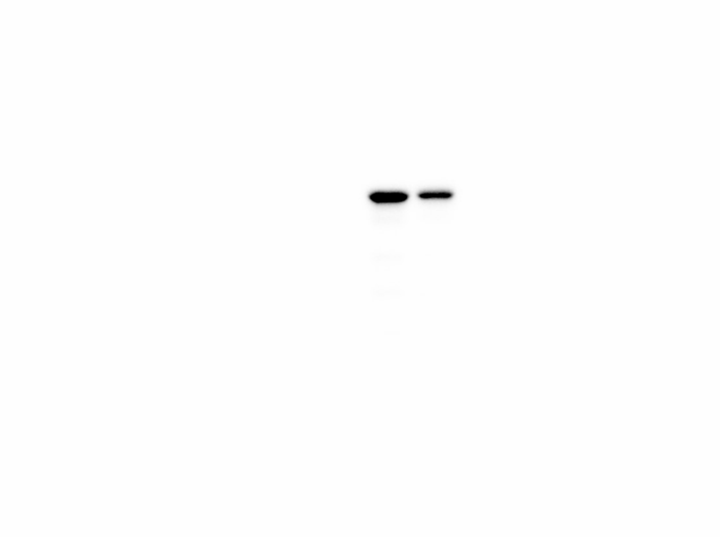

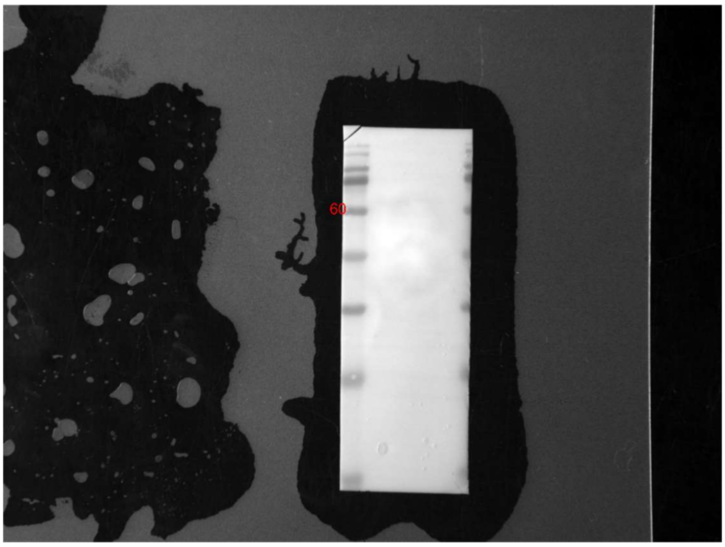

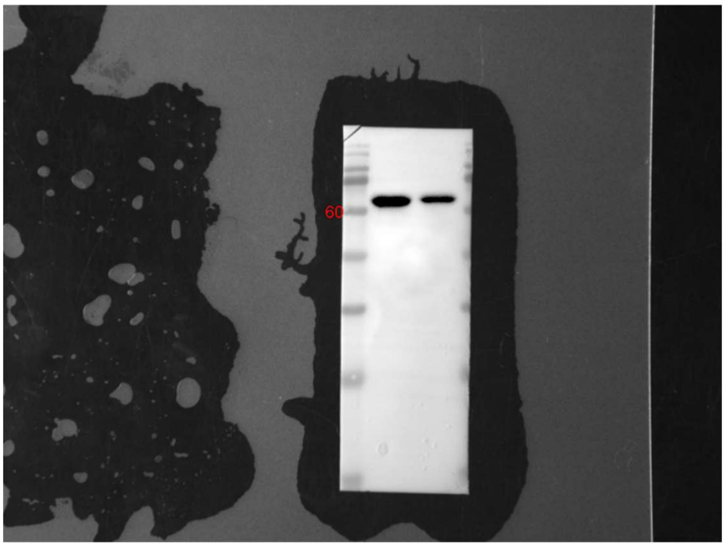

Figure 8E(Bcl2)

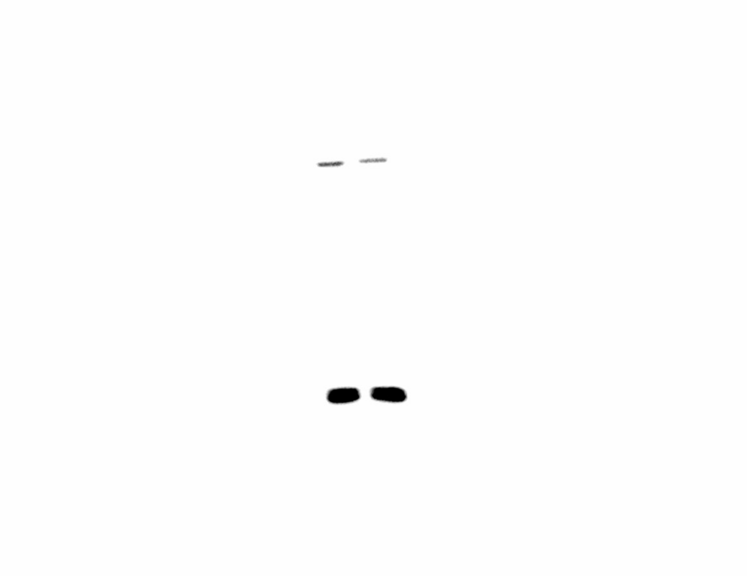

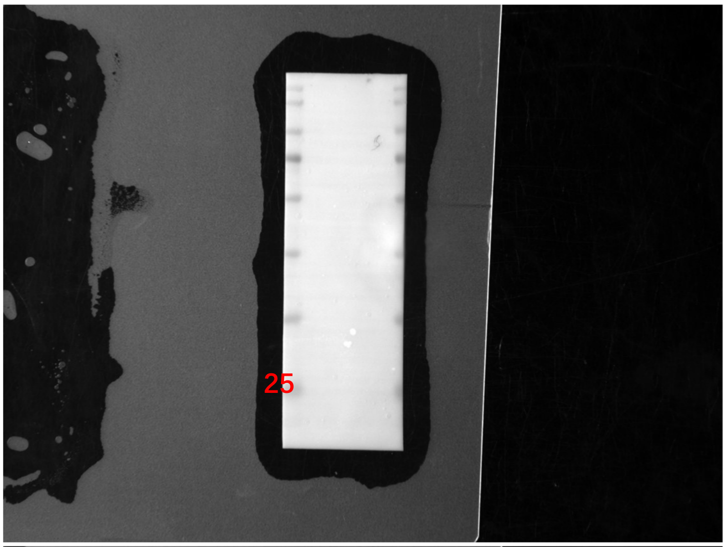

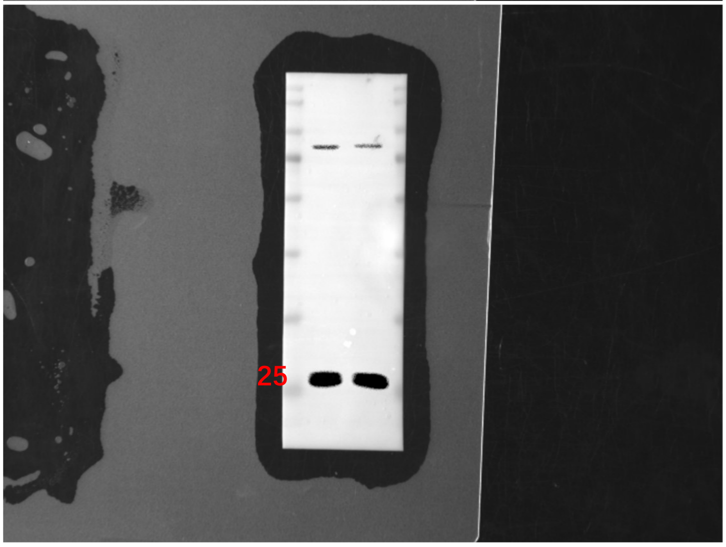

Figure 8E(Caspase3)

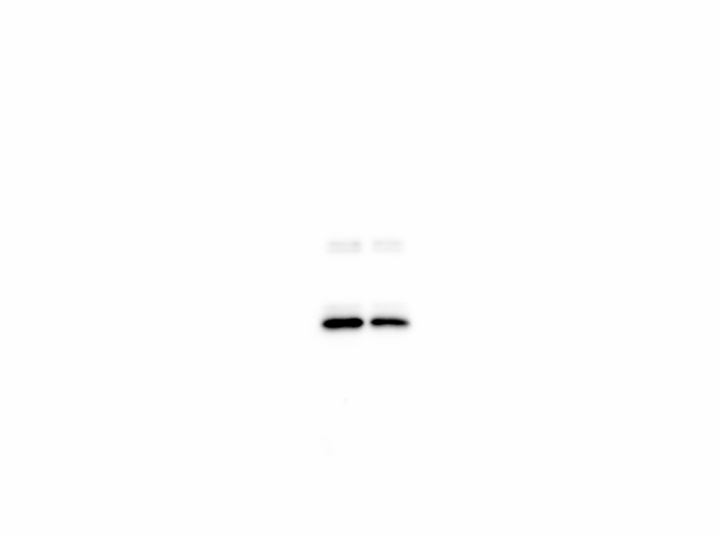

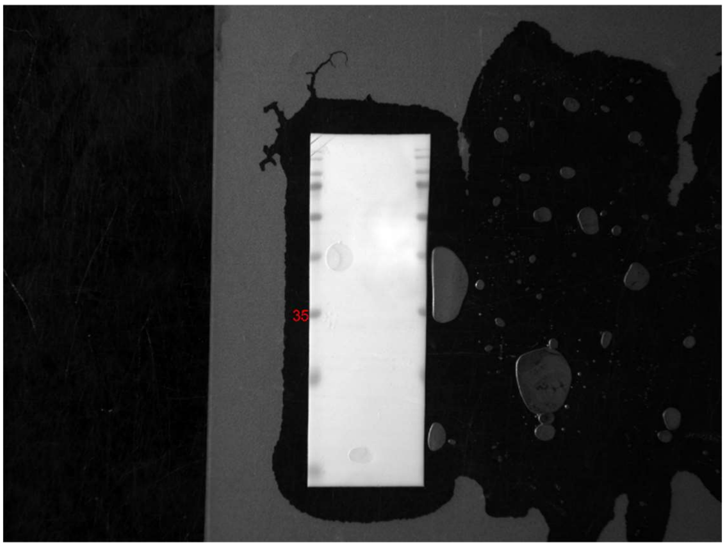

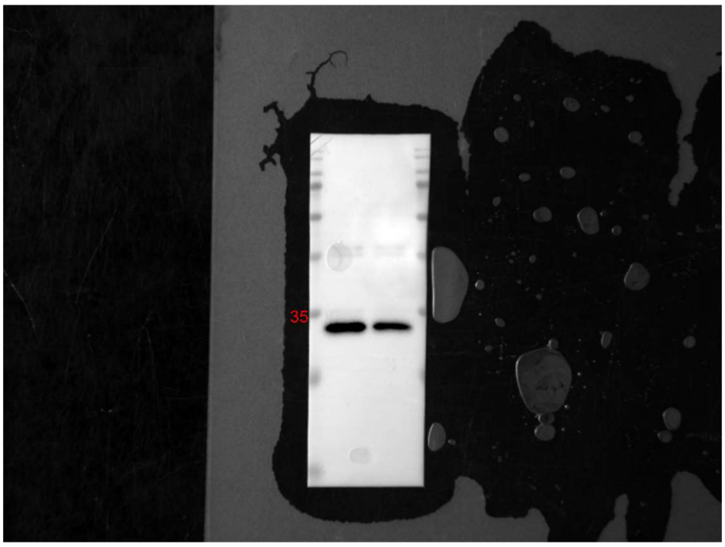

Figure 8E(P53)

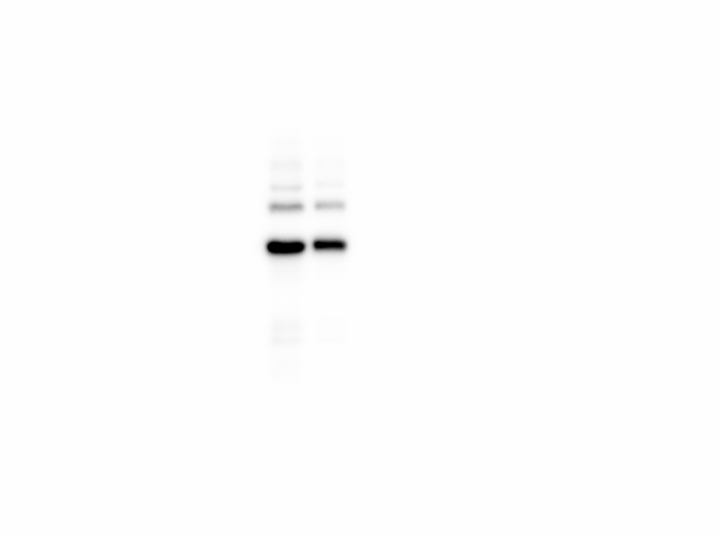

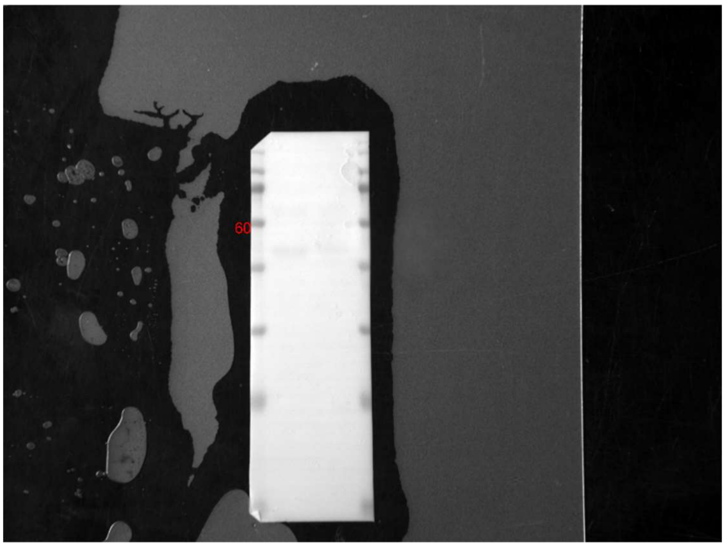

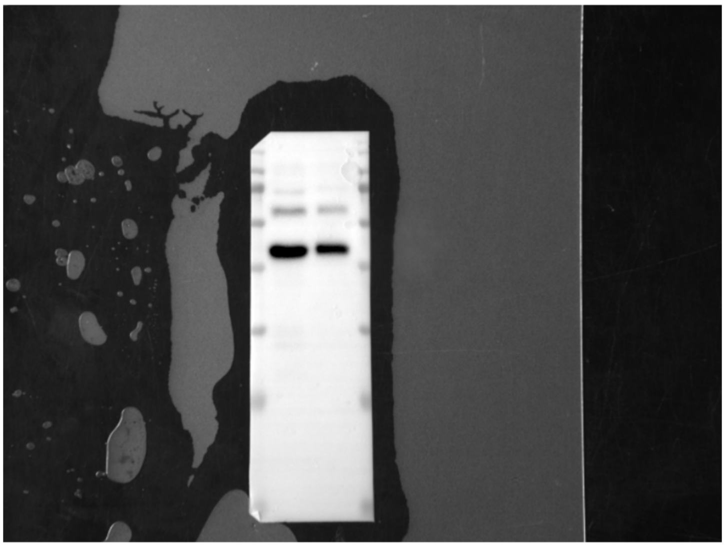

Figure 8E(p-Erk)

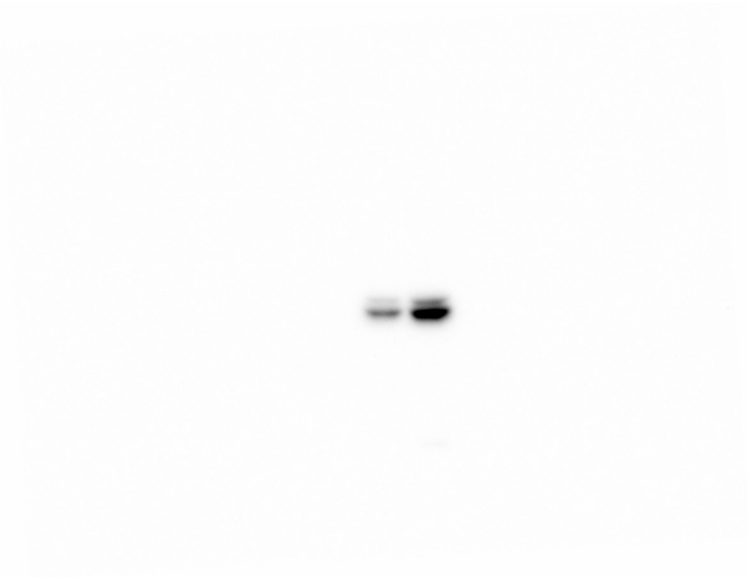

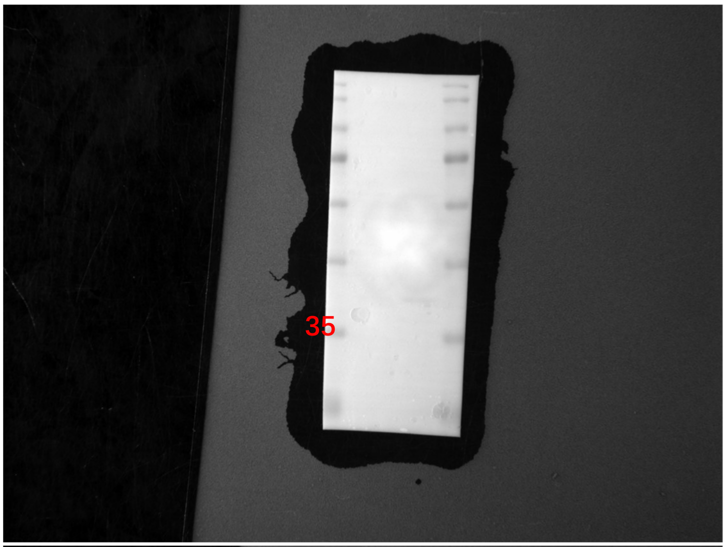

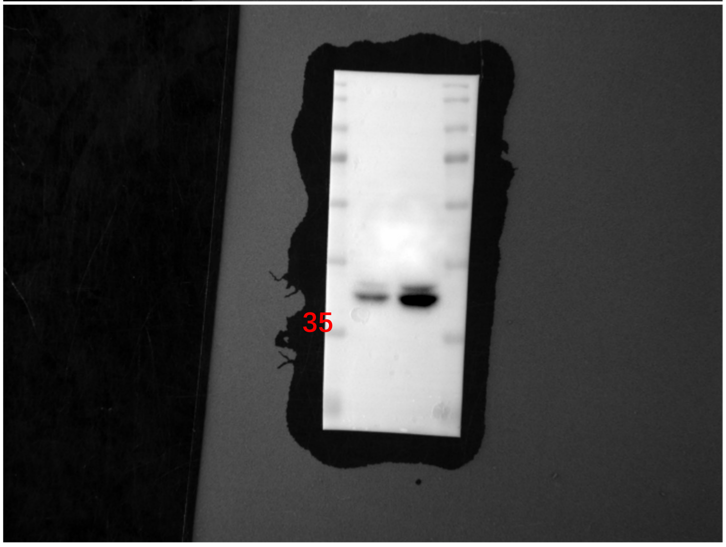

Figure 8E(Erk)

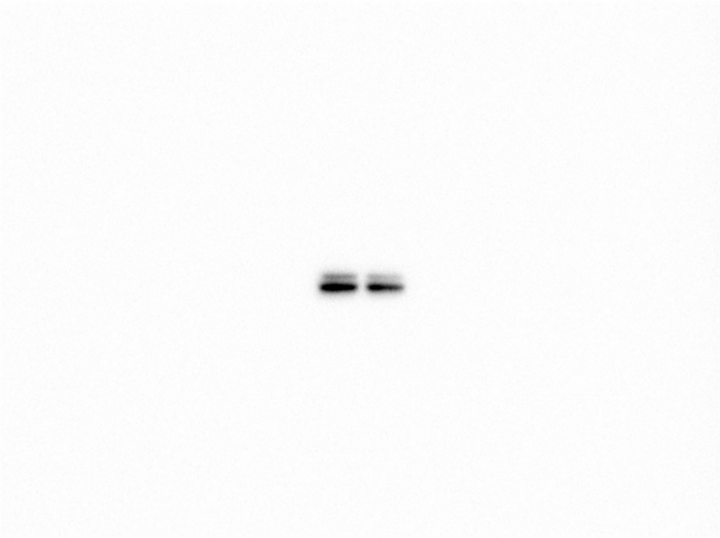

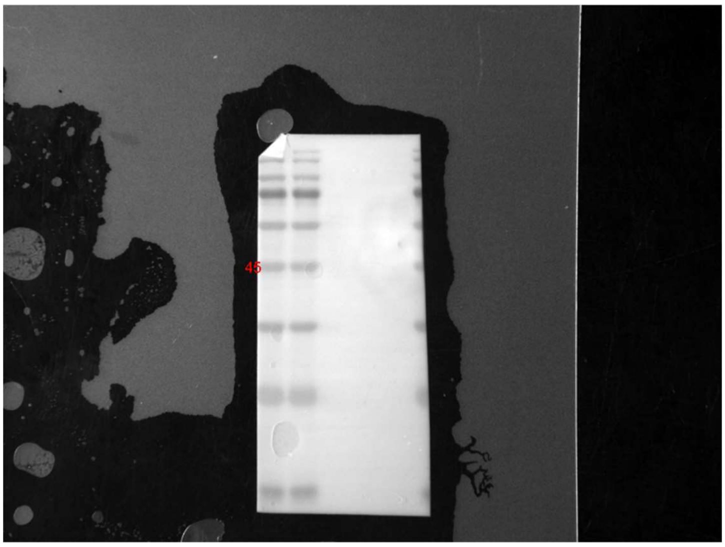

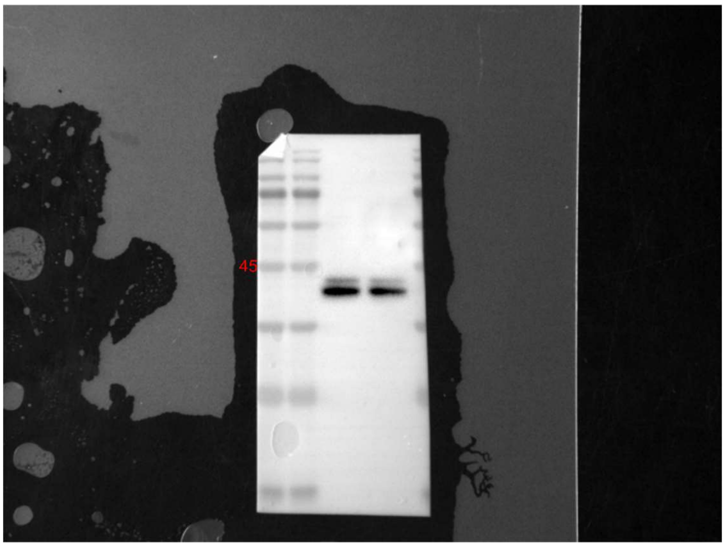

Figure 8E(Tubulin)

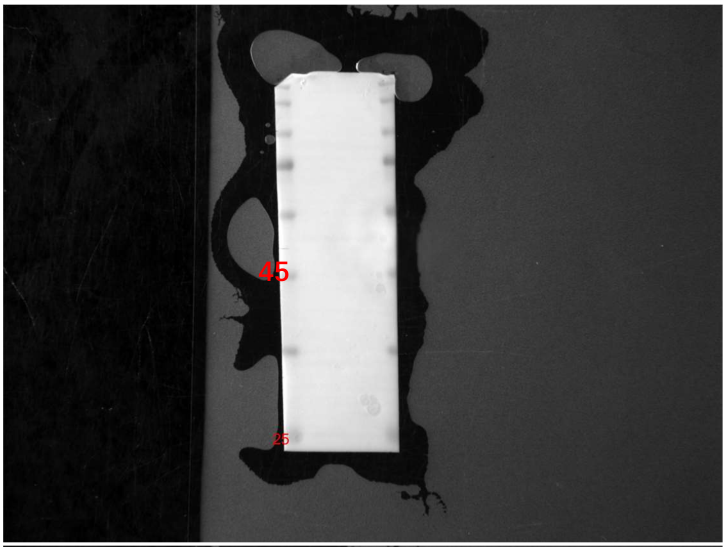

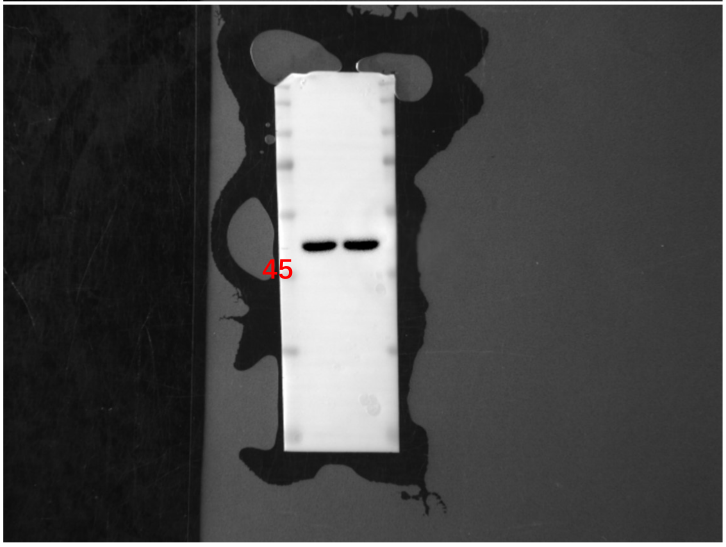

Figure 8F(CDK1)

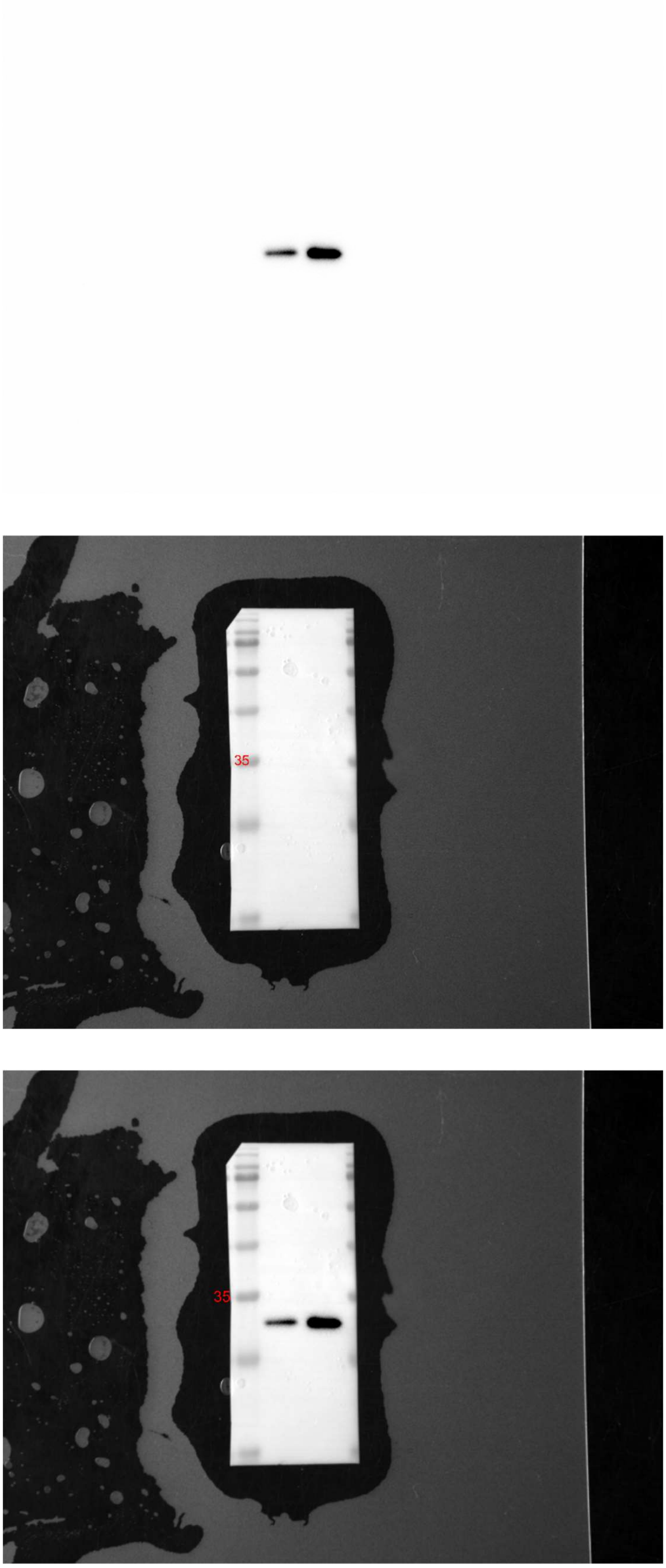

Figure 8F(CDK2)

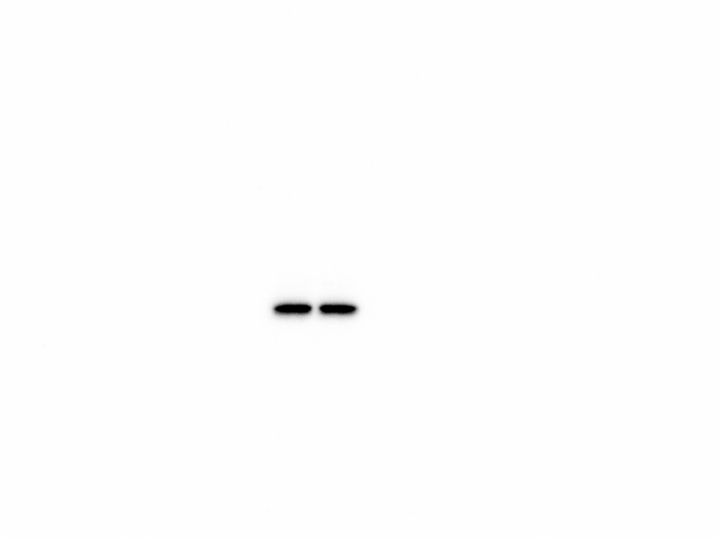

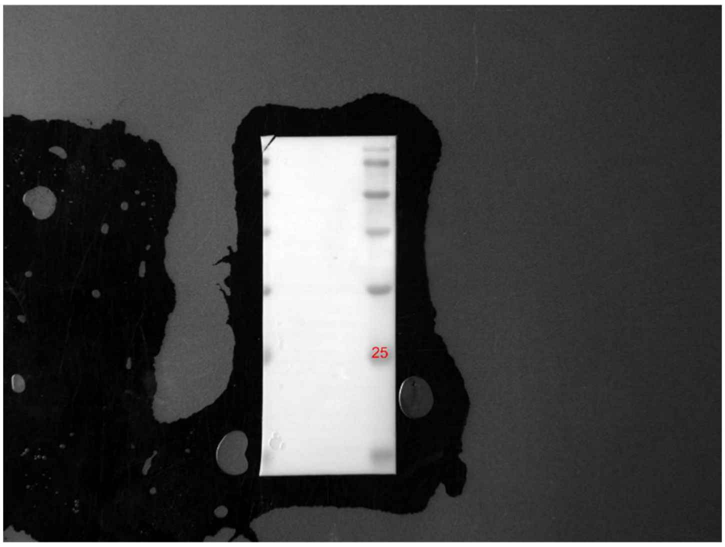

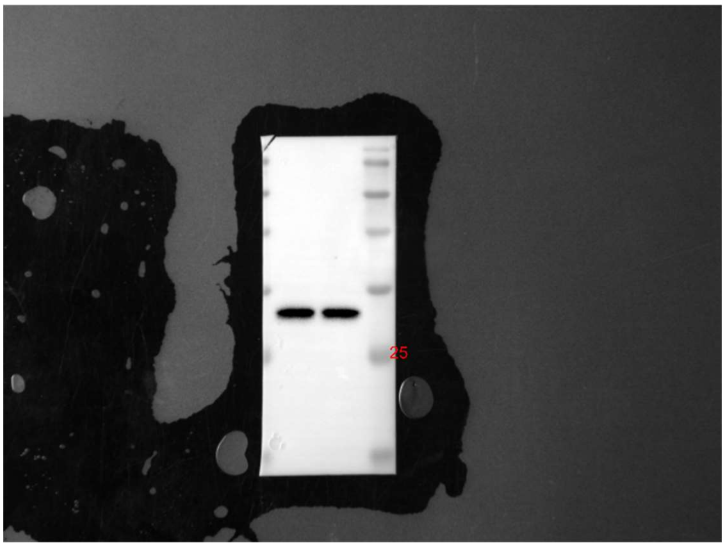

Figure 8F(CDK4)

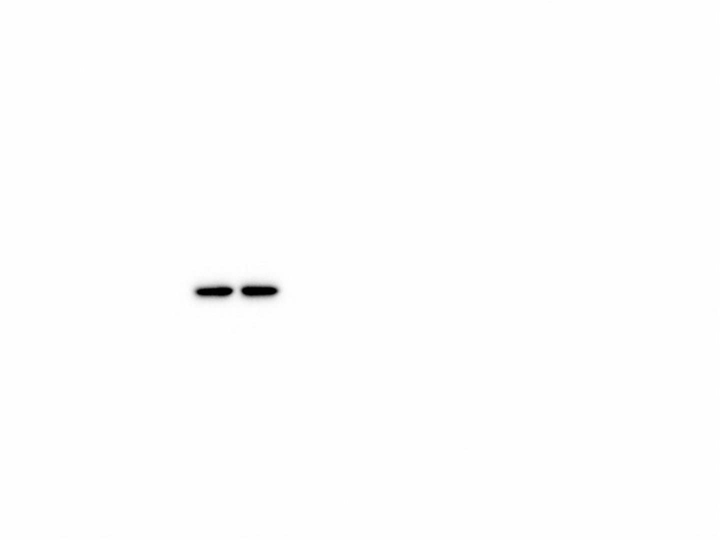

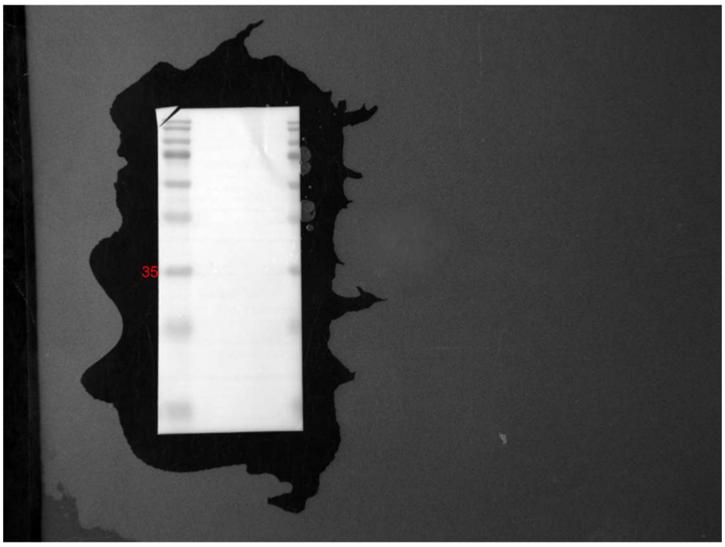

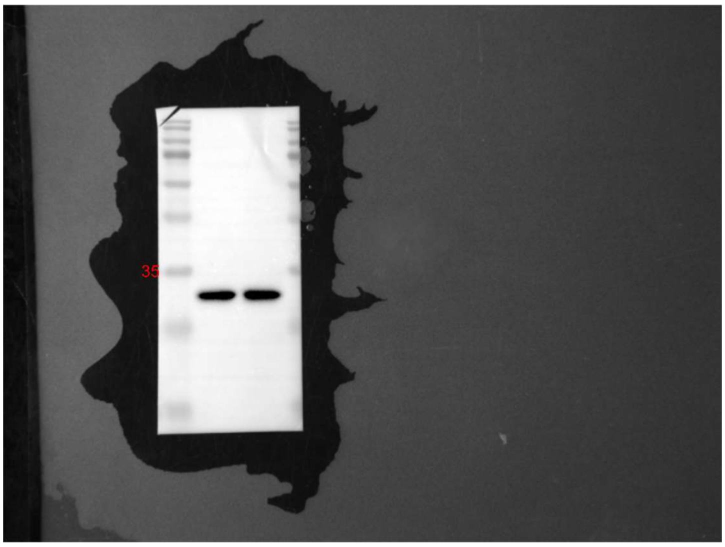

Figure 8F(CDK6)

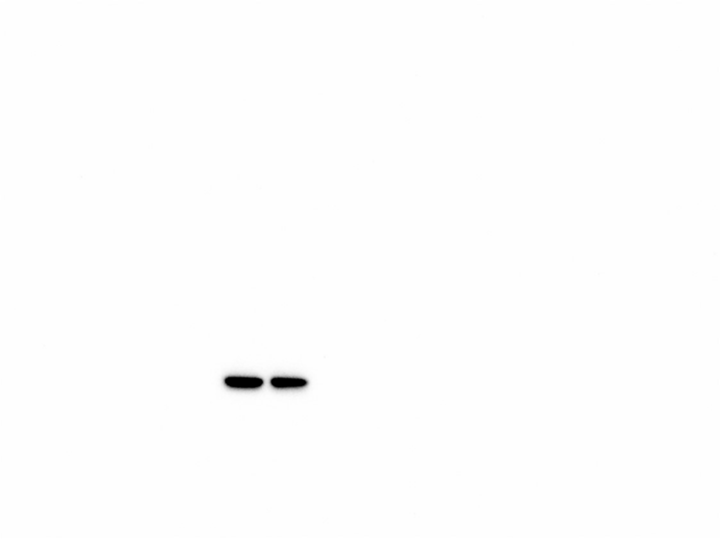

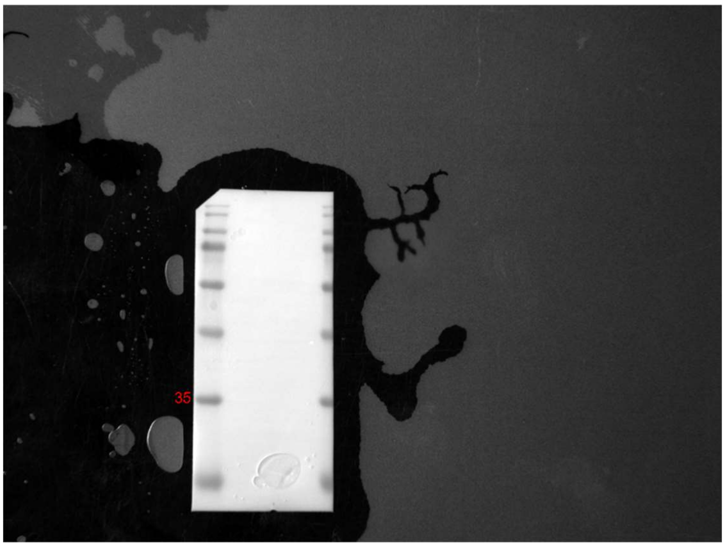

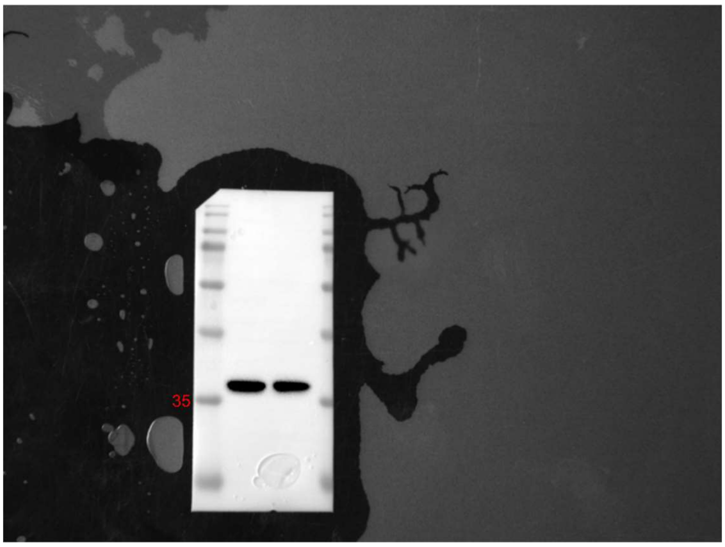

Figure 8F(CDK7)

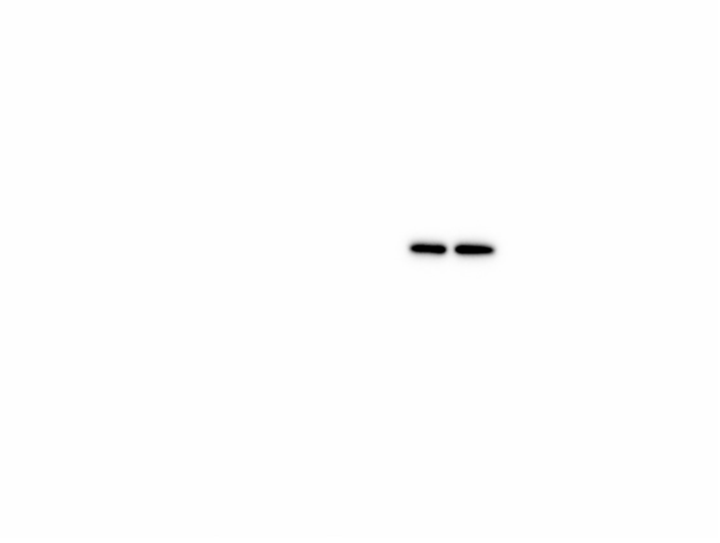

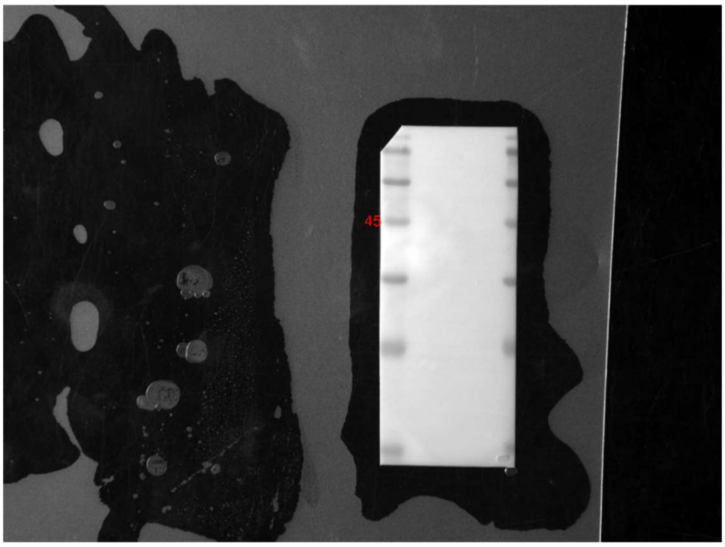

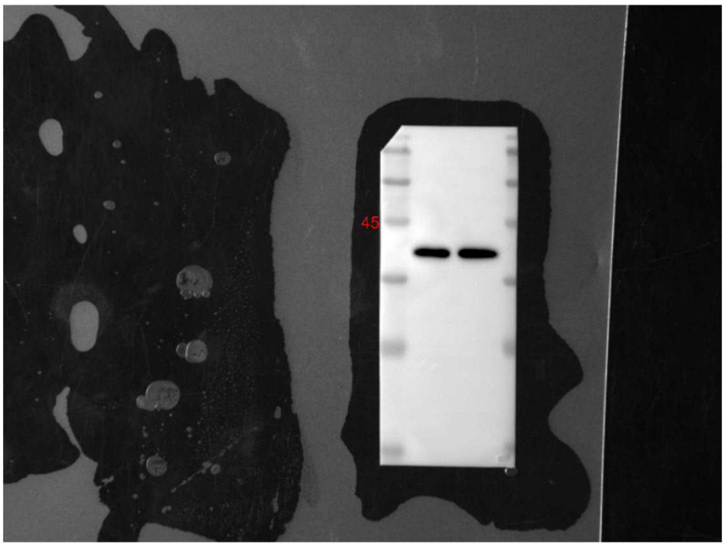

Figure 8F(CDK9)

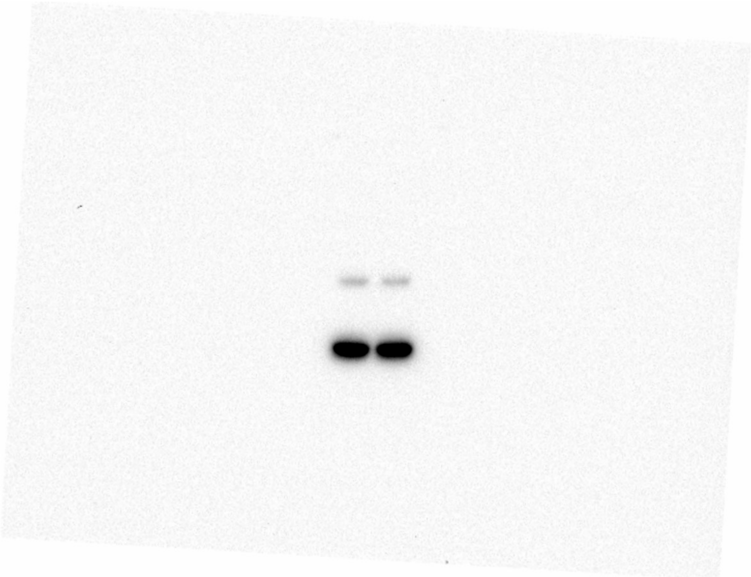

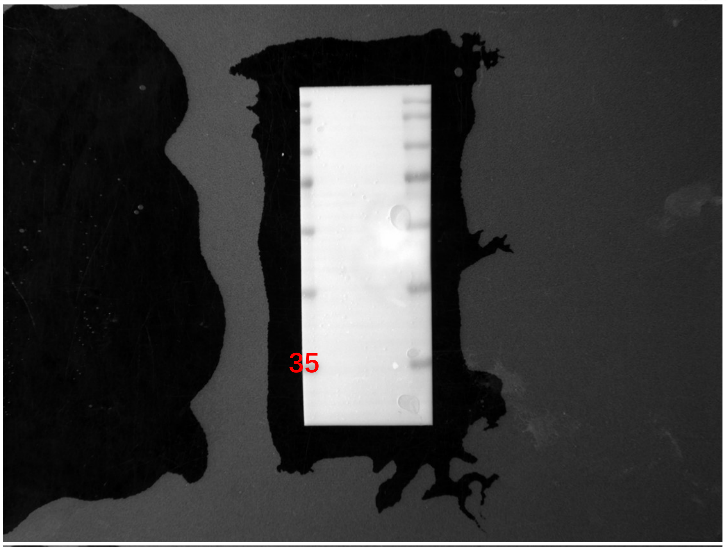

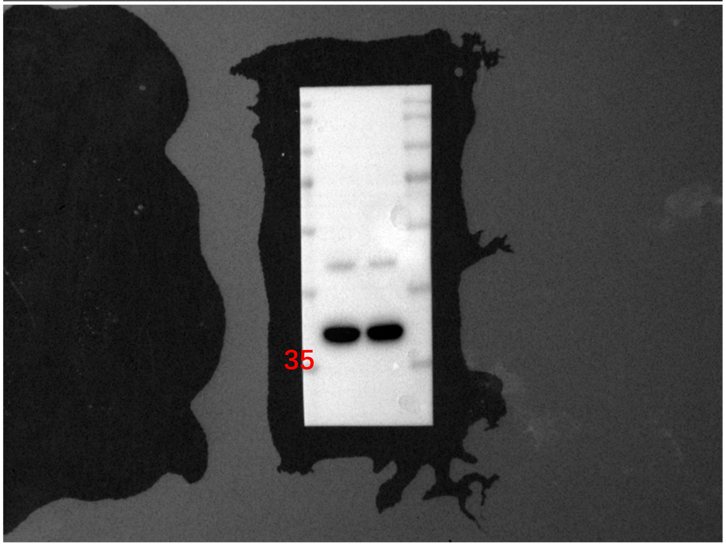

Figure 8F(Tubulin)

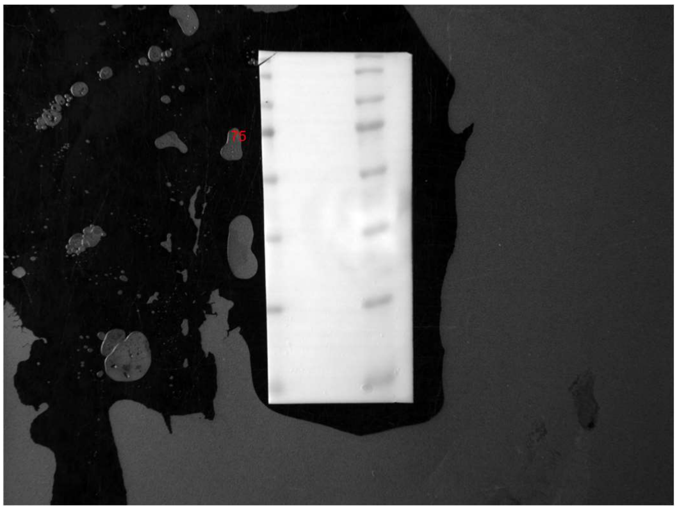

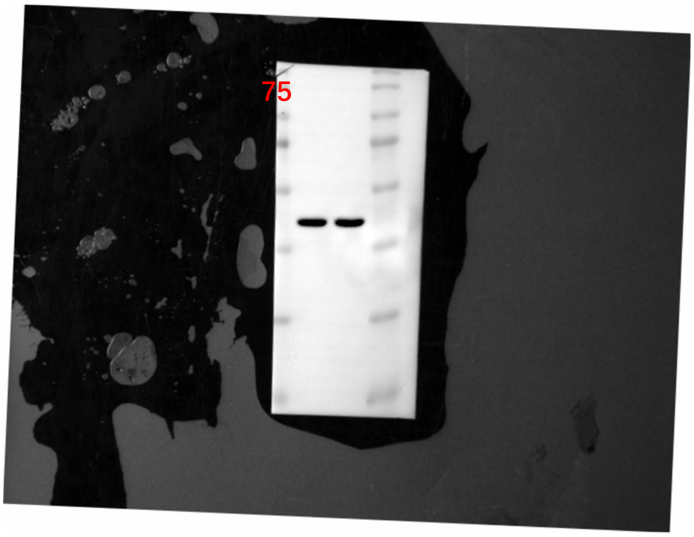

Figure 8F(P65)

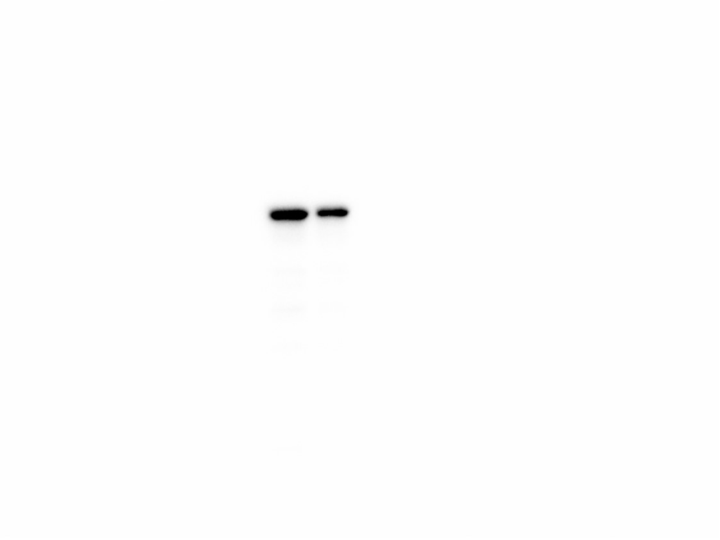

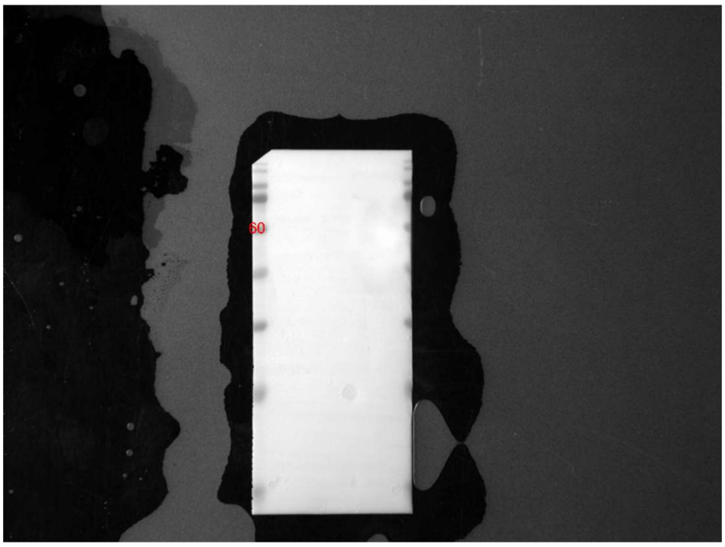

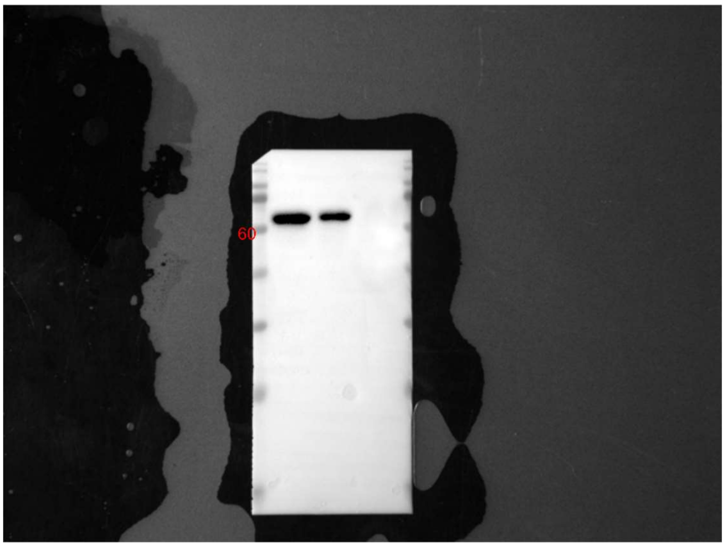

Figure 8F(BCL2)

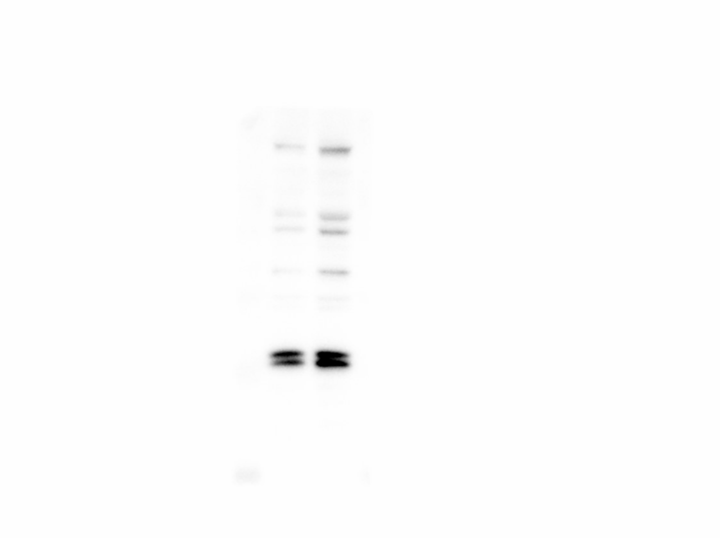

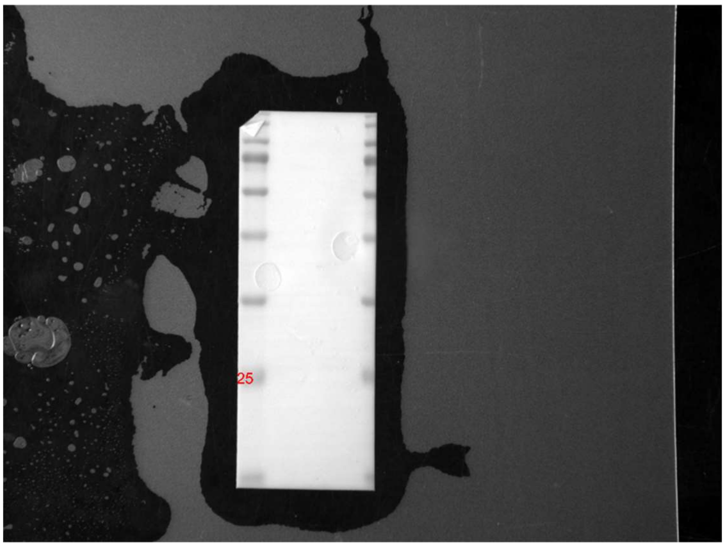

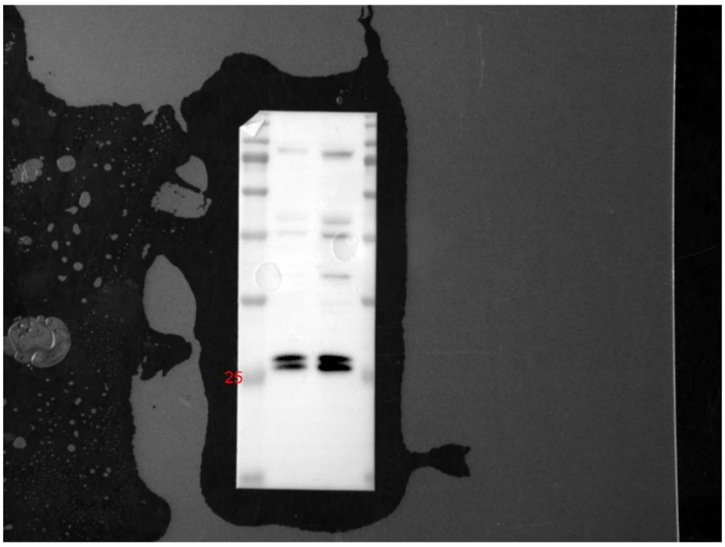

Figure 8F(Caspase3)

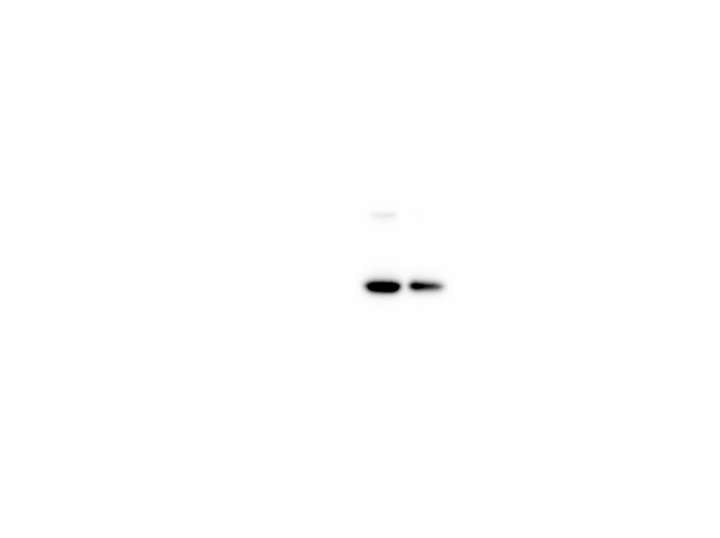

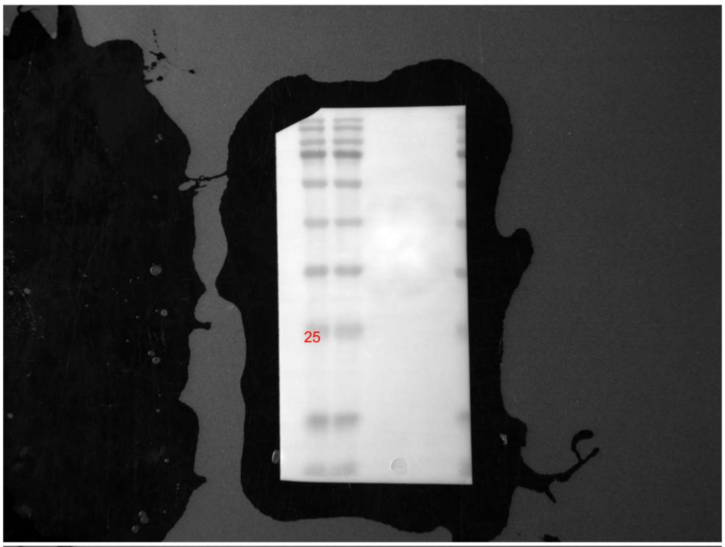

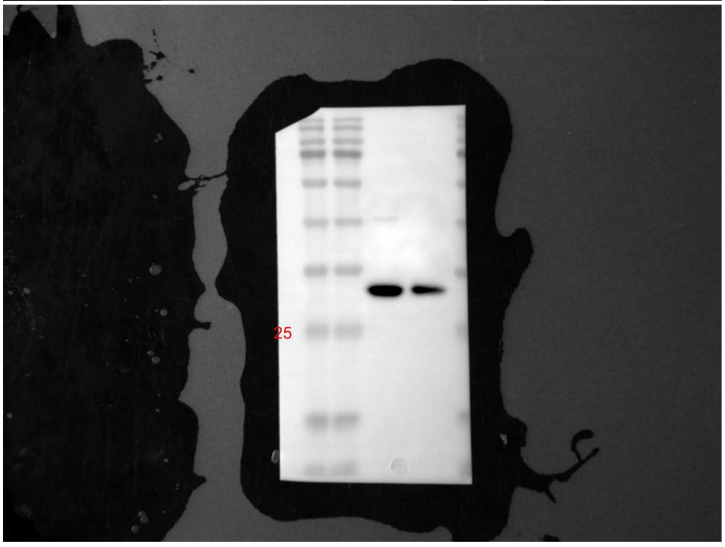

Figure 8F(p53)

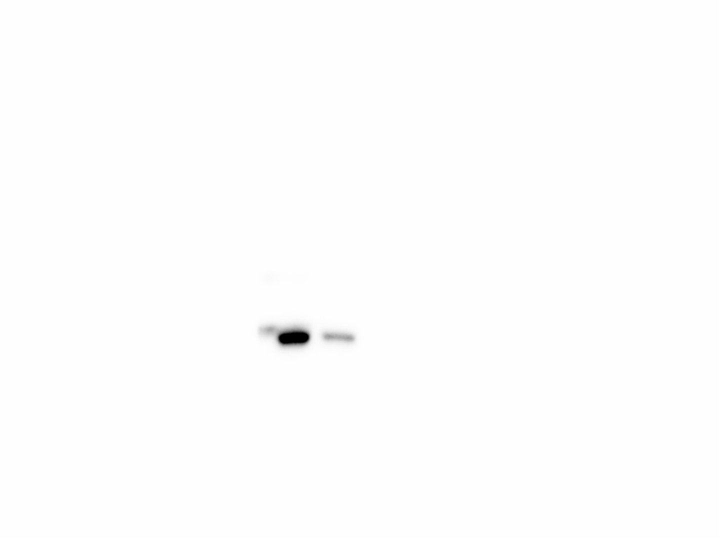

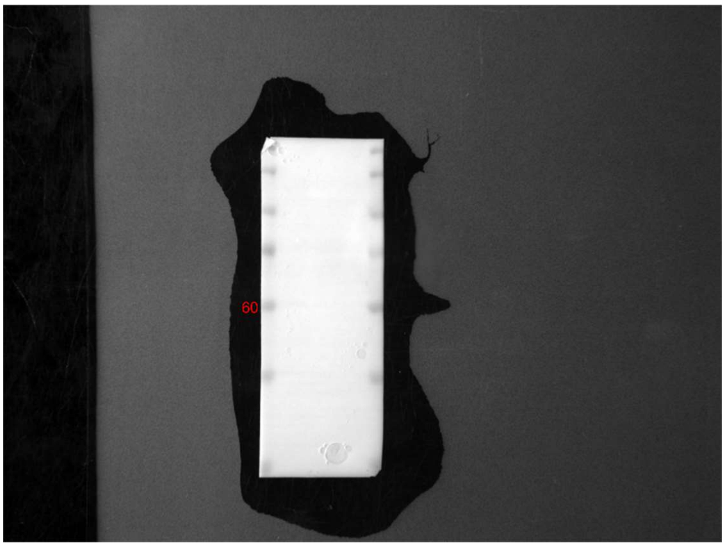

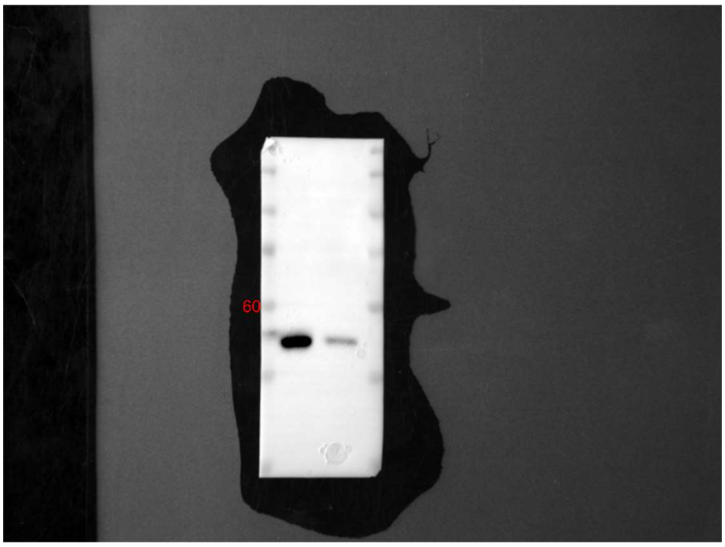

Figure 8F(P-Erk)

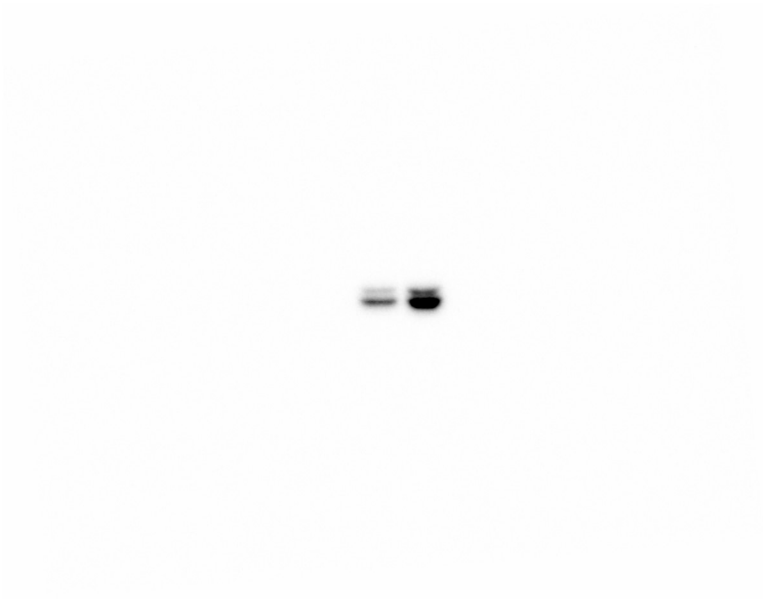

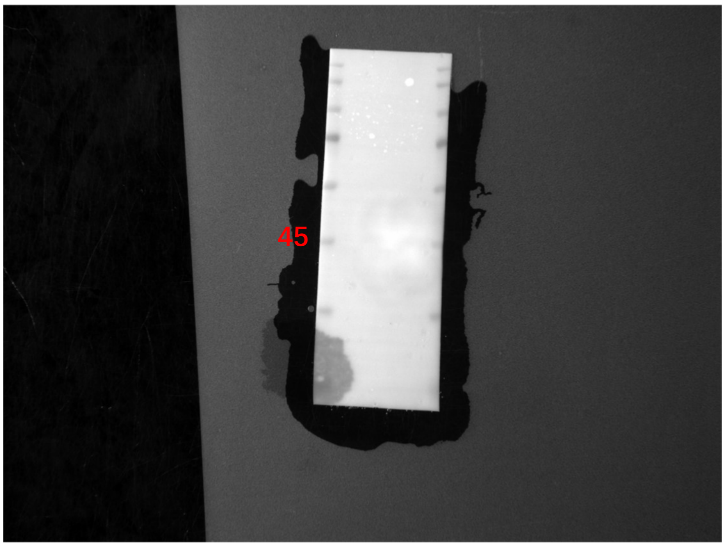

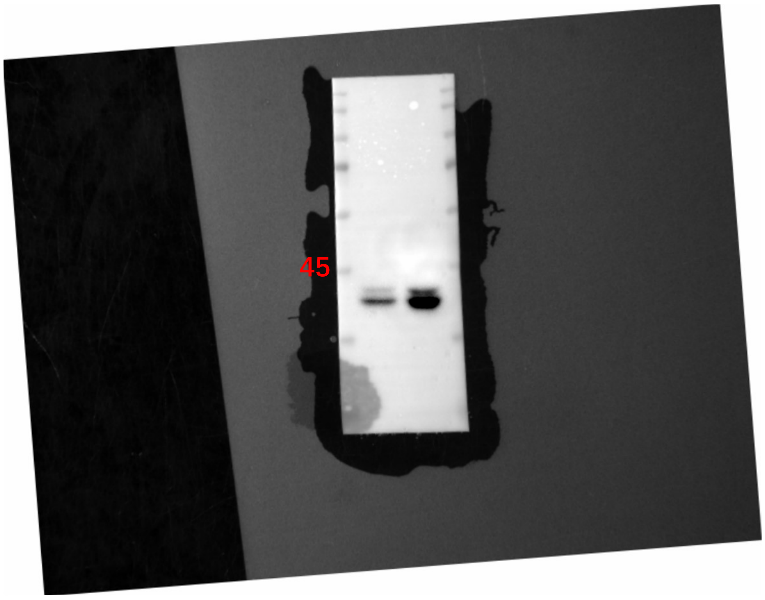

Figure 8F(ERK)

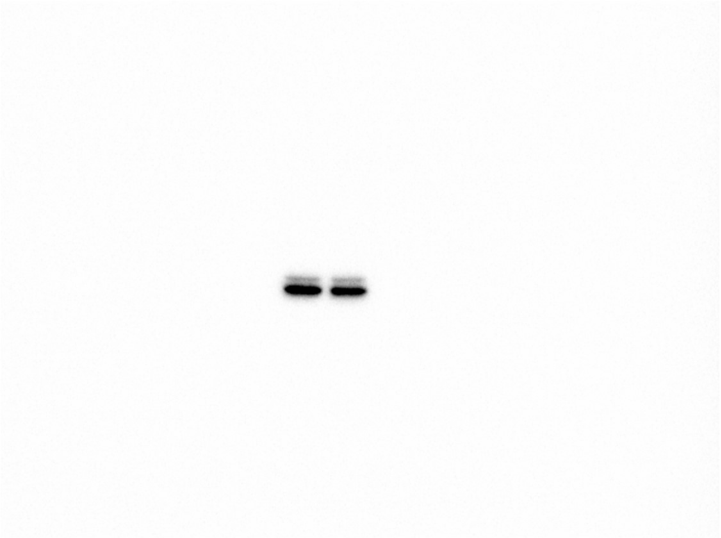

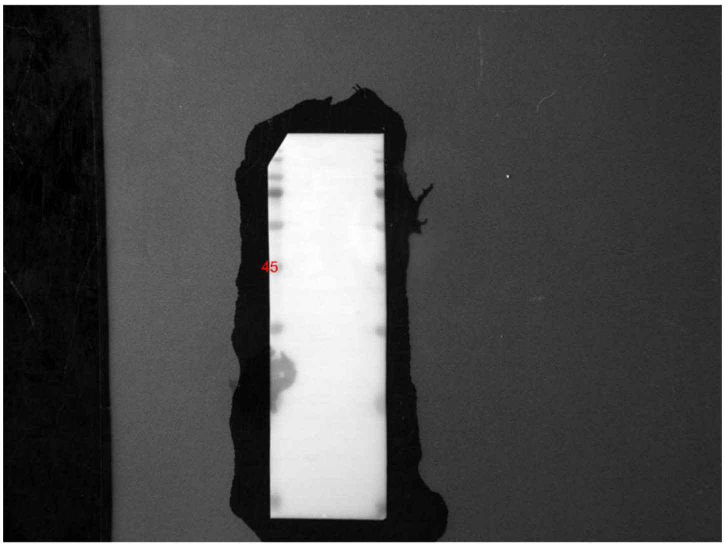

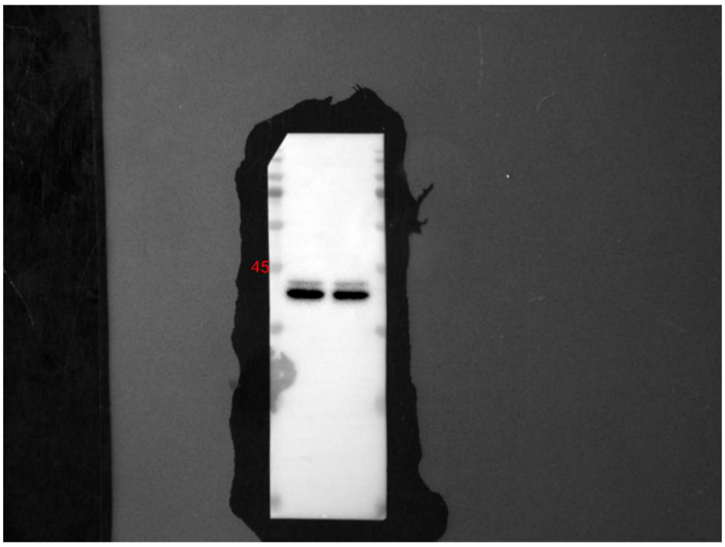

Figure 8F(Tubulin)

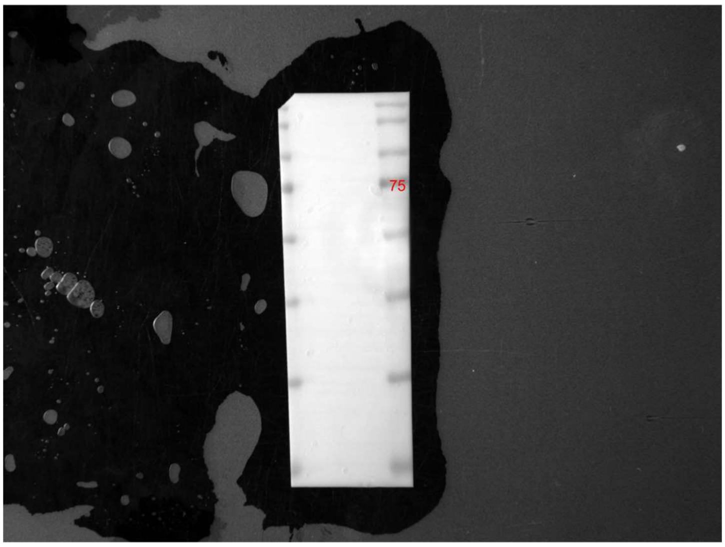

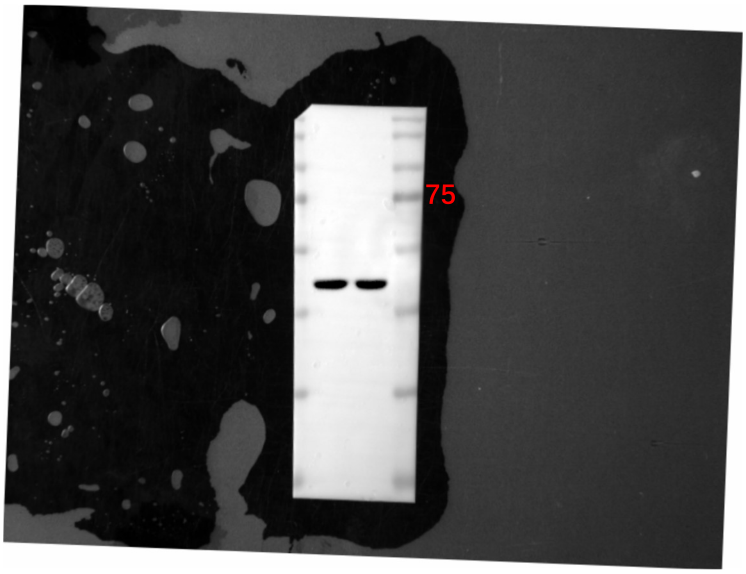

Figure 8G(CDK1)

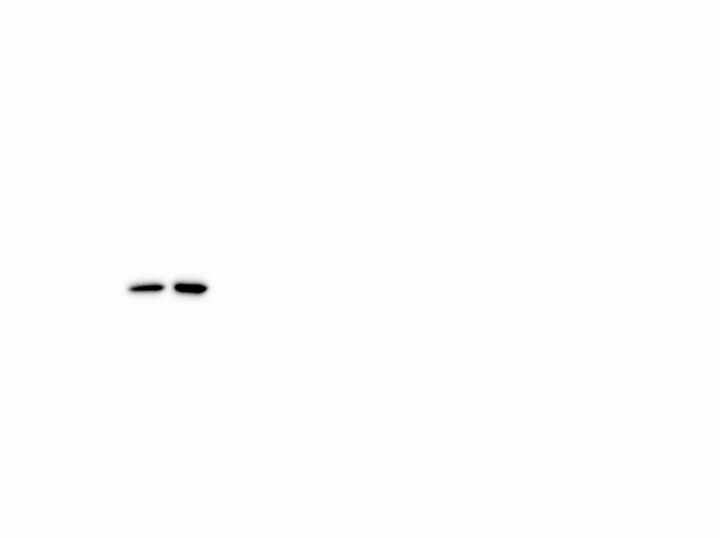

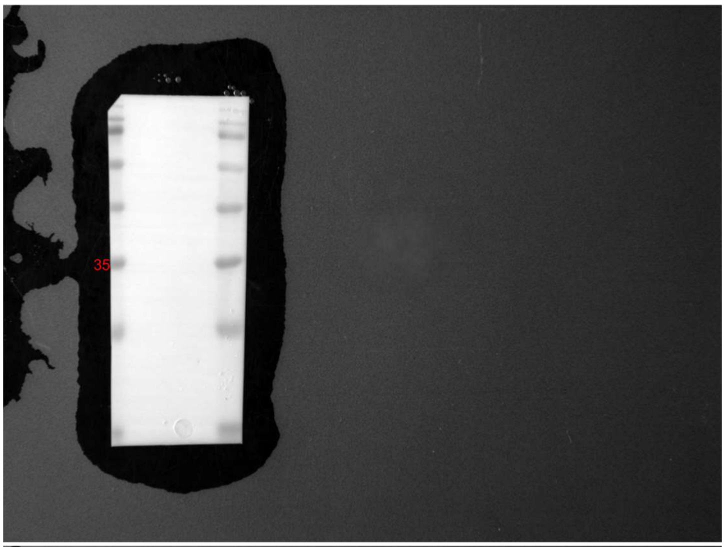

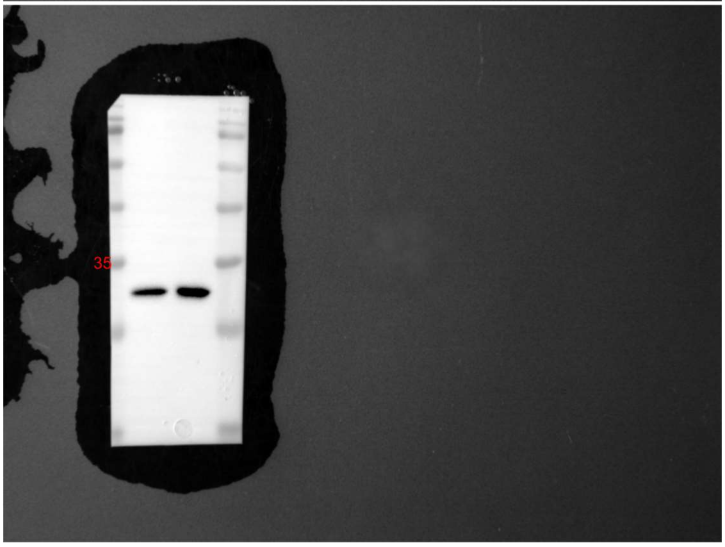

Figure 8G(CDK2)

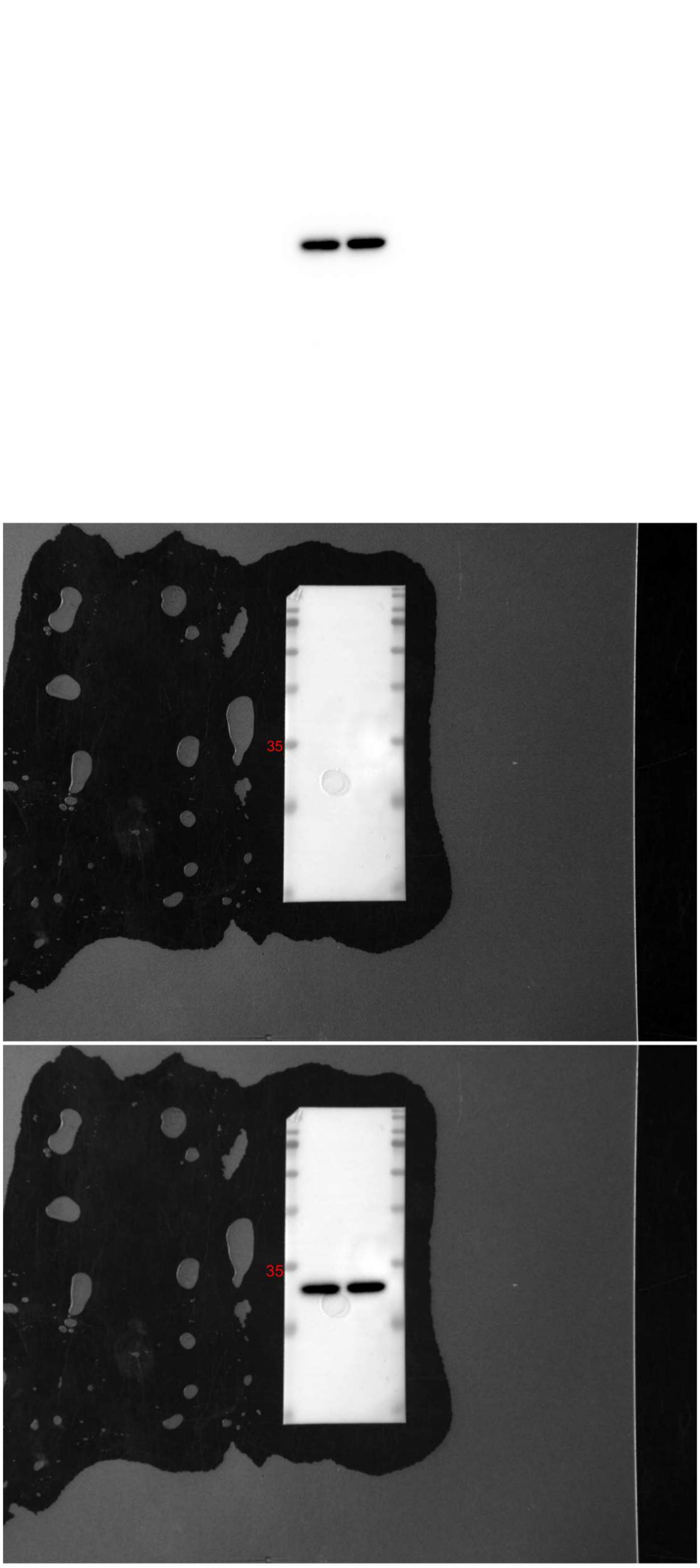

Figure 8G(CDK4)

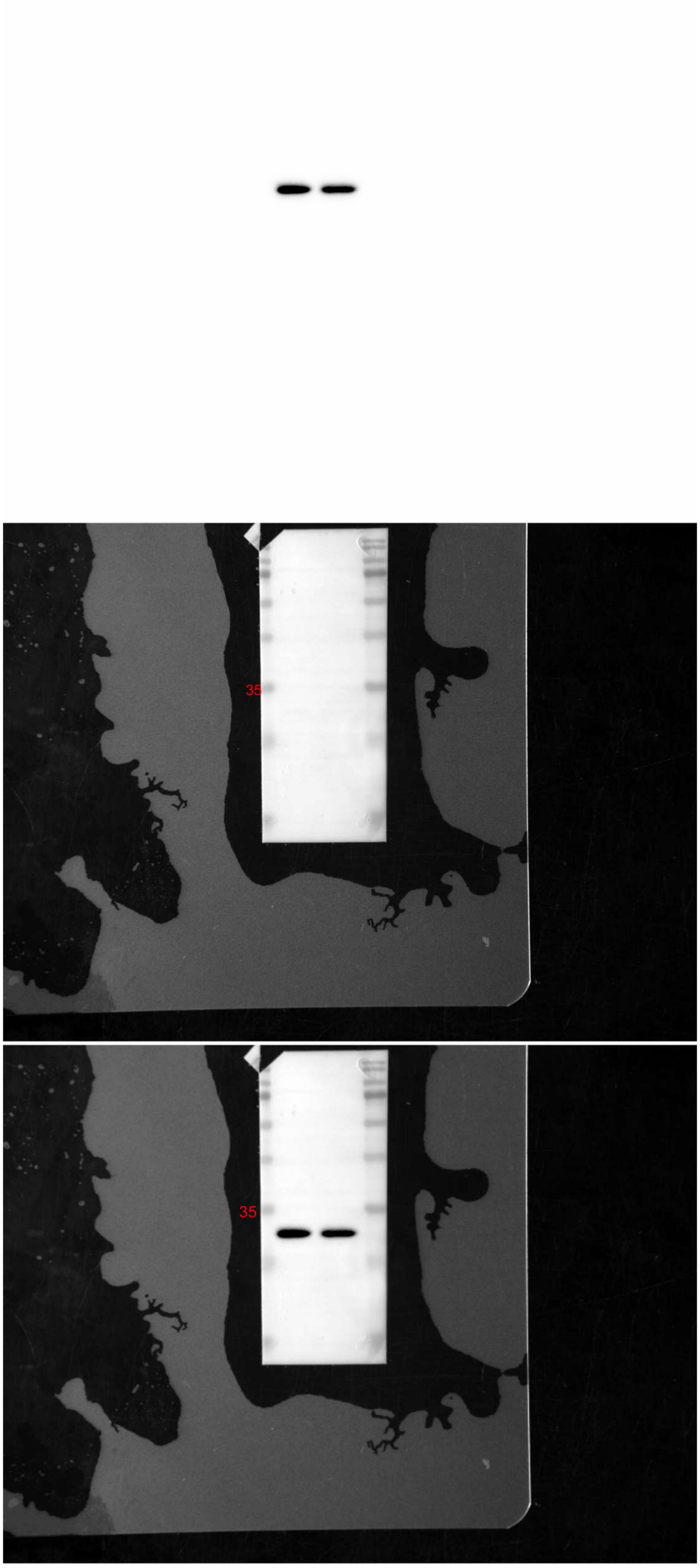

Figure 8G(CDK6)

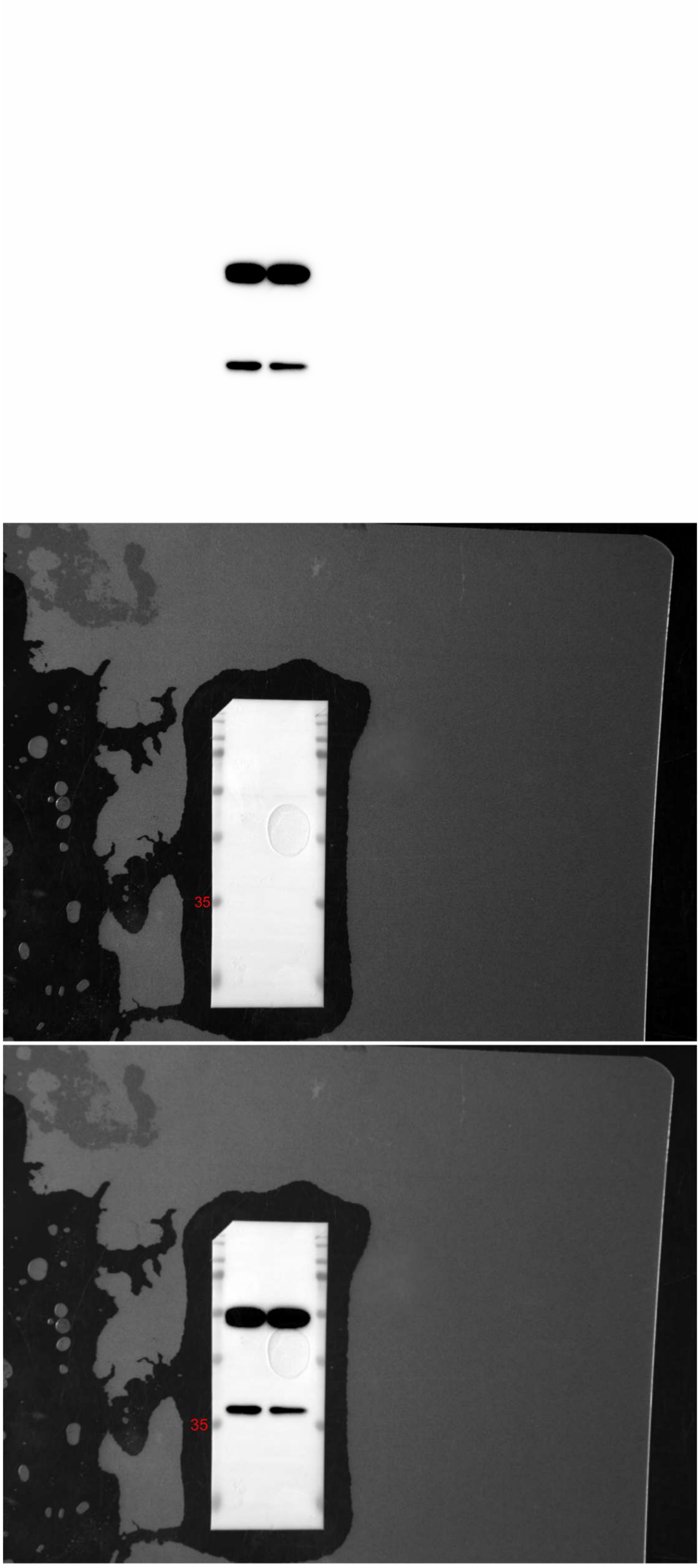

Figure 8G(CDK7)

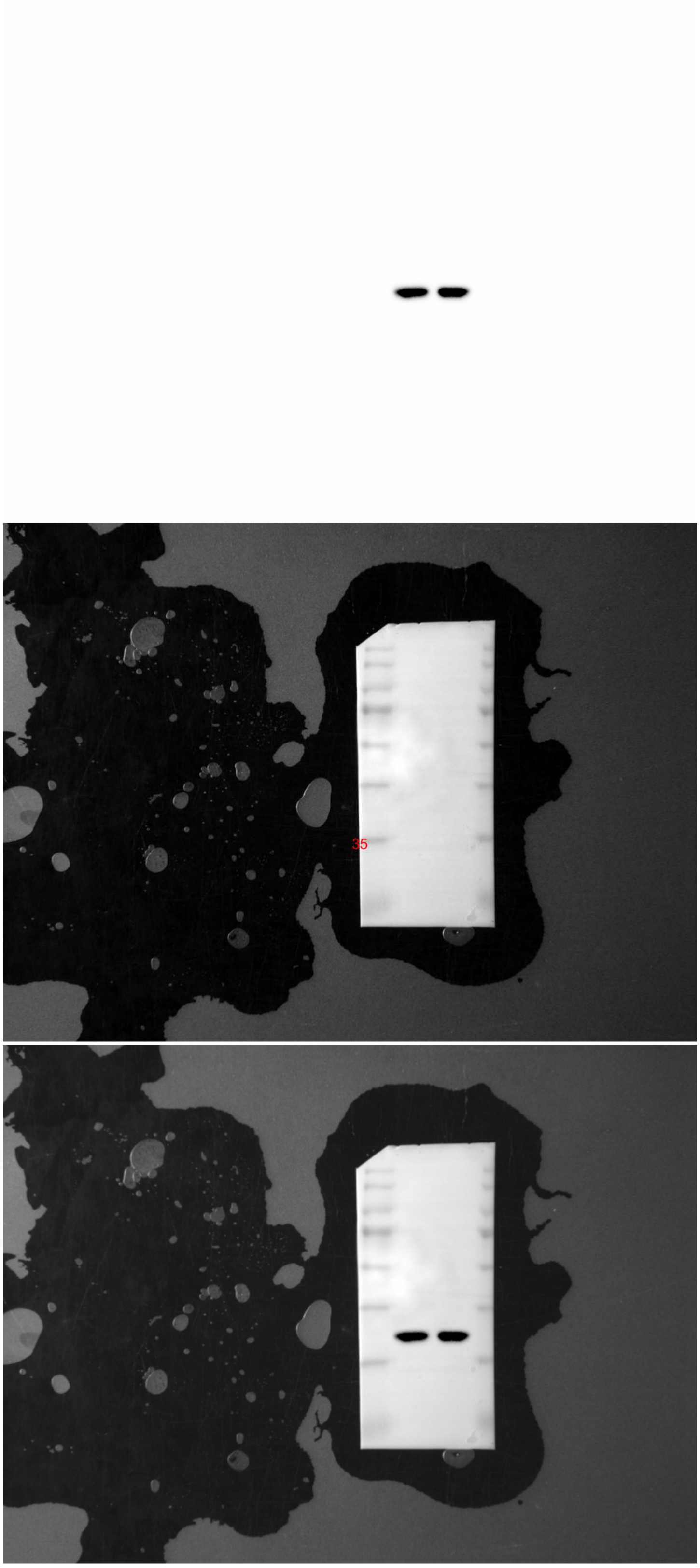

Figure 8G(CDK9)

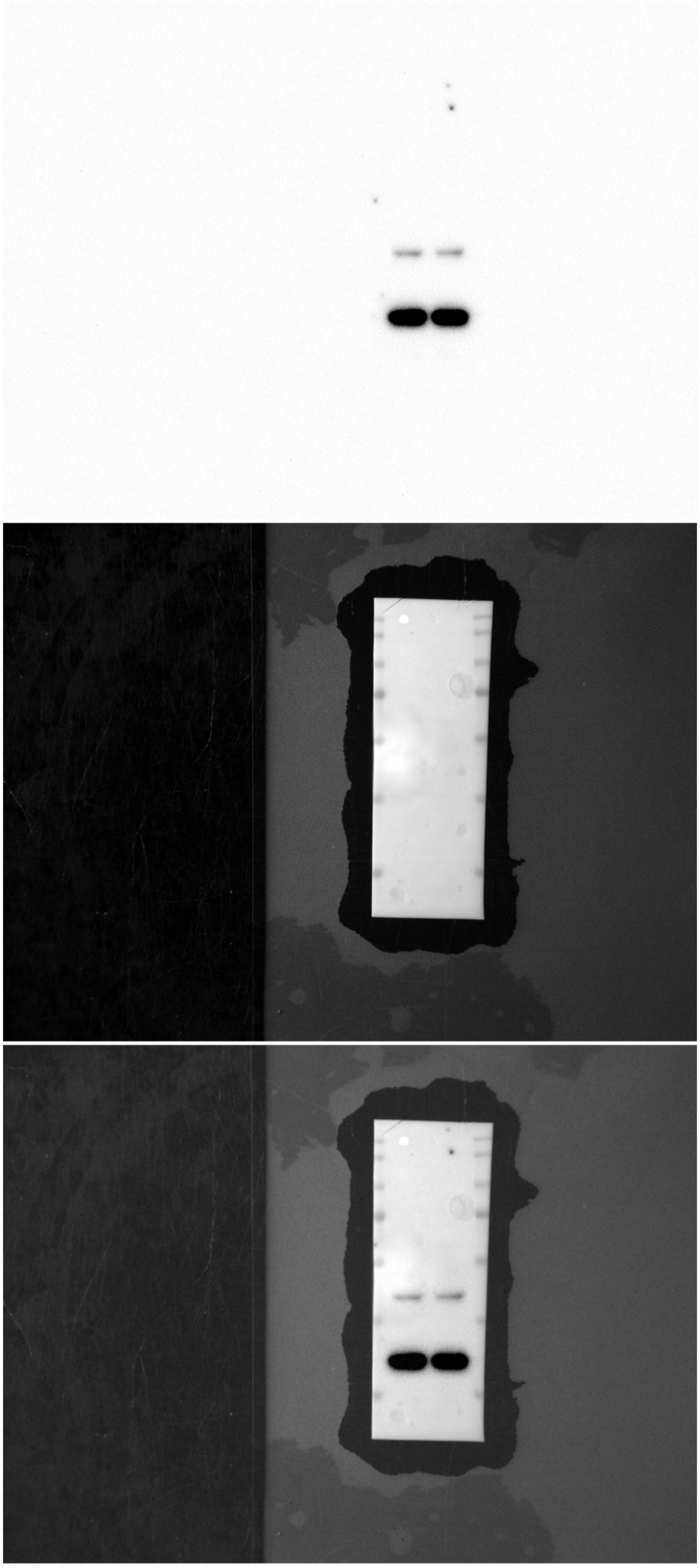

Figure 8G(tubulin)

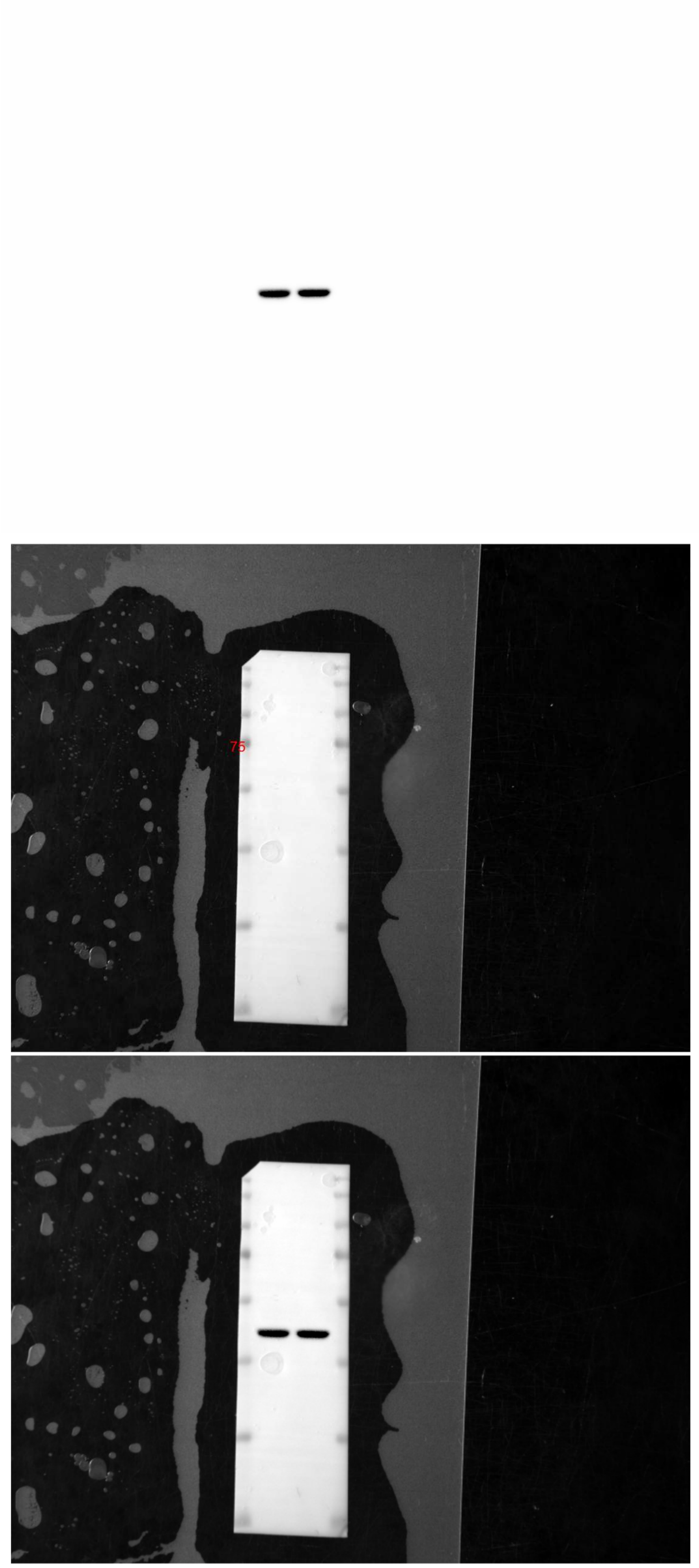

Figure 8G(P65)

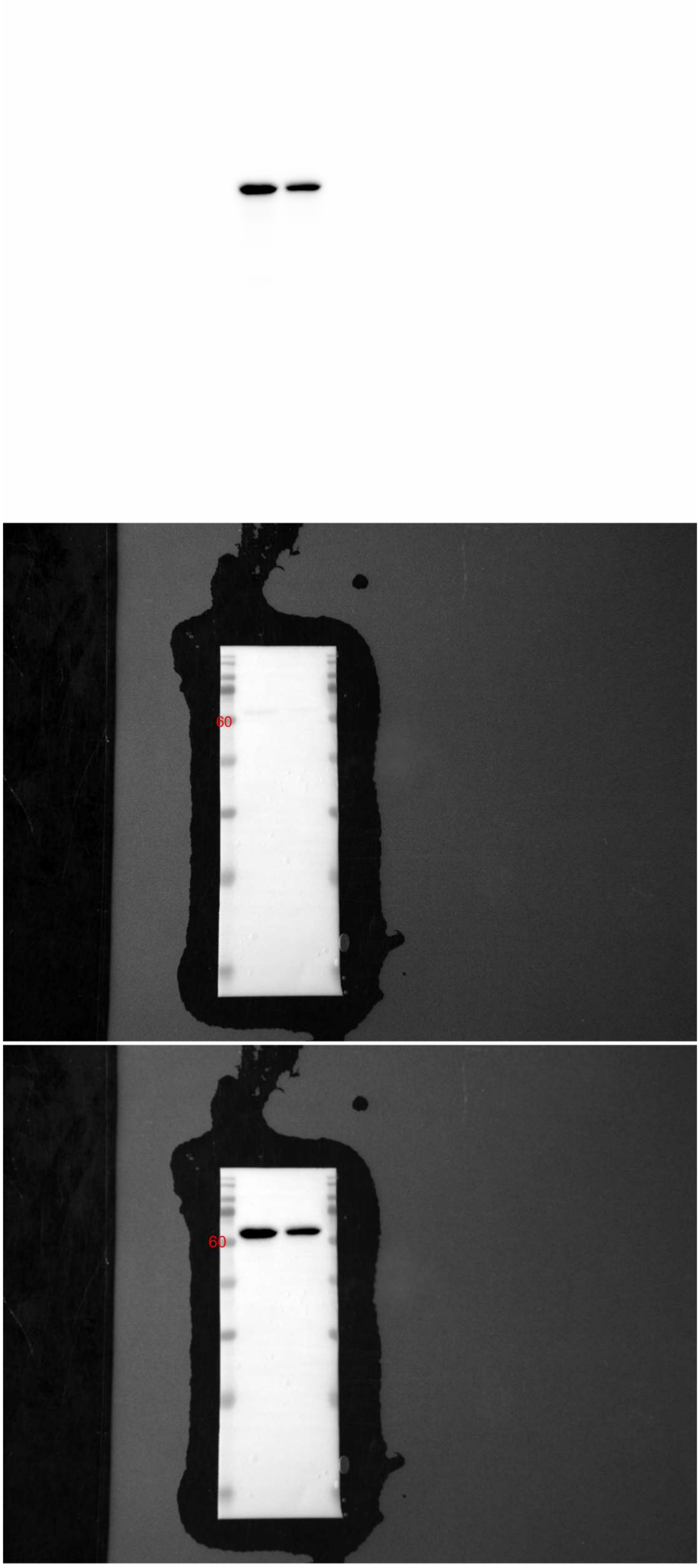

Figure 8G(BCL2)

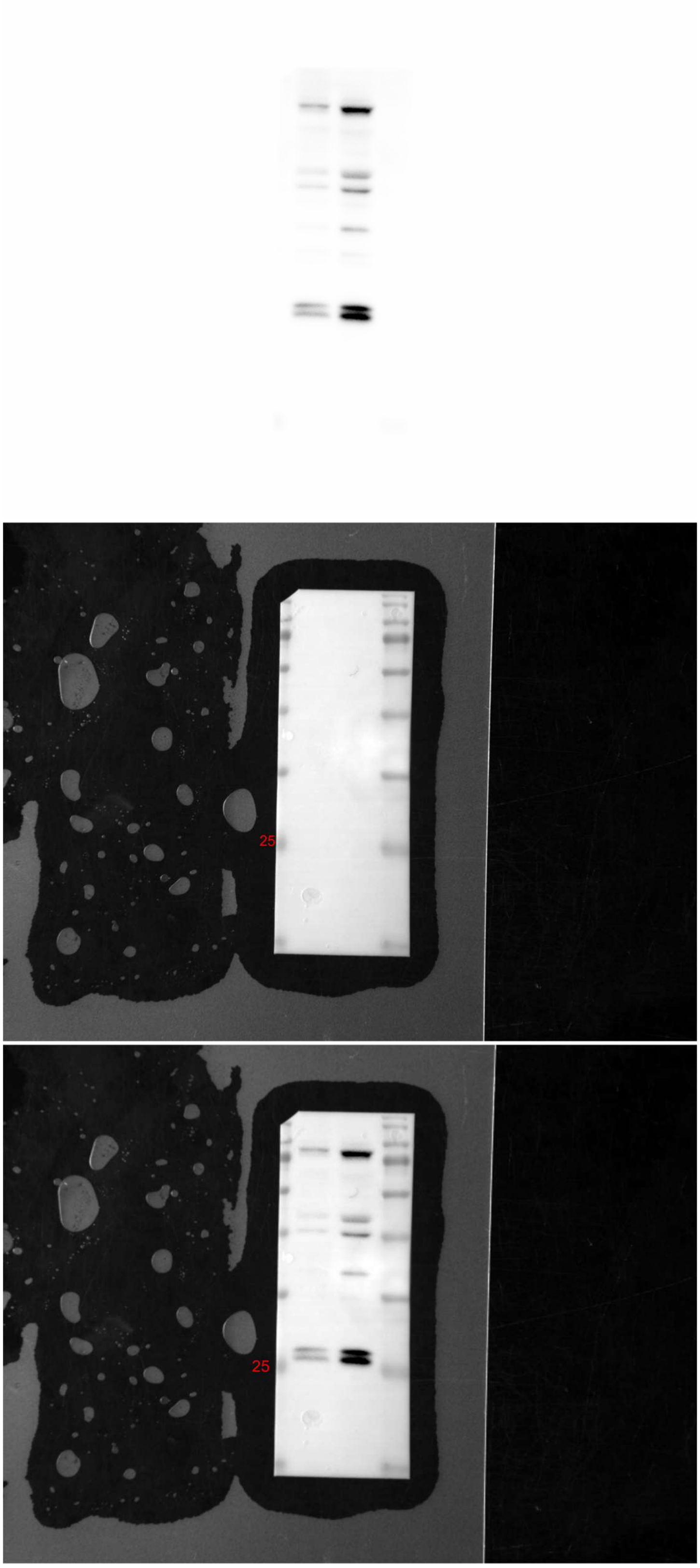

Figure 8G(Caspase3)

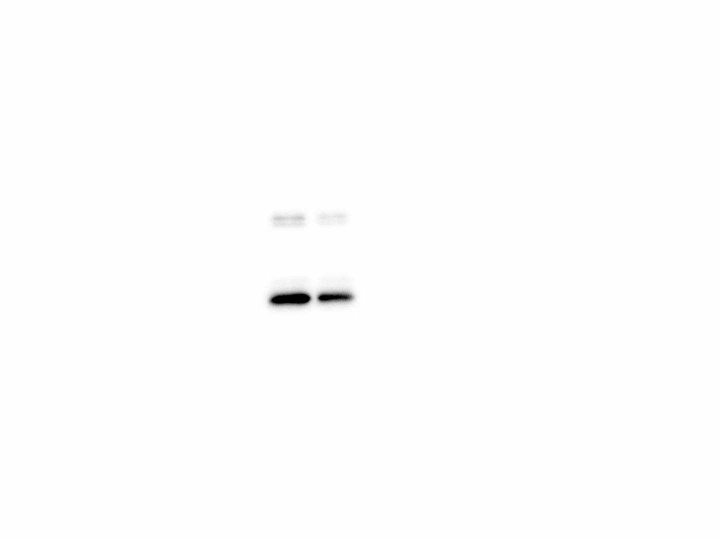

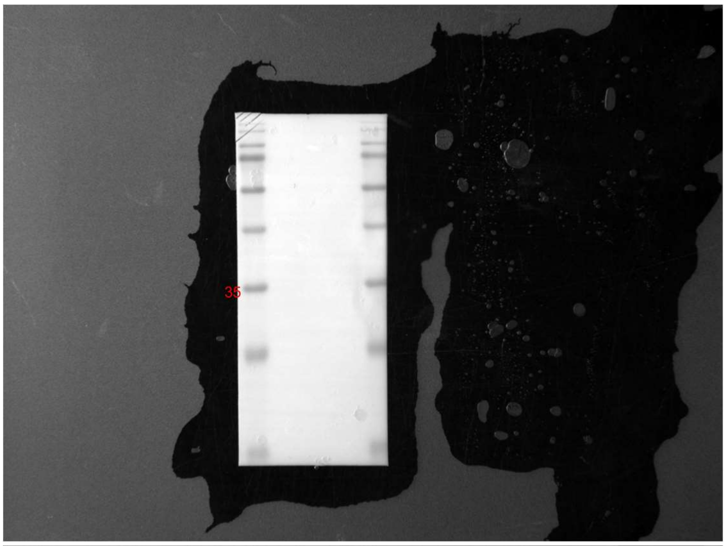

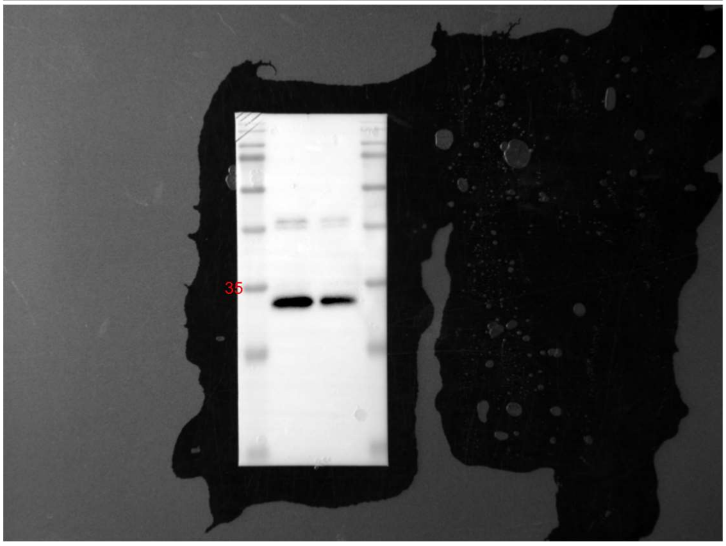

Figure 8G(P53)

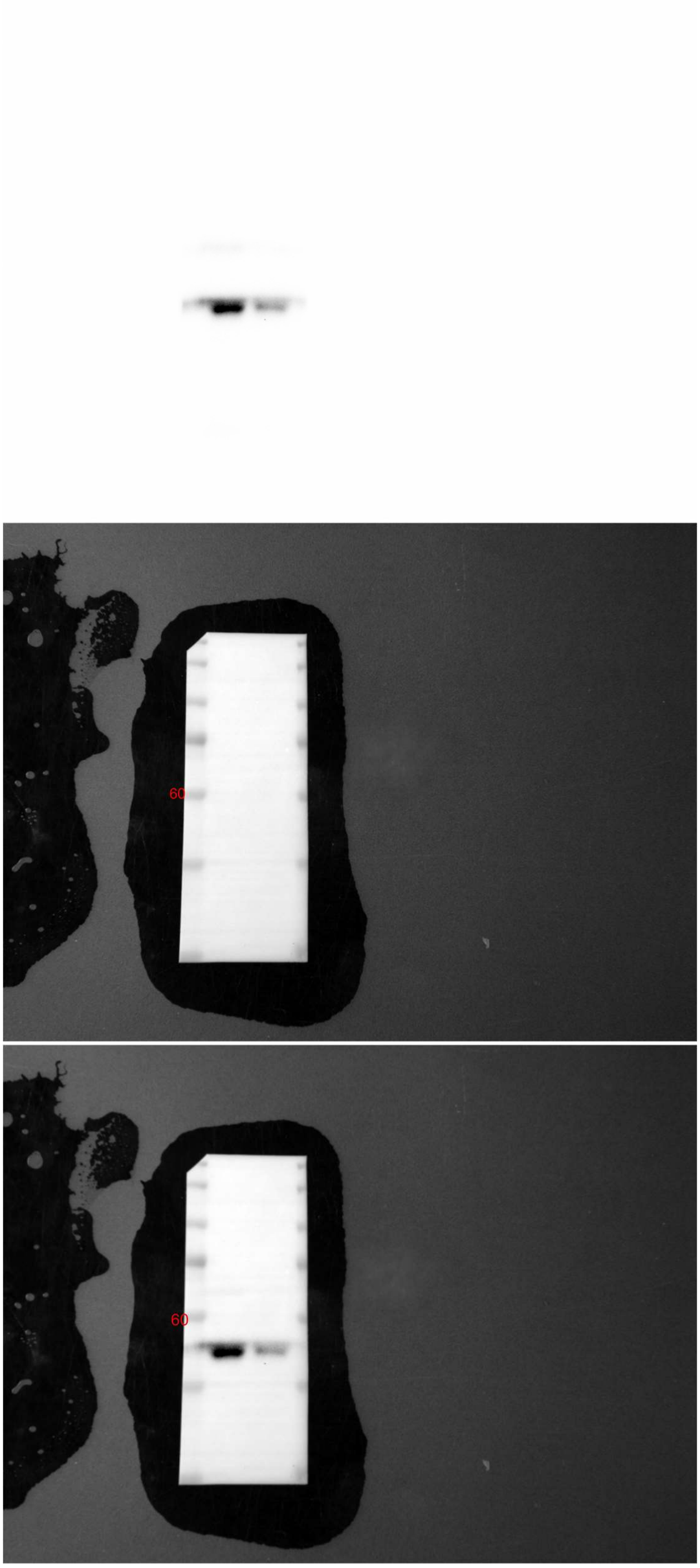

Figure 8G(P-ERK)

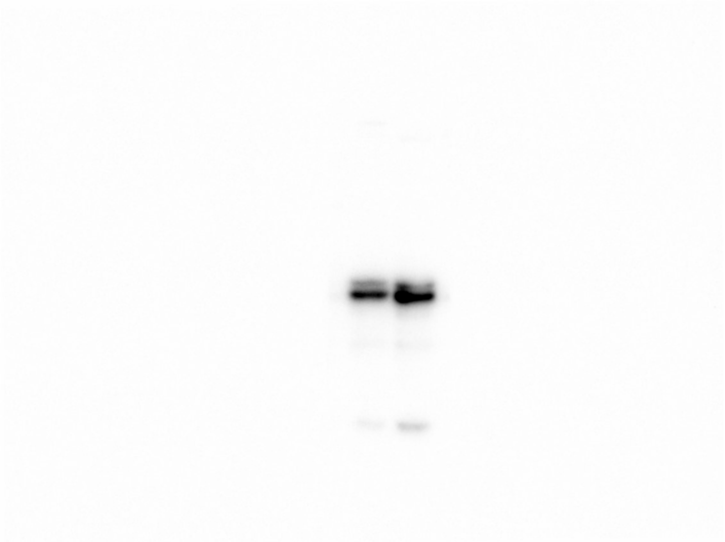

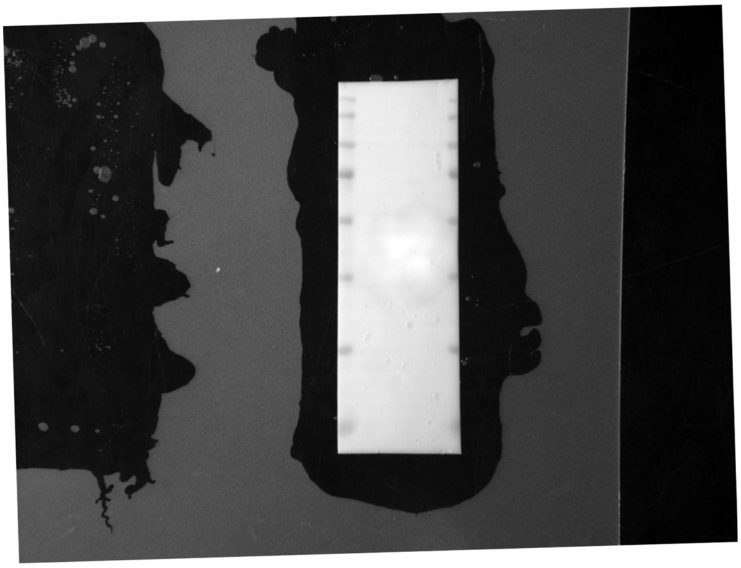

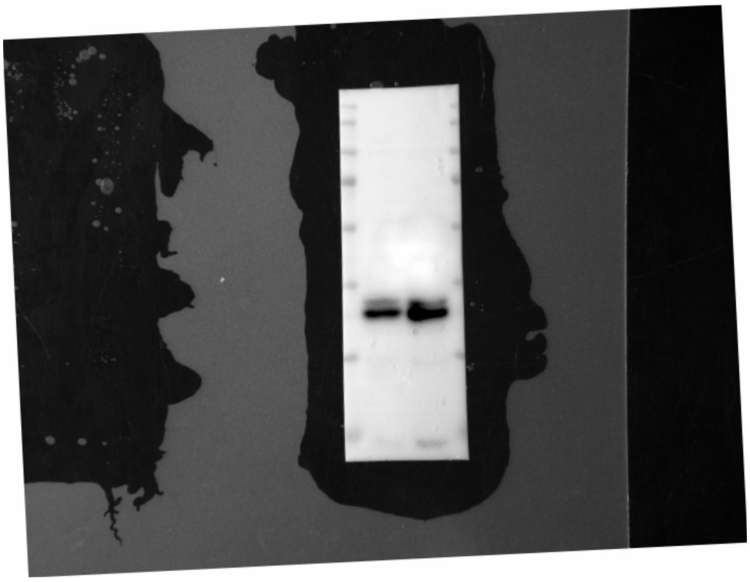

Figure 8G(ERK)

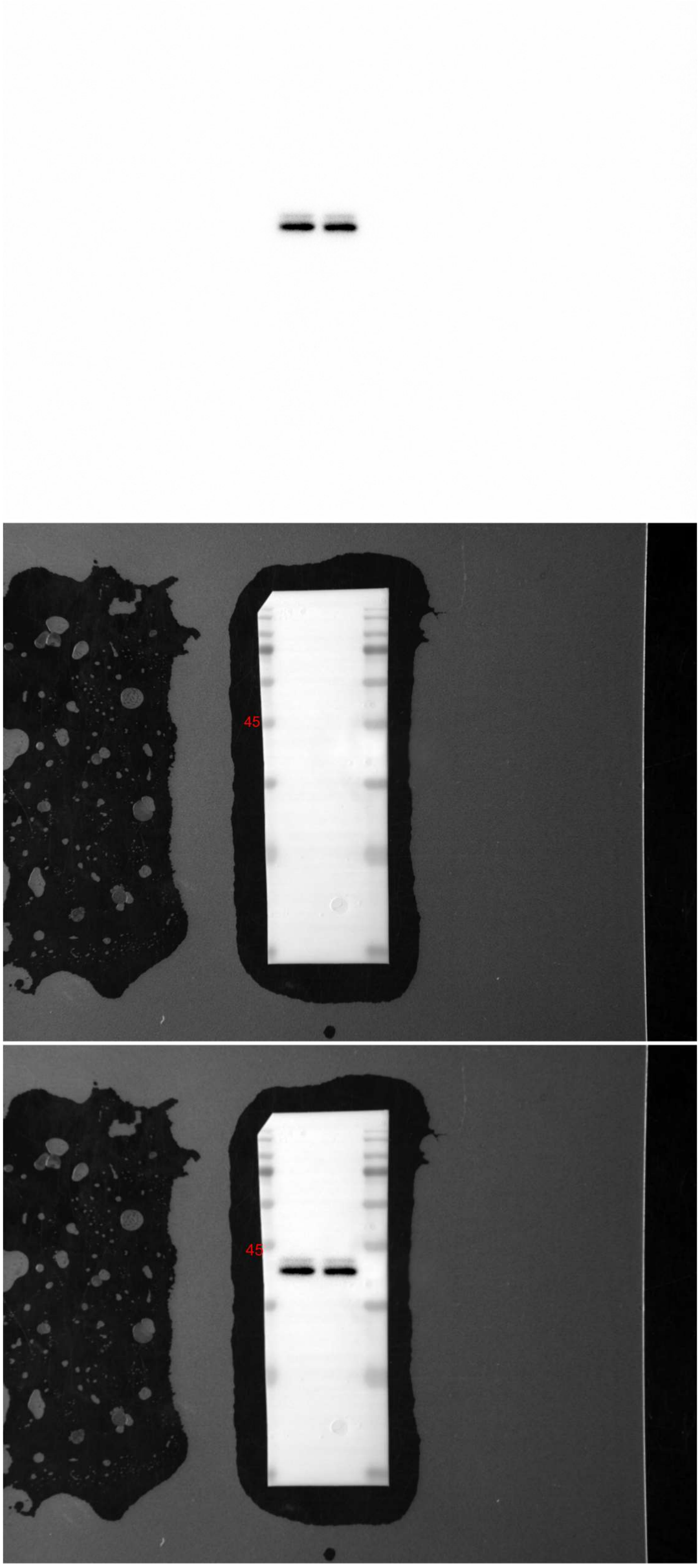

Figure 8G(TUBULIN)

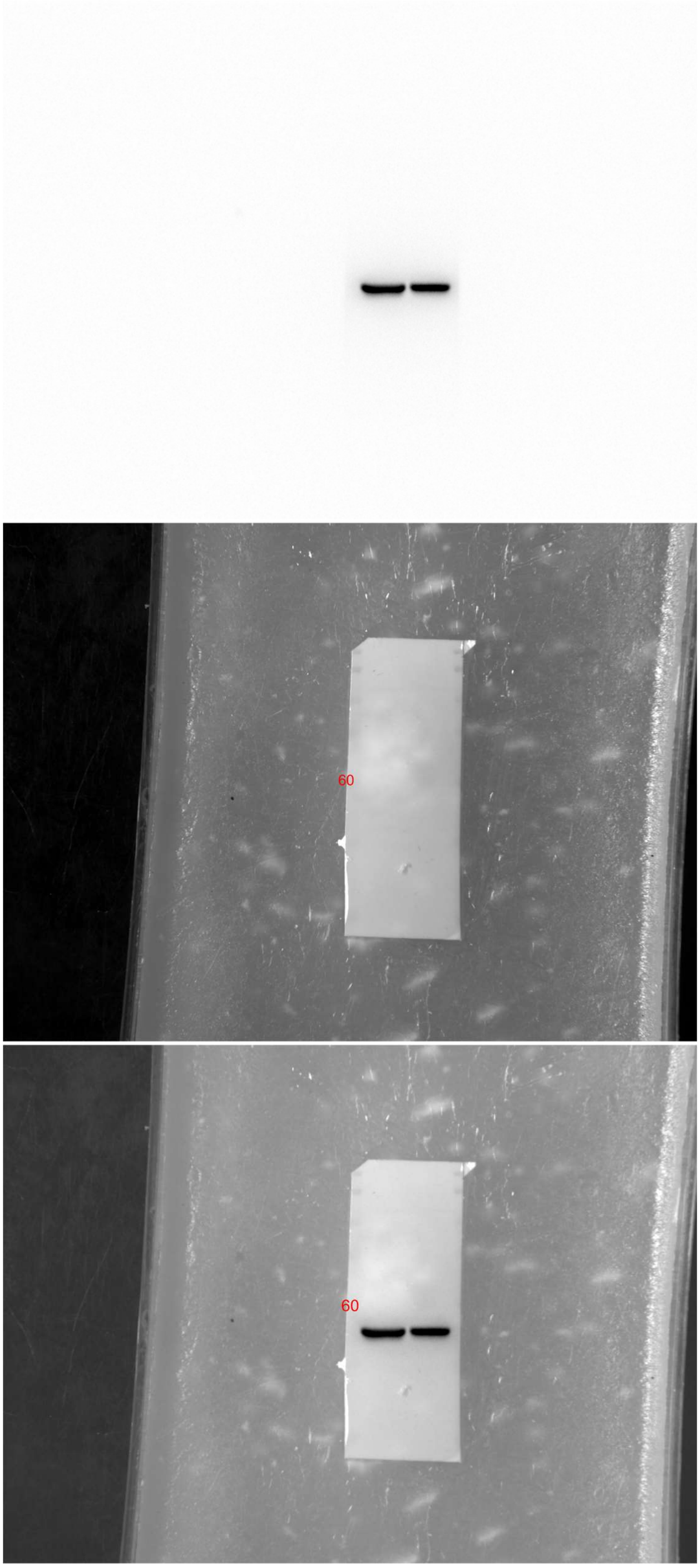

Supplement: Supplementary file 2 — Supplementary Figures. [file 41598_2023_41343_MOESM2_ESM.pdf]
