# Supplementary figures and images for: HMGCS2 serves as a potential biomarker for inhibition of renal clear cell carcinoma growth
Source: Sci Rep. 2023 Sep 5;13:14629. doi: 10.1038/s41598-023-41343-7 (PMC10480187; doi:10.1038/s41598-023-41343-7)

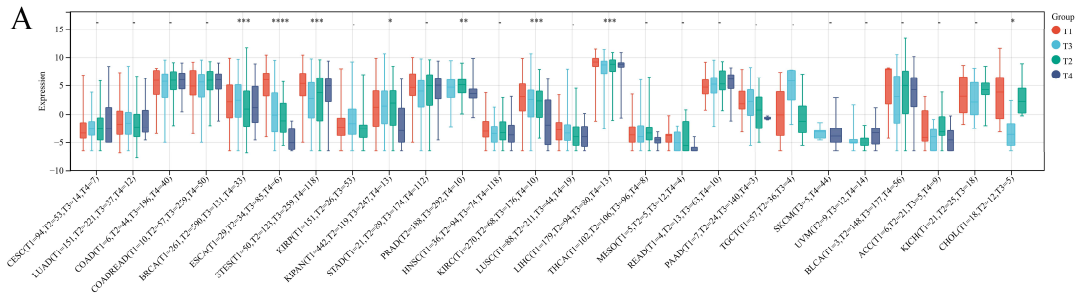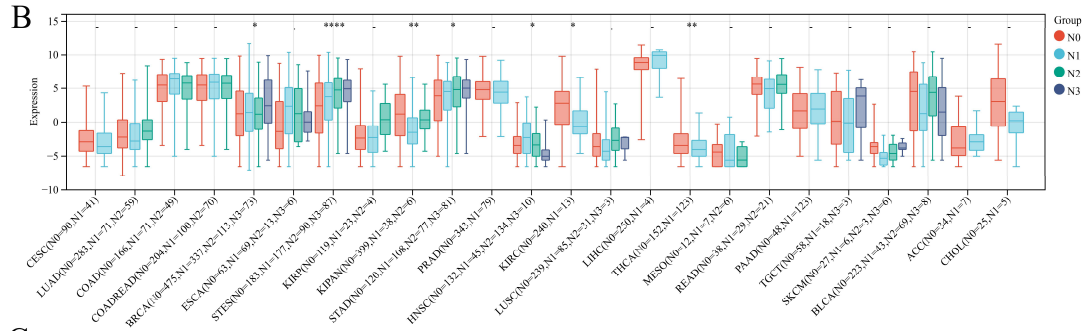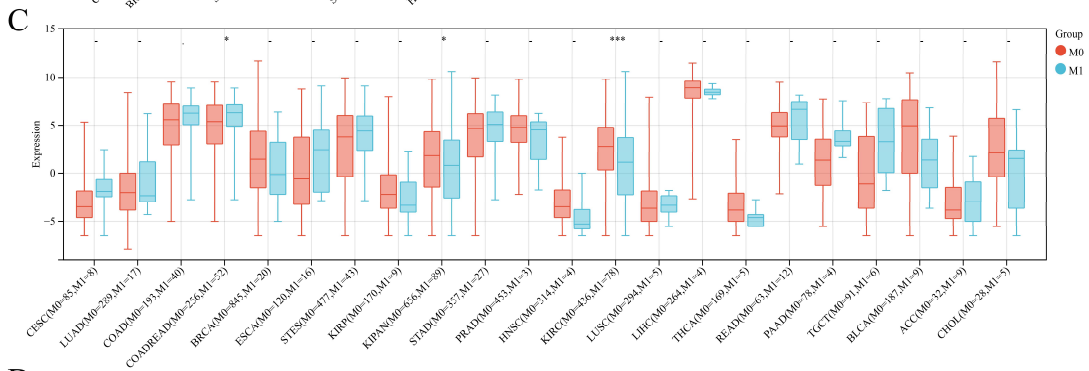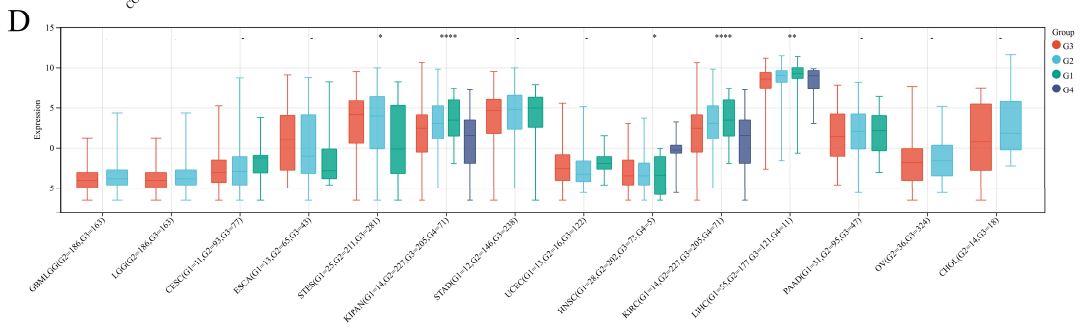

Supplement: Supplementary file 3 — Supplementary Figure 1. [file 41598_2023_41343_MOESM3_ESM.pdf]

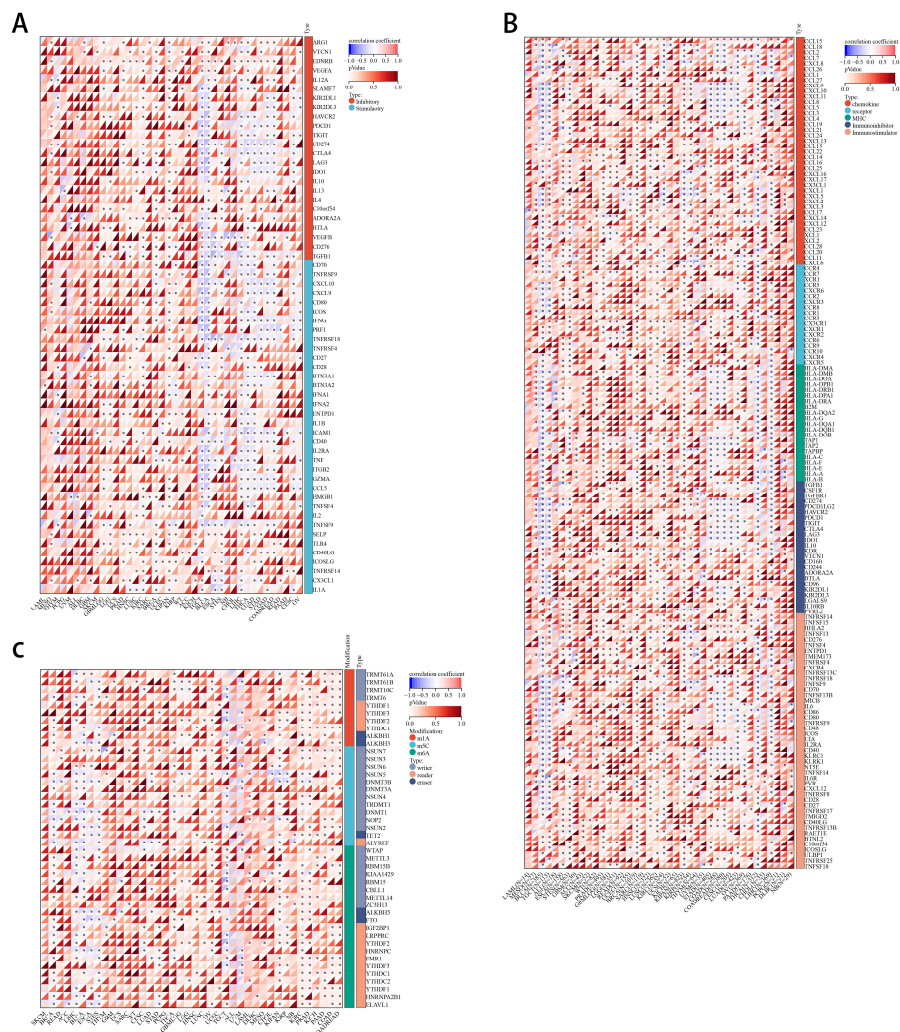

Supplement: Supplementary file 4 — Supplementary Figure 2. [file 41598_2023_41343_MOESM4_ESM.pdf]
